# Cardiac mechanics modeling: recent developments and current challenges

**Published:** 2025-09-09

**Authors:** Aaron L. Brown, Ju Liu, Daniel B. Ennis, Alison L. Marsden

**Affiliations:** a Department of Mechanical Engineering, Stanford University, Stanford, CA, USA; b Stanford Cardiovascular Institute, Stanford, CA, USA; c Department of Mechanics and Aerospace Engineering, Southern University of Science and Technology, Shenzhen, Guangdong, China; d Department of Radiology, Stanford University, Stanford, CA, USA; e Department of Pediatrics (Cardiology), Stanford University, Stanford, CA, USA; f Department of Bioengineering, Stanford University, Stanford, CA, USA

**Keywords:** Cardiac digital twin, Cardiac mechanics, Patient-specific modeling, Multiphysics modeling, Computational cardiology

## Abstract

Patient-specific computational models of the heart are powerful tools for cardiovascular research and medicine, with demonstrated applications in treatment planning, device evaluation, and surgical decision-making. Yet constructing such models is inherently difficult, reflecting the extraordinary complexity of the heart itself. Numerous considerations are required, including reconstructing the anatomy from medical images, representing myocardial mesostructure, capturing material behavior, defining model geometry and boundary conditions, coupling multiple physics, and selecting numerical methods. Many of these choices involve a tradeoff between physiological fidelity and modeling complexity. In this review, we summarize recent advances and unresolved questions in each of these areas, with particular emphasis on cardiac tissue mechanics. We argue that clarifying which complexities are essential, and which can be safely simplified, will be key to enabling clinical translation of these models.

## 1 Introduction

Patient-specific, multiphysics computational models of the heart hold the potential to revolutionize cardiovascular medicine by enabling the creation of digital twins—virtual replicas of an individual’s heart continuously updated with information from the physical twin [1]. Cardiac digital twins can support clinicians in developing personalized treatment strategies tailored to each patient’s unique anatomy and physiology. Beyond clinical applications, digital twin technology offers powerful tools for advancing research, by guiding the design of next-generation therapies and devices and by deepening our understanding of disease mechanisms. This review outlines the major steps in constructing heart models, delves into open questions and current debates, and summarizes recent advancements, with a primary focus on cardiac tissue mechanics.

To be clear about terminology, true digital heart twins, for which the bidirectional flow of information is essential, are rare in the literature. Most current efforts construct “passive” digital twins [2], or “digital snapshots” [3], built from patient data, but not dynamically updated. These models nonetheless lay the essential groundwork for future true digital twins, while ongoing work is exploring continuous data collection through wearables and other sensors, as well as new frameworks for dynamic integration of data into the digital twin [1, 3]. For the remainder of this review, we use the term personalized computational heart model to refer to such precursor digital twins [2], reserving the term digital twin itself for the aspirational tool of the future.

Computational cardiac modeling has already demonstrated significant utility in treatment planning, device evaluation, and surgical decision-making. Electromechanical simulations have been employed to evaluate novel pacing strategies and their effect on cardiac pump function, comparisons that are impractical to perform directly in clinical settings [4]. In related work, patient-specific models were used to optimize cardiac resynchronization therapy (CRT) by simulating various lead placements and computing key biomarkers such as dP/dt_max_, ejection fraction, and stroke work [5]. In structural heart interventions, simulations have elucidated the mechanisms by which devices like the Parachute^®^ implant reduce end-diastolic myofiber stress and potentially reverse post-infarct remodeling [6]. Additionally, hybrid experimental-computational platforms have supported the evaluation of mechanical circulatory support devices such as the Jarvik 2000 pump for single-ventricle circulations [7]. Virtual surgical planning using patient-specific CFD models has been applied to optimize graft geometry in coronary artery bypass surgery [8] and to assist in transcatheter aortic valve replacement planning [9]. Finally, even purely anatomical 3D models, without any underlying physics simulation, can aid surgical planning, for example when designing an intracardiac baffle in double outlet right ventricle cases [10].

Beyond clinical applications, computational modeling provides a powerful framework for uncovering pathophysiological mechanisms that are otherwise difficult to probe. In hypertrophic cardiomyopathy (HCM), electrophysiology simulations have revealed that diffuse myocardial fibrosis significantly increases arrhythmogenic risk, suggesting that fibrosis burden may be a more informative predictor of sudden cardiac death than current clinical criteria [11]. Related work has evaluated multiple hypothesized contributors to impaired ventricular mechanics in HCM, including increased wall thickness, increased stiffness, decreased contractility, and fiber disarray [12]. Multiscale modeling that incorporates metabolic function has revealed the subcellular mechanisms by which 2’-deoxy-ATP improves cardiac function in heart failure patients [13]. Multiphysics modeling has also been used to explore how atrial myopathy contributes to stroke, demonstrating that fibrosis-mediated impairment of left atrial motion alters blood flow and promotes stasis in the left atrial appendage [14]. In developmental cardiology, computational studies have shown that in embryonic zebrafish hearts, trabeculae enhance wall deformability, buffer fluid-induced stress, and structurally reinforce the myocardium [15]. Finally, *in silico* drug trials have begun to emerge as a promising application, with whole-heart models offering mechanistic insight into drug-induced electromechanical dysfunction [16, 17].

**Figure 1:**
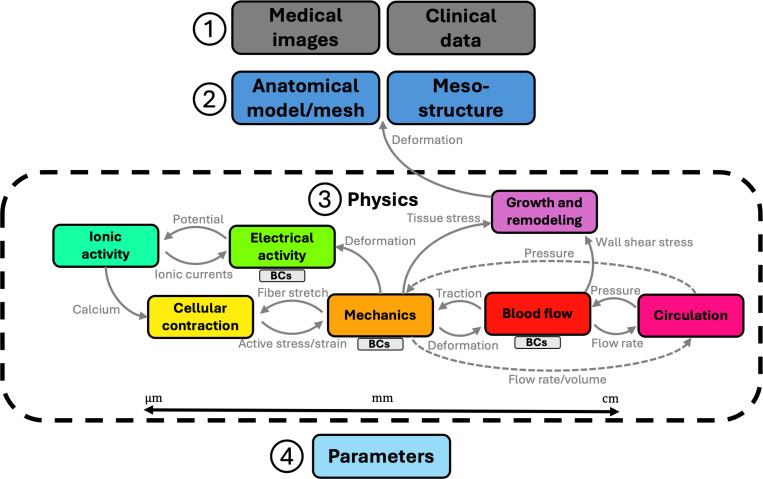
Summary of the major modeling considerations required when developing a personalized computational heart model. 1) The modeling process begins with medical images (CT or MRI), as well as other clinical data like an ECG or blood pressure measurements. 2) Next, a digital anatomic model of the heart is constructed by segmenting the medical image, then meshed in preparation for numerical simulation. The anisotropic mesostructure of the heart is also defined in this step. 3) Once the anatomical model and mesostructure are established, the relevant physics models must be selected and appropriately coupled, depending on the problem at hand. Accurate boundary conditions are essential. 4) Finally, the parameters of the computational model, which can number in the dozens, must be personalized to match patient data.

The essential stages in constructing a personalized computational heart model are illustrated in Figure 1. The process begins with a cardiovascular imaging exam using computed tomography (CT) or magnetic resonance imaging (MRI) to obtain comprehensive, often time-resolved anatomical images. Additional clinical data, such as electrocardiogram (ECG), blood pressure readings (either non-invasive cuff-based or invasive catheter-based), and ultrasound measures may also be collected (Figure 1 step 1). The CT or MRI images are segmented (in space and, if available, in time) to identify the myocardium, as well the four cardiac chambers – left atrium (LA), right atrium (RA), left ventricle (LV), and right ventricle (RV) – and other structures like the aorta and pulmonary arteries. Manual segmentation remains the gold standard, but machine learning has significantly accelerated this process [18, 19]. From the segmentation, typically a triangulated surface model is constructed, which is then volumetrically meshed using standard meshing software [20, 21] (Figure 1 step 2). A critical yet often overlooked decision involves determining the extent of the heart to include in the anatomical model; choices between left ventricular models, bi-ventricular models, and four chamber models, and around anatomical truncation and small-feature representation significantly impact both model fidelity and computational cost. The heart’s anisotropic mesostructure, commonly described as “myofibers” arranged in “sheetlets” [22], must also be accounted for, either directly from advanced cardiac MRI data [23] or, more commonly, using heuristic approaches known as rule-based methods [24]. For simulations involving cardiac electrophysiology, it is also necessary to define the conduction system, particularly the Purkinje network, which plays a critical role in coordinated electrical activation [25, 26, 27]. Similarly, when modeling intracardiac blood flow, realistic representations of the heart valves are required. These can be derived directly from medical imaging data [28], approximated using parametric geometries [29], or constructed from first principles based on mechanical and physiological considerations [30].

Once the anatomical model and mesostructure are established, the relevant physics for a particular problem must be selected (Figure 1 step 3). The heart is inherently a multiphysics system. During a cardiac cycle, electrical signals propagate through the conduction system, triggering ionic exchanges that cause cellular contraction. This generates stress in the myocardial tissue, leading to mechanical deformation, which in turn drives blood flow within the heart and throughout the circulatory system. We emphasize that appropriate boundary conditions (BCs) for electrophysiology [31, 32], tissue mechanics [33, 34], and blood flow [35] are critical for achieving physiological results. For some problems, it is also important to account for growth and remodeling (G&R), which describes the gradual changes in cardiac anatomy and structure driven by long-term mechanical stimuli [36]. Additional processes, such as perfusion of the myocardium [37] and valve dynamics [38], may also be modeled. The implementation of these physics in a numerical framework presents significant technical challenges, especially when coupling different physical processes, and some physics may be ignored depending on the problem being studied.

Finally, the parameters of the model should be “personalized” with the goal of matching model outputs to patient-specific clinical data (Figure 1 step 4). Depending on the complexity of the model, there could be tens or hundreds of model parameters, ranging from electrical conductivity to constitutive model coefficients to mesostructural orientations. This can be extremely challenging, and much work has focused on novel optimization methods [39, 40, 41], especially using neural network surrogates [42, 43, 44, 45], as well as sensitivity analyses [46, 47] and uncertainty quantification [42, 48, 49, 50].

In addition to the modeling considerations discussed above, a cardiac modeler must also select appropriate numerical solvers and configure their settings; these choices significantly affect both accuracy and computational efficiency and should not be overlooked. Focusing on tissue mechanics, the first decision is whether to use finite element analysis (FEA), which remains the standard, or newer approaches such as isogeometric analysis (IGA) [51] and meshless methods [52]. Within FEA, the element type (tetrahedral vs. hexahedral, linear vs. higher order) and the mesh resolution must be specified. For dynamic simulations, as opposed to static or quasistatic ones, one must also choose the time integration scheme (explicit vs. implicit) and time step size, which depend on the underlying physics and application. Additional considerations, including linear and nonlinear solvers, preconditioners, parallelization strategies, and multiphysics coupling schemes are also very important for efficient simulations. These issues are discussed in greater detail in Section 7. We also refer the reader to a recent cardiac mechanics benchmark for further guidance [53].

Several prior reviews have addressed different aspects of cardiac modeling. Avazmohammadi et al. (2019) [54] reviewed constitutive models for cardiac mechanics, while Niederer et al. (2019) [55] explored models of active tension generation from the cellular to organ scale. Bracamonte et al. (2022) [56] examined inverse modeling techniques for patient-specific simulations, and Rodero et al. (2023) [57] discussed the current state of multiphysics modeling, focusing on clinical translation. Arzani et al. (2022) [58] offered perspectives on the growing use of machine learning in physics-based models of cardiovascular biomechanics. This review will differ by focusing on recent developments and unresolved challenges, particularly in cardiac mechanics, that we believe are important to improving the physiological fidelity and clinical relevance of these models.

The subsequent sections explore the following topics. We begin with recent advances in patient-specific anatomical model construction, highlighting the use of machine learning to enhance efficiency (Section 2). This is followed by a review of myocardial mesostructure, including current strategies for its integration into computational models and the open questions that remain (Section 3). We then turn to active and passive constitutive modeling of myocardium, with a focus on viscoelasticity, compressibility, and myofiber dispersion (Section 4). Next, we examine commonly-used cardiac geometries, the interplay among different cardiac structures, and the importance of mechanical boundary conditions (Section 5). We then review the benefits and challenges of multiphysics modeling, with a focus on the interactions among mechanics, electrophysiology, hemodynamics, and circulatory dynamics (Section 6). Subsequently, we discuss important numerical challenges and considerations in cardiac multiphysics modeling (Section 7). Finally, we conclude by highlighting additional important areas of research, including machine learning techniques to accelerate simulation, growth and remodeling, and patient-specific modeling, and offer our perspective on future directions for the field (Section 8).

### Main takeaways

- **Anatomical model construction**: Creating accurate patient-specific anatomical heart models from medical images is labor-intensive when done manually. Machine learning methods appear essential for constructing models on clinically relevant timescales.
- **Myocardial mesostructure**: Myocardial mesostructure has a major impact on simulated mechanics, electrophysiology, and global function. Existing approaches oftentimes oversimplify the problem, which is complicated by inconsistencies in the literature. *In vivo* cardiac diffusion tensor MRI (cDTI) offers a patient-specific alternative, especially in pathological cases, though the technology still requires significant refinement.
- **Constitutive modeling**: Myocardial viscosity, compressibility, and fiber dispersion are well-supported in theory and modeling, but more work need to be done to establish their importance to cardiac function and modeling.
- **Anatomical model and boundary conditions**: Mechanical interactions among heart structures, as well as between the heart and its surroundings, influence simulation results. Boundary conditions are also a critical modeling component and, while they have been studied extensively, further research is needed for greater physiological accuracy.
- **Multiphysics coupling**: The heart is inherently a multiphysics and multiscale organ, and high-fidelity models should ideally capture these complexities. However, such models are difficult to build and tune, and simpler models are often adequate for specific clinical or research applications.
- **Numerical considerations**: Numerical solvers underpin the entire modeling pipeline, with the finite element method most commonly used. A rich body of work exists on improving simulation efficiency, including developments in discretization, preconditioners, and multiphysics coupling. Many cardiovascular-specific solver platforms are now available.
- **Future outlook**: Much of the computational foundation for cardiac digital twins is already in place, but demonstrating their clinical value remains the next major challenge, particularly with regard to validation and regulatory acceptance. Tackling this will expose technical and practical limitations in current models, while also clarifying which complexities are truly necessary for clinical impact and which can be simplified or accelerated with modern tools like machine learning.

## 2 Anatomical model construction

**Figure 2:**
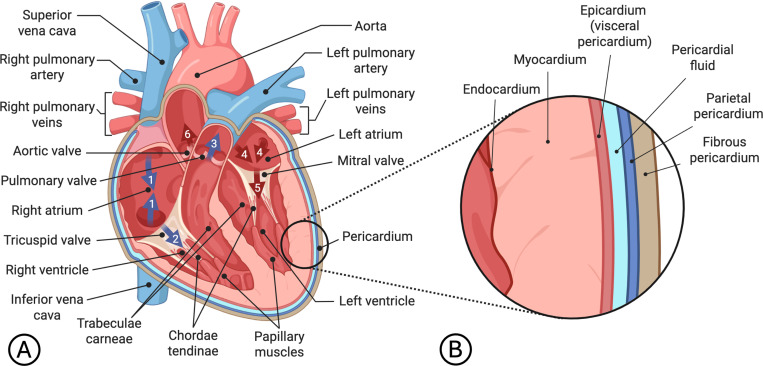
A) Major structures of the heart. B) Layers of the heart wall. Created in https://BioRender.com. Adapted from “The Heart: From the Organ to the Cell” template by Vinay Kara and Sally Kim on BioRender.

The first step in any cardiac mechanics simulation is the development of a geometric model of the domain of interest. Before exploring specific techniques for constructing these models, we review the basic anatomy of the heart, as a thorough anatomical understanding underpins accurate model generation. An illustration of the heart with an inset showing the layers of the heart wall is given in Figure 2.

Blood flow through the healthy heart follows a well-defined unidirectional sequence (Figure 2A), beginning with systemic venous return from the superior and inferior vena cava into the RA (step 1). From the RA, blood crosses the tricuspid valve (TV) (step 2), filling the RV with blood (diastole). During RV contraction (systole), the TV closes to prevent backflow, and the pulmonary valve (PV) opens, allowing deoxygenated blood to be pumped through the left and right pulmonary arteries into the lungs (step 3). After gas exchange occurs in the pulmonary capillary network, oxygenated blood returns via the pulmonary veins to the LA (step 4). Blood then flows across the mitral valve (MV) (step 5), filling the LV (diastole). During LV contraction (systole), the MV closes and the aortic valve (AV) opens, and oxygenated blood is pushed into the aorta and throughout the body (step 6).

Each of the four cardiac valves opens and closes passively in response to pressure gradients across the chambers and vessels. The atrioventricular valves (MV and TV), located between each atrium and ventricle, are anchored to the myocardium by chordae tendineae and papillary muscles, which prevent leaflet prolapse under high systolic pressure. The semilunar valves (PV and AV) control outflow from the ventricles into the pulmonary artery and aorta, respectively. Systemic arterial pressures (approximately 120/80 mmHg [59]) are markedly higher than pulmonary pressures (approximately 20/10 mmHg [60]), and the LV correspondingly has a much thicker myocardial wall than the RV [61]. The LA and RA also have relatively thin walls and they provide a supporting function to ventricular pumping, comprised of three distinct phases: reservoir, conduit, and booster pump [62]. An additional feature of the heart is the so-called trabeculae carneae — a network of spongy, muscle-lined ridges found on the inner surfaces of the ventricles.

The heart wall consists of several distinct layers (Figure 2B). The endocardium lines the interior surfaces of the heart chambers. Outside of this is the myocardium, a thick, muscular layer composed of a continuously branching syncytium of cardiomyocytes (contractile heart muscle cells) that perform the work of contraction. Surrounding the myocardium is the epicardium, which forms the outermost layer of heart tissue. The entire heart is enclosed in a stiff, fibrous pericardial sac. The epicardium (or visceral pericardium) is considered the inner layer of the pericardium. Between the epicardium and the parietal pericardium lies the pericardial cavity, which contains a thin layer of pericardial fluid. The outermost layer, or fibrous pericardium, is tethered to structures surrounding the heart, such as the lungs, diaphragm, sternum, ribs, aorta, and esophagus. As discussed in Section 5, the pericardium plays a significant role in constraining the motion of the heart [34].

Early computational heart models relied on simplified, idealized geometries, which offered a convenient means to study ventricular function. For example, simplified cylindrical or elliptical geometries were used to approximate the LV, while combined elliptical models for LV and RV have also been developed [63]. Other groups have made great progress using anatomically detailed, generic heart models. The Zygote heart [64], in particular, is commonly used [62, 65, 66]. Publicly available cohorts of heart models are also available [67]. One-dimensional models that capture parameters like sarcomere length have also been applied for studies where only limited geometric representation is needed [68]. Although such idealized, generic, or reduced order models are beneficial for parametric studies and benchmarking simulations [53], the morphology of the heart significantly influences its function (especially in pathological cases), and these models lack the anatomical precision needed for personalized heart simulations.

Personalized computational heart models should instead incorporate detailed anatomical features obtained from medical imaging. Typically, this process begins with acquiring three-dimensional computed tomography (CT) or magnetic resonance imaging (MRI) scans, followed by segmentation to identify the myocardium and other structures. The gold standard remains manual segmentation, which requires an expert to manually label anatomical structures within the 3D image stack. The segmentation is then “cleaned up” by removing features like trabeculae and papillary muscles, structures not usually included in the anatomical models due to their complexity. This process also involves smoothing to mitigate noise and artifacts introduced during segmentation. The resulting binary labeled image is then usually transformed into a surface mesh, followed by volumetric meshing to create a model suitable for finite element simulations [20, 21], although the segmentation can also be directly meshed [69]. Volumetric meshing of complex geometries is research field in and of itself, but in most cases meshing can be done using established software libraries such as TetGen [70], the Computational Geometry Algorithms Library (CGAL) [71], Gmsh [72], and Meshtool [73]. This standard pipeline is time-consuming and becomes increasingly challenging if multiple models must be generated from time-dependent data or for large patient cohorts [69].

Given these limitations, recent advances in machine learning have aimed to streamline the geometric model construction process. These automated methods accelerate segmentation, enabling heart mesh generation in seconds versus hours, with virtually no decrease in accuracy compared to manual segmentations [74]. In early work by [75], the authors developed an automatic four-chamber segmentation method using machine learning to first identify the position and orientation of the heart, then estimate the non-rigid deformation of control points to match the image. [76] used a deep convolutional neural network (CNN) approach to directly generate LV meshes from MRI images. To reduce the learning challenge, they first performed principal component analysis (PCA) on a large population of LV meshes to construct a low-dimensional feature space. Then, they trained two CNNs: the first learned to produce segmentations of the LV myocardium and blood pool from images, and the second learned to generate LV geometries (encoded in the PCA space) from the segmentations. [77] used CNNs and graph convolutional networks (GCNs) to automatically generate whole-heart meshes from medical images by learning to deform a small number of control points on a template heart surface mesh. To enhance mesh quality for computational fluid dynamics (CFD) simulations, they also introduced novel mesh regularization loss terms. In subsequent work, [78] refined the deformation process by instead learning a flow vector field that drives the mesh deformation. This modification ensures a diffeomorphic transformation and reduces the likelihood of mesh self-intersections. [74] developed a similar deformation-based deep learning method capable of generating high quality FEA-ready volumetric meshes, not only of myocardium but even valve leaflets. Most of these machine learning models have been trained on datasets of normal anatomies, and pathological cases can present additional complexities. For example, congenital heart diseases (CHDs) often involve substantial variations not only in heart morphology but also topology; for instance, a ventricular septal defect (VSD), where a hole in the interventricular septum connects the left and right ventricles, transforms two cavities into one. Recognizing these challenges, [79] recently developed specialized machine learning methods based on signed distance fields to produce simulation-ready meshes for patients with CHD.

To summarize, the heart’s complex anatomy presents significant challenges for computational modeling, motivating early studies to rely on idealized or generic geometries. Traditional workflows for patient-specific model generation, based on manual segmentation and meshing from medical images, are labor-intensive and time-consuming. However, recent advances in machine learning have rapidly transformed this landscape, enabling highly accurate, automated mesh generation. As interest in these techniques continues to grow, we should only expect further improvements in speed, accuracy, and applicability to increasingly complex anatomies and diverse patient populations.

We close this section by noting that, despite recent advances, *in vivo* imaging cannot directly capture the unloaded configuration of the heart or its residual stresses, both of which influence mechanical behavior [80]. Many studies simply assume an imaged configuration as the reference state, typically during diastole [81, 82, 83], when pressures are relatively low. This is a simplification, however, and several methods have been proposed to approximate the unloaded configuration [40, 84], often relying on iterative procedures. An alternative strategy is to compute a prestress field that balances the observed *in vivo* pressure [34, 85]. Yet even these approaches remain incomplete, as they typically neglect residual stresses, which are likely present and may play an important role in cardiac mechanics [34, 80].

## 3 Myocardial mesostructure

The myocardium, which constitutes the “functional tissue” [86] of the heart, is anisotropic owing to a continuously branching syncytium of cardiomyocytes embedded in a network of collagen and elastin [22]. Accurately accounting for the myocardial mesostructure is essential because it influences both cardiac deformation and the propagation of electrical signals [40, 87, 88, 89]. Myocardial mesostructure is extensively characterized in both animal models and humans [90], using techniques such as histology, confocal microscopy, cardiac diffusion tensor MRI (DTMRI or cDTI), and computed tomography (CT). These imaging methods primarily quantify the local orientation of cardiomyocytes, which defines the “grain” of the myocardium. Additionally, cardiomyocytes tend to form clusters often referred to as “sheets,” “sheetlets,” “lamellae,” or “myocardial aggregates” [22, 91, 92].

In cardiac mechanics simulations, myocardial mesostructure is typically modeled at the continuum scale using a local orthonormal coordinate system, with directions labeled as fiber (f), sheetlet (s), and sheetlet-normal (n) [93]. In this framework, f represents the local orientation of cardiomyocytes, n is a vector normal to the myocardial sheetlets, and s is a vector in the plane of the sheetlet orthogonal to both f and n in the undeformed or reference configuration. In a computational model, f determines the local direction of cardiomyocyte contraction (and if simulating electrophysiology, the preferred electrical propagation direction), while f, s, and n may all factor into the passive constitutive and active contraction models. The specifics of passive constitutive and active contraction modeling are discussed in later sections; here, we focus on mesostructure and its integration into computational mechanics models.

**Figure 3:**
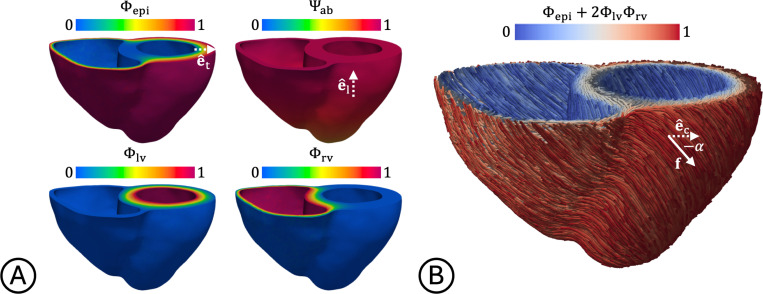
Using a Laplace-Dirichlet rule-based method (LDRBM) [96] to generate myocardial mesostructure on a truncated biventricular computational model. A) Visualization of the four Laplace fields required in this method. Each is generated by solving Laplace’s equation with Dirichlet boundary conditions on prescribed surfaces. ${\text{Φ}}_{\text{epi}}$ parameterizes the transmural depth, and its gradient defines the local transmural direction ${\hat{e}}_{\text{t}}$ (in the free walls). ${\text{Ψ}}_{\text{ab}}$ parameterizes the apex-to-base distance ^1^, and its gradient defines the local apicobasal or longitudinal direction ${\hat{e}}_{1}$. ${\text{Φ}}_{\text{lv}}$ and ${\text{Φ}}_{\text{rv}}$ are additional fields used in conjunction to parameterize the septum. B) Streamlines of the f field with a linear transmural variation from $\alpha =+60^{\circ }$ on the endocardium to $\alpha =-60^{\circ }$ on the epicardium, relative to the drawn local circumferential vector ${\hat{e}}_{\text{c}}$. The coloring by ${\text{Φ}}_{\text{epi}}+2{\text{Φ}}_{\text{lv}}{\text{Φ}}_{\text{rv}}$ is chosen to aid the eye in visualizing the streamlines.

Experimental studies have revealed characteristic patterns in the mesostructure of normal human myocardium [90]. In the ventricles, the fiber direction f varies through the wall thickness. Relative to the circumferential direction (which points counter-clockwise when viewed from the base of the heart), f shifts from a positive helix angle α on the endocardial surface (i.e., oriented toward the base) to a negative angle on the epicardial surface (i.e., oriented toward the apex). See Figure 3B. Standard ranges are, in the LV free wall, +6° to −60°, and in the RV free wall, +90° to −25° [24]; it should be noted, however, that these are nominal values and there are significant variations across studies, methods, and individuals. The transmural profile of f is often modeled as either linear or tangent-like [22, 94]. Recent observations suggest that the transmural variation is more complex in the interventricular septum, where two distinct fiber layers have been described [89]. In the atria, the myocardial mesostructure is also more complex, featuring multiple distinct and overlapping fiber bundles [95].

Based on these observations, “rule-based methods” (RBMs) have been developed to define myocardial mesostructure in arbitrary cardiac geometries without the need to incorporate generalized experimental data or patient-specific measurements. These methods define f, s, and n at any point in the domain based on empirical rules derived from normal myocardial mesostructural observations. Among the most widely used are Laplace-Dirichlet RBMs (LDRBMs), which employ numerical solutions to Laplace’s equation to parameterize the myocardium. We illustrate the approach by applying the LDRBM of Bayer et al. [96] on an example truncated biventricular geometry (Figure 3). First, a local anatomic coordinate system is established at every point in the heart, consisting of radial or transmural $\left({\hat{e}}_{\text{t}}\right)$, apex-to-base or longitudinal $\left({\hat{e}}_{\text{l}}\right)$, and circumferential $\left({\hat{e}}_{\text{c}}\right)$ basis vectors. The transmural direction is obtained by solving a Laplace problem with a Dirichlet boundary condition of zero on the endocardial surface and one on the epicardial surface.


$$\begin{array}{l} \nabla^{2}{\text{Φ}}_{\text{epi}}=0,\\ {\text{Φ}}_{\text{epi}}=0\;\text{on}\;{\text{Γ}}_{\text{endo}},\\ {\text{Φ}}_{\text{epi}}=1\;\text{on}\;{\text{Γ}}_{\text{epi}}. \end{array}$$

The resulting “temperature” field ${\text{Φ}}_{\text{epi}}$ (Figure 3A) serves as a parameterization of transmural depth (in the free walls), with its gradient defining the transmural direction ${\hat{e}}_{\text{t}}$. Likewise, ${\hat{e}}_{\text{l}}$ is defined as the gradient of an apicobasal field, ${\text{Ψ}}_{\text{ab}}$, obtained by solving a Laplace problem with zero at the apex and one at the base. The circumferential direction ${\hat{e}}_{\text{c}}$ is then defined as the vector orthogonal to both ${\hat{e}}_{\text{t}}$ and ${\hat{e}}_{\text{l}}$. Subsequently, a local mesostructural coordinate system {f,s,n} is obtained by prescribed rotations relative to the anatomic coordinate system. f, for example, is obtained by rotating ${\hat{e}}_{\text{c}}$ by an angle α around ${\hat{e}}_{\text{t}}$. Note that {f,s,n} is not properly defined in the septum because ${\text{Φ}}_{\text{epi}}$ does not appropriately parameterize the septum. In the Bayer method, this is resolved by generating additional Laplace fields to distinguish the LV $\left({\text{Φ}}_{\text{lv}}\right)$ from the RV $\left({\text{Φ}}_{\text{rv}}\right)$, which are used to define a second mesostructural coordinate system that is accurate in the septum. The two systems are then blended using bidirectional spherical linear interpolation, which ensures smooth transitions in fiber orientations across the LV, RV, and septal junctions. Full methodological details are provided in Bayer et al. [96].

Initially based on observations of the LV mesostructure and proposed for a biventricular model truncated in the basal plane (below the valve annuli) [96], subsequent LDRBMs have been adapted to better represent the mesostructure in other regions of the heart, such as the ventricular septum, outflow tracts, right ventricle, and atria [24, 98], and to model the transmural variation of f more accurately [99]. Several LDRBMs were considered under a uniform framework and compared in electrophysiology simulations [24] and electromechanical simulations [88].

However, recent discussions have highlighted ambiguities regarding myocardial mesostructure that may impact the accuracy of cardiac mechanics simulations. The review by [92] underscores a lack of consensus on methodologies for quantifying cardiomyocyte orientations, including inconsistencies around reporting either helical angles or their projected angles, which measure the angle between the circumferential direction and the *projection* of f onto the plane tangent to the epicardial surface. The authors recommend reporting the helical angle without projection. They also suggest angles be measured relative to epicardial curvature rather than the heart’s long axis, and that myocardial sheetlet orientations be assessed using the normal vector n. Additionally, although computational studies often assume that f lies within the wall plane, physiological evidence suggests this is not always accurate and the transmural component (i.e. imbrication angle) of f should be considered [22, 100]. Finally, experimental work suggests that cardiomyocytes may exhibit large-scale connectivity and assume a toroidal topology [101], a feature that is not currently considered in RBMs and may be especially important at the base and apex of the ventricles (top and bottom of the torus).

Another issue involves histological and DTMRI studies that have observed two populations of sheetlet directions at a given myocardial location [102], challenging the common assumption of a single s direction in myocardial constitutive models. The implications of this structure, described as forming a “herringbone” pattern [22], on constitutive and cardiac mechanics modeling needs further investigation. Related to these issues is an apparent error in the definition of the sheetlet angle β in the well-known Bayer LDRBM [96], which appears to have propagated through the cardiac modeling literature. While the figure in [96] suggests that β is associated with a counterclockwise rotation about f, the provided equation implements a clockwise rotation.

Due to these issues, we believe that care should be taken when applying mesostructural values from literature to ensure that the data and computational model are consistent. Collaborative efforts bridging experimental and computational researchers could help address these issues.

Given the importance of mesostructure in cardiac electromechanics, the ideal cardiac digital twin should incorporate patient-specific mesostructure, which is not realistically achievable with LDRBMs limited by only a few tunable angle parameters. Computational studies have identified significantly different results when comparing the smooth and spatially homogeneous LDRBM-generated mesostructures versus experimentally observed mesostructure [87, 103]. Moreover, current LDRBMs are based primarily on data from healthy cases, though patient-specific models would be most valuable for diseased hearts, where mesostructure often differs markedly due to congenital or acquired heart diseases and remodeling processes [22, 104, 105, 106, 107, 108]. To provide some degree of personalization, Washi et al. [109, 110] proposed a novel fiber optimization algorithm, in which fibers are locally reoriented based on the predominant direction of active tension generation during isovolumic contraction, but this remains to be validated. To achieve truly patient-specific myocardial mesostructures, *in vivo* cardiac diffusion tensor MRI (DTMRI or cDTI) is under active investigation [22, 111, 112, 113, 114], but faces several challenges, such as long acquisition times, limited spatial resolution, and the challenges associated with compensating for bulk cardiac motion [57, 115]. At the same time, the emergence of 3D histological methods and cDTI is shifting the field away from simplified angular descriptions of mesostructural features toward vector- and tensor-field representations, which can be mapped directly onto computational meshes [103, 115].

## 4 Constitutive modeling

Experimental mechanical testing on animal and human samples has revealed that the myocardium is a non-linear, anisotropic, viscoelastic, history-dependent material [94]. Early work assumed transverse isotropy, but the modern consensus is for orthotropy [116]. Most material testing has focused on the ventricles, in particular the LV, although recent work has identified regional differences in myocardial properties [117, 118]. Existing constitutive models generally contain an isotropic term accounting for the non-fibrous underlying matrix [86], with additional tensile stiffness along f and sometimes s or n, as well as shear stiffness arising from interactions between f, s, and n [54]. In the following, we briefly describe some prominent constitutive models for the myocardium.

We first discuss the transversely isotropic Guccione model [119, 120]:

(1) $$\psi_{G}=\frac{C}{2}\left(e^{Q}-1\right),$$

where

(2) $$Q=2b_{1}\left(E_{ff}^{2}+E_{ss}^{2}+E_{nn}^{2}\right)+b_{2}E_{ff}^{2}+b_{3}\left(E_{ss}^{2}+E_{nn}^{2}+E_{ns}^{2}+E_{sn}^{2}\right)+b_{4}\left(E_{fs}^{2}+E_{sf}^{2}+E_{fn}^{2}+E_{nf}^{2}\right),$$

and

(3) $$E_{ab}=a\cdot \mathrm{Eb};\;a,b\in \left\{f_{0},s_{0},n_{0}\right\},$$

where $f_{0}$, $s_{0}$, $n_{0}$ are the fiber, sheetlet, and sheetlet-normal directions in the reference configuration. The material parameters are C, which scales the stiffness, and $b_{1}$, $b_{2}$, $b_{3}$ and $b_{4}$, which control the degree of strain stiffening in tension, compression, and shear. This has been used recently in [41, 121, 122].

The Usyk model [123, 124] is an orthotropic variation of the Guccione model:

(4) $$\psi_{U}=\frac{C}{2}\left(e^{Q}-1\right),$$

where

(5) $$Q=b_{ff}E_{ff}^{2}+b_{ss}E_{ss}^{2}+b_{nn}E_{nn}^{2}+b_{fs}\left(E_{fs}^{2}+E_{sf}^{2}\right)+b_{fn}\left(E_{fn}^{2}+E_{nf}^{2}\right)+b_{ns}\left(E_{ns}^{2}+E_{sn}^{2}\right).$$

$E_{ab}$ is defined as in the Guccione model, and C and $b_{ij}$, i, j∈{f,s,n} are material parameters with similar interpretations as in the Guccione model. This has been used recently in [62, 83, 84].

Finally, the orthotropic, invariant-based Holzapfel-Ogden (HO) model [86] has been widely adopted and takes the form^2^

(6) $$\psi_{HO}=\frac{a}{2b}\left(e^{b\left(I_{1}-3\right)}-1\right)+\frac{a_{f_{s}}}{2b_{f_{s}}}\left(e^{b_{fs}I_{8,fs}^{2}}-1\right)+\sum_{i\in \{f,s\}}\chi \left(I_{4,i}\right)\frac{a_{i}}{2b_{i}}\left(e^{b_{i}{\left(I_{4,i}-1\right)}^{2}}-1\right),$$

where the a parameters scale the stiffness, and the b parameters control the degree of strain stiffening. χ(x) is a (potentially smoothed) Heaviside function centered at x=1, which enforces the assumption that fibers support stress in tension only [40]. This has been used recently in [53, 103, 125].

In the literature, there are various modifications to these models, such as using isochoric strain or strain invariants and adding a volumetric energy term in a nearly incompressible formulation [53, 126, 127]. Another example is the work of [128], who showed that when modeling compressible materials, the use of full (rather than isochoric) anisotropic invariants in an anisotropic material model like the HO model is necessary to properly capture anisotropic volumetric deformations [40, 129]. We also mention the work of [130], who modified the sheetlet term in the HO model with an invariant that measures the change in area of a myocardial sheetlet, demonstrating significant improvement in the quality of the fit to experimental tension and shear data.

For a review of myocardial constitutive models, see [54]. Here, we focus on three developments in constitutive modeling that have not been fully integrated into standard practice: viscosity, compressibility, and fiber dispersion.

### 4.1 Viscosity

The models discussed above were primarily developed under the assumption of an elastic myocardium, with no consideration for viscoelastic behavior. Although increased myocardial viscosity has been associated with heart disease [131], it was believed by many to be “not important on the time scale of the cardiac cycle” [86]. Additionally, the modeling of viscoelasticity has been hindered by a lack of comprehensive experimental data [86]. Moreover, the mechanisms behind myocardial viscoelasticity remain debated, with various theories suggesting contributions from viscous effects related to blood or extracellular fluid flow through the porous myocardium, intrinsic viscoelasticity of cardiomyocytes, and molecular friction among extracellular matrix constituents, particularly collagen [132, 133]. However, there has been a recent resurgence of interest in the viscoelastic properties of the myocardium and their potential relevance to cardiac function in both healthy and diseased states [131].

In particular, [94] compiled a dataset from planar biaxial extension and triaxial simple shear experiments on excised human ventricular myocardium, using strain rate modulation and stress relaxation tests to quantify viscoelastic behavior. Their findings revealed significant hysteresis even under quasistatic loading, indicative of substantial energy dissipation within the tissue. This data was used to develop and calibrate several viscoelastic models of the myocardium, constructed as extensions of existing hyperelastic models; these are discussed in the following paragraphs. However, given the *ex vivo* nature of these experiments and the state of the tissue, it remains unclear how important these effects are for *in vivo* modeling efforts.

[127] proposed a viscoelastic model for the myocardium based on a three-dimensional, nonlinear analogue of the generalized Maxwell model. This model combines multiple Maxwell elements (spring and dashpot connected in series) in parallel with hyperelastic components, using four Maxwell elements to account for viscoelasticity in the isotropic ground matrix (m), as well as fiber (f), sheetlet (s), and fiber-sheetlet (fs) components. The viscous overstress for each component α is given by the following convolution integral expression:

(7) $$S_{visc,\alpha }=\int_{0}^{t}\exp\left(-\frac{t-s}{\tau_{\alpha }}\right)\beta_{\alpha }{\dot{S}}_{iso,\alpha }^{\infty };\;\alpha \in \{m,f,s,fs\},$$

where $\beta_{\alpha }$ are four scaling factors for the viscous strength, $\tau_{\alpha }$ are the corresponding four viscous relaxation times, and ${\dot{S}}_{iso,\alpha }^{\infty }$ are the rates of change of the corresponding hyperelastic contribution to the stress.

These parameters were calibrated to experimental data from [94]. They also provided details on an efficient implementation of their model within a FEA framework. In test cases, the model generally captured biaxial extension data well, but it could not fully replicate the hysteresis shape in shear experiments and showed discrepancies in stress relaxation experiments, particularly at early times. In simulations of an idealized LV subjected to time-varying pressure loading, the model predicted transmurally varying viscoelastic responses, with significantly more pronounced hysteresis on the endocardial surface.

A simplified version of the generalized Maxwell model, using a single Maxwell element to represent the viscoelasticity of the isotropic ground matrix, was used in a four-chamber heart model by [134]. Their parameter sensitivity study concluded that “viscous relaxation has a negligible effect on the overall behavior of the heart,” as assessed by left ventricular pressure-volume loops and myocardial fiber strain. Essentially, while changes in the viscous parameters significantly altered the mechanical response, the effect was primarily to increase stiffness, so that a stiffer hyperelastic material was able yield similar results. However, they observed that viscosity led to a cycle-to-cycle shift in pressure-volume loops, suggesting that “relaxation is significant between cardiac cycles, even though it is not significant within an individual cycle.” This was attributed to the relatively long viscous relaxation time (on the order of O(10) seconds) compared to the short duration of a single cardiac cycle (<O(1) second).

Nordsletten et al. [133] employed a fractional viscoelastic approach, implementing a fractional Zener model [135], which describes the viscoelastic stress via a fractional differential equation:

(8) $$S_{ve}+\delta D_{t}^{\alpha }S_{ve}=D_{t}^{\alpha }S_{v},$$

where $D_{t}^{\alpha }$ is the Caputo derivative, defined as

(9) $$D_{t}^{\alpha }f=\frac{1}{\text{Γ}(1-\alpha )}\int_{0}^{t}{\left(t-s\right)}^{-\alpha }\dot{f}(s)ds,$$

with α∈[0,1] representing the order of the derivative. In this formulation, $S_{v}$ is given by an expression that mimics the elastic stress of the Holzapfel-Ogden (HO) model. While similar in form to the convolution integral in Eq. (7), whereas Eq. (7) models only a few discrete relaxation timescales for viscosity, Eq. (8) encodes a continuous distribution of relaxation timescales, which better reflects the multiscale nature of myocardial viscoelasticity. The distribution’s shape is controlled by the parameters α and δ. The new model demonstrated superior fitting to experimental data compared to both traditional hyperelastic models and the Maxwell-based approach of [127]. Furthermore, the identifiability of the 11 model parameters was successfully demonstrated.

[136] then incorporated this fractional viscoelastic model into a finite element framework, addressing several numerical challenges associated with solving the fractional differential equation [137]. Their model was applied to an idealized LV coupled with a 0D circulation model. Comparisons with a hyperelastic model (Holzapfel-Ogden) revealed that the viscoelastic model dampened spurious oscillations and reduced stroke work, along with other small but noticeable differences in LV deformation, fiber stress, and myocardial pressure. They argued that viscoelasticity plays its most important role during the relaxation phase of the cardiac cycle.

In addition to these advanced viscoelastic models, simplified approaches that approximate some viscous effects have also been applied in the literature. These include a viscous pseudopotential model [34, 53, 138]

(10) $$S_{visc}=\frac{\partial }{\partial \dot{E}}\psi_{visc},\;\psi_{visc}=\frac{\mu }{2}\mathrm{tr}\left({\dot{E}}^{2}\right),$$

a Newtonian viscous model [139]

(11) $$S_{visc}=\mu JF^{-1}\left(\nabla v+\nabla v^{T}\right)F^{-T},$$

and Rayleigh damping [121, 66], which adds a damping term

(12) C(u)=αM+βK(u)

to the time-space discretized elastodynamics equation $M\overset{..}{u}+C(u)\dot{u}+K(u)=R$ [140].

In summary, while significant progress has been made in developing and applying viscoelastic models for the myocardium, the importance of viscous effects at the relevant timescales remains under debate and warrants further investigation.

### 4.2 Compressibility

Most constitutive models and the majority of cardiac mechanics studies assume that myocardium, like other biological tissues with high water content, is “essentially incompressible” [86, 119, 141, 142]. Early experimental studies have been cited to justify this assumption of myocardial incompressibility [143, 144]. However, it is worth noting that [144] is not readily available through internet searches, and we were unable to locate the original publication. [141] tested the hypothesis that myocardial perfusion through the coronary circulation changes throughout the cardiac cycle due to variations in myocardial stiffness, leading to bulk compressible behavior. In their cyclic biaxial experiments on perfused, *ex vivo* ventricular septa from dogs, they observed that the volume of myocardial tissue occupied by vasculature changed by 2–4% depending on perfusion pressure. Using noninvasive MRI, [145] measured myocardial volume changes below 2%. Based on the evidence at the time, [146] concluded in 2008 that “the total myocardial volume changes no more than 4% during a cardiac cycle,” supporting the assumption of near incompressibility.

However, recent investigations have challenged this assumption. In an *in vivo* study of canine hearts, [147] quantified myocardial volume changes across different layers, observing a 4.1% systolic reduction in the sub-epicardial layer, 6.8% in the midwall, and 10.3% in the sub-endocardial layer. They further hypothesized the “existence of blood-filled spaces within the myocardium” that could directly exchange blood with the ventricular lumen, as the observed volume change exceeded what could be accounted for by coronary blood flow. Interestingly, they also noted that volume reductions in early activated myocardial regions occurred before the rise in chamber pressure, suggesting that these changes were due to myofiber contraction rather than compression caused by elevated chamber pressure.

In another study, [129] conducted *ex vivo* tension and compression experiments on cylindrical specimens of porcine myocardium. They found a 4% volume change under uniaxial tension at a stretch of 1.3. In confined compression, they observed a highly nonlinear reponse, with a stress of 4 kPa at 5% volume strain, rising to nearly 50 kPa at 10% strain. To model these experimental results, they replaced the isotropic term in the Holzapfel-Ogden (HO) model (Eq. (6)) with a six-parameter Yeoh model, which accurately captured the myocardium’s slightly compressible behavior. They also developed a representative volume element (RVE) consisting of cardiomyocytes, extracellular matrix material, and cylindrical voids representing capillaries. Simulation of this RVE under confined compression revealed that only 42% of the total volume change could be attributed to capillary volume reduction, suggesting that the compressibility of the solid tissue itself must contribute significantly to myocardial volume change.

[148, 149] used sonomicrometry to measure left ventricular free wall deformation in an ovine heart. In healthy animals, they observed significant myocardial compressibility, with important regional and transmural variations. On average, they recorded a 15% systolic volume reduction, with up to 25% reduction at the endocardial surface, and slightly greater compressibility at the apex. After myocardial infarction, which eliminates blood perfusion and tissue contraction, the infarcted region showed much smaller systolic volume reductions, and in some cases, the infarcted tissue even increased in volume during systole. In a follow-up study, [150] compared compressible and incompressible models in organ-level cardiac mechanics simulations. Based on their experimental findings, they characterized the myocardium as nearly incompressible during diastole but compressible during systole. To account for this, they proposed a material model in which the volumetric penalty parameter varies as a negative linear function of the active stress magnitude, allowing for compressibility during systole when active stress is maximized [148]. Their simulations demonstrated that incorporating compressibility improved the agreement with experimental data, particularly showing that an incompressible model led to exaggerated systolic wall thickening, reduced LV longitudinal shortening, and a much higher active stress required to match the experimental pressure-volume loop.

Myocardial compressibility was also shown to be reduced in patients with heart failure with reduced ejection fraction (HFrEF). [151] used cardiac MRI and transthoracic echocardiography (TTE) to measure myocardial volume changes. They found that in healthy individuals, systolic myocardial volume was 87% or 79% of the diastolic volume, based on MRI and TTE measurements, respectively. In contrast, MRI data from HFrEF patients indicated that systolic myocardial volume equaled diastolic volume, suggesting that while healthy myocardium is compressible, myocardium in HFrEF patients is incompressible.

In conclusion, existing evidence suggests that the myocardium is compressible *in vivo*, although the degree of compressibility and underlying mechanisms remain incompletely understood. Additionally, myocardial compressibility appears to be altered in diseased states, but this is also of uncertain functional consequence. More research is needed to fully elucidate the role of compressibility and determine the best way to model it in simulations.

We close by noting that it is generally easier to simulate compressible materials than incompressible ones, as incompressibility often requires special numerical treatments to avoid ill-conditioned matrices (Section 7).

### 4.3 Fiber Dispersion

While myocardial mesostructure is often modeled as a continuously varying fiber field, myofibers f (and sheetlet orientations s) exhibit significant dispersion at the mesoscale. Within an appropriately sized region of interest (ROI), these fibers are not perfectly aligned with the mean direction, but instead have orientations dispersed around it. For instance, [152] analyzed 2D histological sections of rat septum and quantified dispersion as the standard deviation of fiber angle within a small tissue subregion (approximately 0.01 mm^2^). Other studies estimated dispersion by fitting an analytic distribution, characterized by a dispersion parameter, to the histogram of fiber angles within a dataset of a given size [94, 153, 154, 155]. Notably, the ROI size is important [156]; [153] investigated the effect of ROI size on fiber distribution estimates and provided guidelines for finite element analysts to select mesh sizes that balance capturing spatial inhomogeneities with statistical robustness and computational feasibility. Dispersion can also be assessed using cDTI, where low fractional anisotropy correlates with high dispersion [22]. Further work with cDTI could better characterize dispersion throughout the heart using more advanced acquisition methods and signal models.

Fiber dispersion varies significantly between individuals [94]. Moreover, enhanced fiber dispersion, or “disarray”, is a hallmark of certain cardiac diseases [104, 105, 107] and can lead to impaired mechanical function [152]. Simulations of cardiac mechanics have further demonstrated the importance of fiber dispersion [103, 126].

From a constitutive modeling perspective, fiber dispersion was first incorporated into myocardial models by [126]. They extended the standard myocardial HO model (Eq. (6)) [86] using a generalized structure tensor (GST) approach, originally developed to model collagen dispersion in arterial tissue [157]. This approach has been applied in recent studies [158, 134]. Briefly, the orientation probability density function at an angle Θ from the mean fiber or sheetlet direction was described by a normalized π-periodic von Mises distribution:

(13) $${\text{ρ}}_{i}(\text{Θ})=4\sqrt{\frac{b_{\text{ρ}}}{2\pi }}\frac{\exp\left[b_{\text{ρ}}(\cos(2\text{Θ})+1)\right]}{(-i)\text{erf}\left(\sqrt{2b_{\text{ρ}}}\right)},\;i\in f,s,$$

where $b_{\text{ρ}}$ is a concentration parameter, and erf(x) is the error function. This rotationally symmetric distribution was fit to limited data in the literature at the time. Dispersion parameters $\kappa_{f}$ and $\kappa_{s}$ for fibers and sheetlets were computed as

(14) $$\kappa_{i}=\frac{1}{4}\int_{0}^{\pi }{\text{ρ}}_{i}(\Theta )\sin^{3}\Theta d\Theta ,$$

and the standard HO model was modified to account for dispersion

(15) $$\psi_{HO}=\frac{a}{2b}\left(e^{b\left(I_{1}-3\right)}-1\right)+\frac{a_{fs}}{2b_{fs}}\left(e^{b_{fs}I_{8,fs}^{2}}-1\right)+\sum_{i\in \{f,s\}}\chi \left(I_{4,i}^{*}\right)\frac{a_{i}}{2b_{i}}\left(e^{b_{i}{\left(I_{4,i}^{*}-1\right)}^{2}}-1\right).$$

where the dispersion-modified fiber and sheetlet invariants $I_{4,i}^{*}$ are defined as

(16) $$I_{4,i}^{*}=\kappa_{i}I_{1}+\left(1-3\kappa_{i}\right)I_{4,i},\;i\in f,s.$$

Later, [159] incorporated dispersion into the fs term as well. In addition, a non-symmetric, two-parameter dispersion model has also been developed, first for collagen fibers in arterial walls [160], and later adapted for myocardium [161]. This model assumes the probability density is the product of independent functions describing in-plane and out-of-plane dispersions.

The GST approach should be compared to the alternative angular integration (AI) or continuous fiber dispersion method, in which the contribution to the total strain energy of a single fiber at a given stretch is integrated over all directions or solid angles, weighted by the probability density [160]. The AI and GST approaches have been directly compared in [162, 163, 164], with particular attention paid to whether GST properly excludes fibers under compression and the computational cost of AI. To address these issues, [165] modified the GST approach with a general invariant that properly excludes compressed fibers. Later, the same authors applied a discrete fiber dispersion (DFD) approach based on the AI approach, and showed similar results to AI with a significant reduction in computational cost [166].

Finally, while the role of dispersion in the passive myocardial response has received much attention, less is known about the effect of dispersion on myocardial contraction. Fiber dispersion appears to be at least partly responsible for significant cross-fiber stresses generated during contraction [167, 168], which is likely important to physiological deformation [100, 103, 169]. This has been modeled in several studies by adding a cross-fiber active stress, which can depend on the degree of dispersion [123, 126, 170].

In summary, there has been significant work in characterizing and modeling fiber dispersion, but its importance in cardiac mechanics, especially in diseased cases and with respect to active contraction, should be investigated further.

## 5 Importance of anatomic model and mechanical boundary conditions

**Figure 4:**
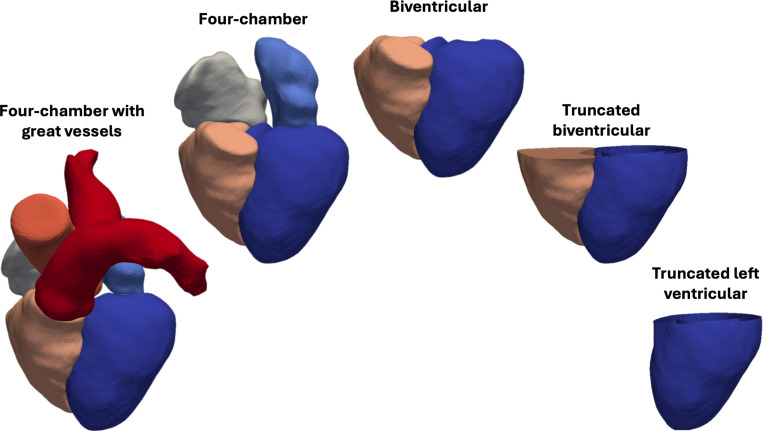
Representative cardiac geometries commonly used in the literature. Four-chamber models, sometimes including the great vessels, offer the most comprehensive anatomical representation but are difficult to reconstruct from medical imaging and substantially increase computational complexity. In contrast, biventricular (BiV) and LV models, sometimes truncated at the basal plane have been more widely used due to their reduced complexity, although the physiological consequences of excluding other cardiac structures remain poorly understood.

A variety of anatomical models have been used in the cardiac modeling literature (Figure 4). Whole-heart models, which include all four chambers and sometimes the trunks of the aorta and pulmonary arteries, are the most realistic [34, 41, 62, 171, 172, 173, 174], but constructing patient-specific models remains challenging due to limited medical image resolution in the thin-walled RV, atria, and basal portions of the ventricles [175]. Additionally, whole-heart simulations tend to be more complex to develop and calibrate [33]. Because of these challenges, biventricular (BiV) models that include only the LV and RV are common. Many BiV models are further simplified by truncating the geometry at the “basal plane” [33, 85]. An even further simplification is to include only the LV, which may also be truncated at the basal plane [175]. These simplifications arise, in part, from the complexity of building models with multi-chamber geometries from coarsely sampled medical imaging data. They also stem from the expectation that the ventricles, particularly the LV, are the primary force generators underlying cardiac function. However, “even subtle changes in cardiac anatomy can have a large impact on cardiac function” [176], so it follows that the choice of anatomic model may significantly influence the outcomes of cardiac mechanics simulations. This raises important questions, for example: How does the RV influence LV function, and vice versa? Through what mechanisms do the atria and ventricles interact, and how significant are these interactions?

Closely tied to the choice of anatomic model is the selection of boundary conditions (BCs), which are equally important in determining cardiac function [177]. For example, when using a truncated BiV or LV model, the modeler must decide on an appropriate mechanical boundary condition for the basal surface. Ideally, this boundary condition should replicate the mechanical influence of the basal ventricular tissue and atria, to the degree that such an influence exists.

In this section, we examine the mechanical interactions among various cardiac structures, highlighting how the omission of certain structures may impact simulation results. Additionally, we review commonly used boundary conditions designed to approximate, with varying degrees of fidelity, the effects of structures excluded from the computational domain.

### 5.1 Mechanical interactions among structures of the heart

The mechanical interaction between LV and RV, known in the literature as ventricular interdependence [178], occurs through direct and indirect mechanisms. The ventricles share a common septum, which provides direct coupling, while additional interactions arise via blood flow through the systemic and pulmonary circulations. Indeed, at steady-state operation without valve regurgitation or intracardiac shunting, LV and RV stroke volumes must be similar, or else blood would accumulate somewhere in the body [179]. Additionally, important interactions exist through the pericardium [180, 181, 182, 183]. Both directions, LV to RV and RV to LV, have been shown to be important, especially in diseased cases [184, 185].

Early work by [186] introduced a reduced-order mathematical model to study ventricular interdependence, modeling the LV free wall, RV free wall, septum, and pericardium with separate time-varying elastances. Their findings, consistent with experimental observations, include the following. Increased diastolic pressure in one ventricle reduces the compliance of the other, and the pericardium enhances the mechanical coupling between ventricles. Additionally, when the septum does not contract, it bulges into the RV during systole, creating a “parasitic” effect – RV pumping is enhanced at the expense of LV function. This effect is eliminated with a contracting septal wall. In recent work, [180] used a one-dimensional BiV model with pericardium and closed-loop circulatory model to investigate the effects of LV and RV systolic and diastolic dysfunction. Their results showed that LV dysfunction prompts the RV to compensate by increasing cardiac power output, whereas RV dysfunction reduces LV filling, a phenomenon that could be misinterpreted clinically as LV diastolic dysfunction (heart failure with preserved ejection fraction). Similarly, [187] developed a patient-specific finite element model to investigate the nature of ventricular interdependence in the presence of a left ventricular assistance device (LVAD). They found that greater LVAD support reduces LV pressure and myofiber stress, shifts the RV PV loop to higher pressures and volumes, and causes abnormal bending of the septum into the LV.

Atrioventricular interactions occur through direct tissue coupling at the atrioventricular plane, as well as via blood flow exchanges across the mitral and tricuspid valves [188]. As with ventricular interdependence, the pericardium may provide an additional interaction mechanism. The atria have conventionally been understood to support ventricular function in three distinct roles: serving as a reservoir of blood during ventricular systole, acting as a conduit in early diastole, and functioning as a booster pump in late ventricular diastole [62, 189]. However, the ventricles, as the larger and stronger chambers, play an equally important role in atrial function. [172], for example, used a four-chamber model to investigate the interactions among the ventricles, atria, and pericardium. They found that ventricular contraction is important for atrial filling, creating a suction of blood from the venae cavae and pulmonary veins into the atria. Similarly, [190] found that increasing ventricular fiber angles from +40°/ −40° to +60°/ −60° increased atrioventricular plane motion from 1.0 mm to 14.0 mm and increased LA maximum volume by 8 %.

The atria influence ventricular function primarily through their role as booster pumps, though there is some conflicting evidence about the size of their impact. [62] compared whole-heart simulations with and without atrial contraction and found that without atrial contraction, ventricular end-diastolic volumes and peak systolic pressures decreased significantly. In contrast, [191] found that while variations in atrial calcium transient models strongly affected atrial function, their impact on ventricular PV curves was relatively small. [192] simulated LA ablation in a four-chamber model, assessing how altered electrical activation and increased stiffness from ablation scars affect cardiac function. With ablation, LV stroke volume decreased by only 1.8 %, though they suggest the effect may be larger in older populations. Increased LA stiffness also reduced atrioventricular plane displacement, leading to lower peak systolic pressure in the LV, though the effect was modest. Notably, the RA and RV were essentially unaffected by LA ablation. Even an entirely passive atria may still influence ventricular mechanics. [66] compared a biventricular model with a whole-heart model and found that even non-contracting atria significantly affected ventricular deformation, specifically atrioventricular plane displacement and radial displacement of the basal portions of the LV and RV free walls. They attributed these effects to the added inertia of atrial tissue.

The existing evidence clearly demonstrates the significance of mechanical coupling among cardiac chambers, though the precise mechanisms and clinical importance of these interactions warrant further study. These considerations are particularly relevant for computational heart modelers, who must balance anatomical complexity with computational efficiency. In addition to the four chambers, it is important to consider the roles other cardiac structures, including the roots of the great vessels [81, 171, 191], the trabeculae and papillary muscles [193, 194, 195], and epicardial adipose tissue [34, 171, 196].

### 5.2 Mechanical boundary conditions

Arguably the most important BC in cardiac mechanics simulations is the one applied to the endocardial surface of the heart chambers, representing the traction exerted by the blood on the heart wall. This traction is commonly modeled as a spatially-uniform pressure, either prescribed directly [53] or determined through a coupling with a 0D circulation model [197]. Alternatively, it can be explicitly computed in a fluid-structure interaction (FSI) simulation [198, 199]. While this class of BC is relatively straightforward, the appropriate formulations of other BCs can be more ambiguous. Here, we discuss two important examples: BCs on the basal surface in truncated BiV and LV models and BCs on the epicardial surface to capture the effect of the pericardium, relevant to all anatomical models.

**Basal BCs** A variety of basal BCs have been proposed in the literature. It is important to recognize that *in vivo* the basal aspects of the ventricles plunge towards the apex during contraction, while the apex remains relatively fixed in position. The apex does, however, rotate counter-clockwise relative to the base, which is largely held in place at the level of the atria by the blood vessels. Early approaches constrained the basal plane in the long-axis direction, while handling in-plane motion in different ways. [33] compared five such basal BCs on truncated biventricular geometries, finding notable differences in global metrics and statistically significant, though minor and localized variations in tissue strain. They advised against fixing individual basal nodes, recommending instead BCs that constrain only the average in-plane motion of basal nodes, allowing circumferential and radial motion within the basal plane. However, given the crucial role of atrioventricular plane motion in ventricular function [172], allowing long-axis motion of the basal plane is desirable. To achieve this, other researchers have used Robin BCs, conceptually a spring and dashpot in parallel [83, 85, 197]. These BCs introduce additional parameters describing the strength of the elastic (spring) and viscous (dashpot) contributions; nominal values can be found in a recent cardiac mechanics benchmark paper [53]. Interestingly, the previously described BCs lead to physical inconsistencies, including a net force exerted by the blood on the chamber wall and an inaccurate value of the work done by the blood pressure to inflate the heart chamber. To address these issues, [200] proposed a so-called energy-consistent basal BC, which depends on the pressure being applied on the endocardial surface and permits global energy conservation when coupling to a 0D model of blood circulation [169, 201].

**Figure 5:**
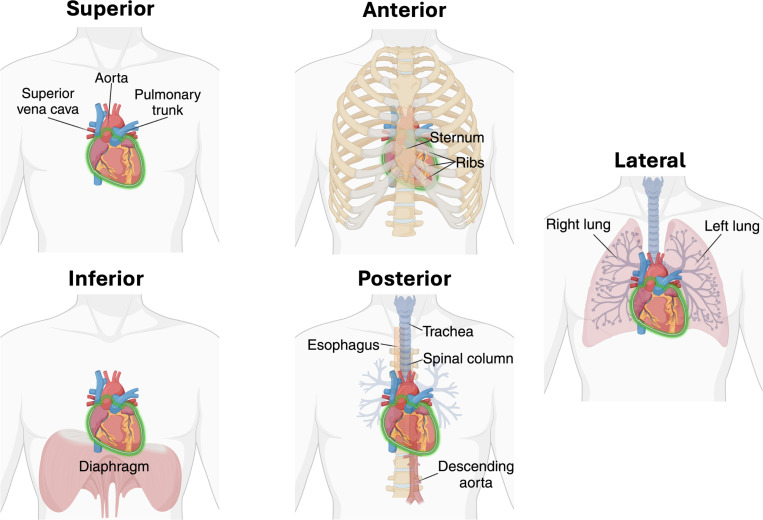
Illustrations showing the anatomic structures surrounding the heart, on the superior, inferior, anterior, posterior, and lateral sides. The pericardium is highlighted in green. Created in https://BioRender.com

**Pericardial BCs** As discussed in Section 2, the heart, as well as the roots of the great vessels, is enclosed in a fibrous sac called the pericardium [34]. A thin layer of pericardial fluid fills the space between the epicardium and the pericardium [172], constraining relative motion in the normal direction while allowing tangential sliding with relatively low friction [34]. Between the myocardium and visceral pericardium are fat deposits known as *epicardial* adipose tissue (EAT), which tend to occupy the grooves between the LV and RV, as well as between the ventricles and atria [202]. Additional fat deposits, known as *paracardial* adipose tissue, surround up to 80 % of the pericardium’s outer surface (parietal pericardium) [202]. Beyond the pericardium are various neighboring anatomic structures, illustrated in Figure 5. Superiorly, the pericardium is continuous with the walls of the great vessels. Anteriorly, sternopericardial ligaments attach it to the sternum, and it may contact the fourth to sixth ribs on the left side. Inferiorly, the pericardiophrenic ligaments connect it with the diaphragm. Posteriorly, the pericardium is adjacent to structures such as the bronchi, esophagus, descending thoracic aorta, and vertebral column [196]. Laterally, it is in contact with the lungs [34]. Given this complex anatomic environment, it is apparent that the tissue support on the epicardium is heterogeneous, and consequently very difficult to model.

Some groups have chosen to explicitly model the pericardium in cardiac simulations. [172] introduced a geometric model of the pericardium and surrounding tissue, solving a frictionless contact problem between the epicardial surface of a whole-heart mesh and the inner surface of a pericardial mesh. Performing simulations with and without pericardial support, they found that including the pericardium significantly influenced heart deformation and function, primarily by restricting motion of the outer contour of the heart, consistent with physiological observations. Additionally, pericardial support reduced systolic pressure and ejection fraction in both ventricles while enhancing atrioventricular plane displacement and consequently atrial filling. Similarly, [203] modeled the rat pericardium using shell finite elements and imposed frictionless sliding contact with the epicardium via a linear penalty method. Their study examined the effects of pericardial elasticity on cardiac function under pulmonary hypertension and simulated pericardiectomy by removing the pericardium entirely.

More commonly, however, the effect of the pericardium is incorporated through a BC on the epicardial surface, avoiding the computational expense of explicit contact modeling. Inspired by [172], [34] introduced a widely used pericardial BC, formulated as a Robin BC acting only in the epicardial normal direction. This penalizes normal displacement while permitting free tangential sliding, reflecting the lubricating role of the pericardial fluid. Comparing simulations with and without the pericardial BC, they found that the pericardial BC improved agreement with MRI data in terms of atrioventricular plane displacement, atrial filling during ventricular systole, and LV and RV endocardial motion.

Building on this approach, [81] introduced a spatially varying version of the pericardial Robin BC, further improving the agreement with image data. In their work, the stiffness parameter decreased from apex to base of the ventricles, based on observations of epicardial motion in CT images. This decrease in stiffness reflected the presence of EAT near the ventricular base, which locally reduces the constraining effect of the pericardium. Similarly, [62] assigned a higher stiffness value to epicardial regions directly contacting the pericardium and a much lower value to regions near the ventricular base and atrial appendages where EAT is more prominent. Further exploring this concept, [173] varied the spatial heterogeneity of the pericardial BC and found that while local deformations were very sensitive to changes in BC parameters, global functional metrics such as stroke volume remained relatively unaffected. In recent work, [204] proposed a method to incorporate localized forces on the RV, particularly from the ribs and diaphragm, into an inverse mechanics framework, significantly improving reference configuration estimates for the RV.

In a comparative study, [205] evaluated five different pericardial modeling approaches on the same whole-heart mesh, including the sliding contact method of [172] and the constant and spatially-varying Robin BC approaches of [34], [81], and [62]. Their results indicated that Robin BCs reduced LV twist compared to sliding contact models, likely due to their standard formulation being valid only for small epicardial rotations [34]. Additionally, they found Robin BCs to be more computationally demanding than the sliding contact approach, warranting further investigation.

An alternative approach to boundary conditions was explored by [175, 206], who used boundary energy terms to weakly impose the motion of the epicardial and basal surfaces based on image data. Comparing their approach to traditional generic boundary conditions, such as those described previously, they found significant improvement in model accuracy. While a promising approach, the utility of image-based BCs in a clinical setting should be carefully considered, since they “inherently do not accommodate for changes in mechanical environment upon performing (virtual) surgeries or (virtually) implanting a device” [33].

## 6 Coupling tissue mechanics to other physics

State-of-the-art heart models capture not only cardiac tissue mechanics, but also electrical signal propagation, subcellular force generation, and blood flow within the heart and throughout the broader circulatory system. In some cases, coupling these physical processes is essential to accurately represent key cardiac phenomena. However, such couplings can also introduce significant numerical challenges that require specialized solution strategies. Typically, multiphysics equations are coupled either monolithically or in a partitioned (segregated) manner: monolithic approaches offer greater robustness but can be complex to implement, whereas partitioned schemes are more modular and easier to develop but may suffer from numerical instabilities [197]. In this section, we review the physical processes commonly coupled with mechanics, the capabilities such couplings enable, their technical implementation, and the computational challenges they pose.

### 6.1 Electrophysiology and cellular contraction

The physical models governing cardiac electrophysiology and their interactions with mechanics are summarized in Figure 1. Electrical activity in the heart is commonly modeled using a reaction-diffusion partial differential equation (PDE) for the transmembrane electric potential, resulting in a propagating wave solution [207]. This formulation can represent differing conduction anisotropy ratios in the intracellular and extracellular spaces (bidomain model), or assume identical anisotropy in both domains (monodomain model) [208]. From a numerical standpoint, the bidomain model is more challenging due to the need to solve an implicit equation for the extracellular potential at every timestep. Computationally efficient alternative formulations like the Eikonal and Reaction-Eikonal models have also been explored [209].

Electrical activity is typically coupled with an ionic model: an ordinary differential equation (ODE) system describing the concentration dynamics of various ions that cross the cell membrane during depolarization and repolarization [62]. Distinct ionic models have been developed for specific cardiac cell types, including ventricular myocytes, atrial myocytes, and Purkinje cells [139]. These ionic models also compute the intracellular calcium ion (Ca^2+^) concentration, which is the primary input to a model for cellular contraction [55]. This contraction is linked to tissue-scale mechanics through one of three main strategies: active stress, active strain, or a combined approach known as Hill’s three-element model [54]. Alternatively, electrical activity may simply yield an activation time, which triggers active contraction in a phenomenological modeling approach [52, 57, 210, 211]. In many studies, a *weak* or one-way coupling from electrophysiology to mechanics, which captures so-called excitation-contraction coupling [212], is used to efficiently reproduce the essential features of cardiac electromechanics [47, 83, 209, 213].

*Strongly* coupled approaches to electromechanics account for physiological feedbacks from mechanical deformation to both cellular contraction and electrical activity. Many models of contraction incorporate length-dependent activation, wherein stretched muscle fibers produce greater contractile force, an effect underlying the organ-level Frank-Starling mechanism [55]. Some models also incorporate a dependence on the rate of deformation [214]. Recent studies have also examined the effects of mechanical deformation on electrophysiology, terms mechano-electric feedbacks (MEFs) [215], which include deformation-induced changes to the effective conductivity tensor and volume scaling of electrical current and potential [66, 121], as well as cell-level responses to stretch, such as the activation of stretch-sensitive ion channels [212, 216, 217]. MEFs are considered important in diseased cases [57, 218], and are believed to explain phenomena like commotio cordis—ventricular fibrillation induced by a sudden blow to the chest—or its converse, the precordial thump, which can restore normal rhythm [219].

Simulating coupled electrophysiology and mechanics introduces several numerical challenges. Electrophysiology often demands finer mesh resolution and smaller timesteps than mechanics to accurately reproduce physiological conduction velocities [139]. This discrepancy may necessitate variable interpolation between meshes and the use of specialized time-integration schemes [51, 62, 171, 220], depending on the specific numerical approach. These challenges are discussed further in Section 7. Moreover, mechanics to contraction feedbacks related to fiber stretch and stretch rate can destabilize staggered coupling schemes unless appropriate stabilization techniques are employed [221]. For further detail on models of electrophysiology and their coupling to mechanics, we refer the reader to the reviews in [222, 223].

### 6.2 Blood flow

At the endocardial and valvular surfaces, the motion and stress are continuous between the tissue and blood [38], resulting in a strong coupling between the two domains. This interaction is fundamental: blood flow within the heart is predominantly determined by the motion of the heart walls and valve leaflets, while the blood, in turn, imposes significant stresses on cardiac tissue. The interplay between blood flow (fluid) and myocardial mechanics (structure) falls under the general category of fluid–structure interaction (FSI).

Two primary approaches dominate the modeling of cardiac FSI: the immersed boundary (IB) method [174, 199, 224, 225] and the arbitrary Lagrangian–Eulerian (ALE) method [139, 226, 227]. In brief, the IB method solves the fluid equations on a fixed (Eulerian) background mesh, incorporating the effects of structures such as the endocardium or valves through localized forcing terms on the right-hand side of the fluid momentum equation. Because the fluid and structural meshes do not conform at the interface, IB approaches often rely on adaptive mesh refinement to maintain accuracy near these boundaries.

By contrast, the ALE method uses a fluid mesh that conforms to and deforms with the moving structure, offering enhanced resolution at the fluid–structure interface. However, this benefit comes at the cost of potential mesh distortion, often requiring remeshing strategies to accommodate large deformations [38]. A more comprehensive overview of cardiac FSI techniques and challenges can be found in [38].

Cardiac FSI models mark a significant advance toward developing cardiac digital twins, enabling the integration of mechanics, and often electrophysiology as well, with realistic simulations of blood flow within the cardiac chambers and across the valves. Blood flow modeling itself remains an expansive area of research with major clinical relevance. Nevertheless, FSI introduces unique computational challenges, which is why fully coupled, whole-heart FSI simulations have only recently become feasible.

### 6.3 Circulatory dynamics

While fluid–structure interaction (FSI) methods are frequently used to couple cardiac mechanics with blood flow within and proximal to the heart, they are rarely employed to model blood flow throughout the rest of the circulatory system. Instead, the systemic and pulmonary circulations are commonly represented using lumped parameter networks (LPNs), zero-dimensional (0D) models that analogize blood flow to electrical current [228] (1D models are also sometimes used [229]). This simplification allows for the computation of bulk hemodynamic quantities such as pressure and flow rate at a significantly reduced cost. Mathematically, LPNs are described by systems of differential-algebraic equations (DAEs) [230], which can be derived by applying Kirchhoff’s first law to an equivalent electrical circuit.

These DAE systems can be coupled to the three-dimensional (3D) blood flow equations via inlet/outlet flow rate and pressure [35, 228, 231], or directly to the tissue mechanics equations using chamber flow rate or volume and endocardial pressure as coupling variables (indicated by the dashed lines in Figure 1) [83, 169, 197, 201, 232, 233]. A wide range of LPN models have been proposed in the literature, from simple Windkessel-type representations [234] to elaborate, closed-loop networks modeling the entire circulatory system [201, 235].

Incorporating an LPN into a heart model enhances physiological fidelity by imposing preload and afterload [201, 236], and allows the study of cardiac responses to circulatory changes such as during exercise [237], under surgical conditions [238], or due to respiration [239]. For cardiac mechanics or FSI models that lack fully resolved valve dynamics, LPNs can also serve to model valve behavior, enabling physiologic reproduction of the cardiac cycle, especially the clear delineation of the four cardiac phases [197]. Furthermore, using an LPN, especially one representing the full circulatory loop, allows investigation of how cardiac function influences system-wide hemodynamics. Since patient-specific data are often available at various locations in the circulatory system, incorporating an LPN can support parameter tuning to improve personalization of cardiac models [5, 42, 240].

However, coupling heart models to LPNs introduces computational challenges akin to those encountered in FSI. Loosely coupled schemes can exhibit instabilities, rooted in the “balloon dilemma,” which must be addressed using specialized stabilization strategies [241]. In strongly coupled approaches employing Newton-like solvers, the inclusion of an LPN modifies the structure of the tangent matrix, necessitating the use of carefully constructed preconditioners [197, 242, 243].

### 6.4 Other physical processes

We conclude this section by noting that while electrophysiology, cellular contraction, tissue mechanics, blood flow, and circulatory dynamics represent the core physical processes modeled in multiphysics simulations of the heart, additional physiological phenomena have also been explored. Notable examples include myocardial perfusion [244, 245, 246, 247], transport of blood species like oxygen [240], metabolic/energetic function [248], coagulation [249], and the physiological regulation of arterial pressure via the baroreflex mechanism [250]. Future cardiac modeling approaches could be extended to include these phenomenon with increasing fidelity.

## 7 Numerical formulations

Numerical methods are essential for the investigation of cardiac mechanics, particularly under physiologically detailed and patient-specific settings. The inherently multiphysics and multiscale nature of cardiac modeling has driven significant research into advanced numerical methodologies.

We begin by summarizing common numerical choices in the literature, focusing on FEA for simulating cardiac tissue mechanics. Tetrahedral elements are the most common, including linear [62, 121, 161, 226], quadratic [171, 233], and quadratic-linear in mixed displacement-pressure formulations [80, 175], but hexahedral elements have also been used [5, 51, 251]. For mechanics, average elements edge sizes are typically between 1 mm and 5 mm [53, 62, 66, 67, 80, 115, 172, 174, 226], resulting in $O\left(10^{3}\right)$ to $O\left(10^{5}\right)$ elements for a biventricular mesh [53]; a useful guideline is to have at least two elements through the wall thickness [83]. Time integration is usually implicit – generalized-α [83, 85, 197], Newmark [121, 171, 172, 219], backward Euler [5, 62, 201, 251] – with timestep sizes O(1) ms, but some studies have used explicit methods [52, 66, 174], which require smaller timestep sizes $O\left(10^{-2}\right)$ ms. Some groups forgo time integration entirely by performing a quasi-static analysis [51, 233]. Implicit schemes require the solution of a nonlinear algebraic system at each timestep, and Newton’s method is almost universally used [83, 121, 172, 197, 201, 226, 251]. To solve the resulting linear system at each Newton iteration, the Generalized Minimal Residual method (GMRES) is common [40, 83, 121, 201, 226, 251], but direct methods, like LU decomposition [172] have also been applied. Linear system preconditioners, including Balancing Domain Decomposition by Constraints [251], Algebraic Multigrid [251], Additive Schwarz [40], and incomplete LU with/without thresholding [40, 240] have been used. Linear solvers and preconditioners are often used from packages like MUMPS, PETSc, and Trilinos [53]. Note that these solver settings apply only when simulating tissue mechanics. Other physics, notably electrophysiology, have different spatial and temporal discretization requirements [252], and can introduce particular nonlinear and linear solver challenges [62]. In the remainder of this section, we delve into these topics further, reviewing key modeling challenges in multiphysics heart modeling and the corresponding developments in numerical techniques designed to address them.

In modeling the passive mechanical behavior of myocardium, a major factor concerning the numerical design comes from the (quasi-)incompressibility of the material. This property essentially dictates the choice of the variational formulation and element technology. Conventionally, finite strain problems are formulated on the basis of the principle of stationary potential, wherein the bulk modulus is set to be substantially larger than the shear moduli to penalize the incompressibility condition [62, 121, 253]. However, that formulation, when paired with low-order elements, is known to suffer from mesh locking and pressure instability. To mitigate this issue, special element technologies have been developed, including the F-bar projection technique [254, 255] and higher-order elements [256, 257]. In particular, the Hermite cubic element has been particularly favored in studying cardiac mechanics [258, 259, 260]. Nonetheless, a persistent drawback of the penalty-based formulation is the resulting near-singular system, which poses a significant challenge for linear solvers. In many cases, robust direct solvers are required to ensure numerical stability.

Alternatively, the incompressibility constraint can be enforced via a Lagrange multiplier, introduced as an additional field that is stress-like and sometimes simply referred to as the pressure. This leads to the so-called two-field variational or mixed formulation, in which both displacement and pressure are treated as primary unknowns. Unlike the pure displacement formulation, this approach enforces the incompressibility constraint equation directly without suffering from the singular discrete problem. Its mathematical form is of the saddle-point nature and requires the use an element pair that satisfies the celebrated inf-sup condition [261]. Not all combinations of shape functions are inf-sup stable. The most common choice is a (tri-)quadratic interpolation for displacement and a (tri-)linear interpolation for the pressure, known as the Taylor-Hood element and denoted by $P_{2}/P_{1}$ for tetrahedra and $Q_{2}/Q_{1}$ for hexahedra [80, 175, 210, 262, 263]. However, using Taylor-Hood elements necessitates generating a higher-order mesh for the kinematic field. Moreover, higher-order $C^{0}$-continuous Lagrange elements are not sufficiently robust for finite strain analysis [264]. Consequently, low-order element pairs are favored in practice. A widely used low-order choice is trilinear displacement paired with piecewise constant pressure, denoted as the $Q_{1}/Q_{0}$ or $Q_{1}/P_{0}$ element [172, 262, 265]. Nevertheless, this element pair is marginally inf-sup stable, and may exhibit spurious pressure modes under certain loading conditions. Additionally, generating a full hexahedral mesh remains challenging for cardiac simulations [210], making tetrahedral elements more desirable, especially for patient-specific geometries [62, 121, 161, 226]. Unfortunately, the $P_{1}/P_{0}$ element is not inf-sup stable and exhibits volumetric locking, although it has been applied in cardiac simulations [254]. We refer readers to a systematic comparison of a variety of elements for finite strain analysis [266]. Higher-order elements are appealing because of their superior accuracy per degrees-of-freedom. Recent developments have been made with inspiration from isogeometric analysis, and higher-order, higher-continuity splines have have been demonstrated to be a robust and accurate element technology in modeling tissue behavior [51, 267]. Additionally, meshless methods, originally proposed to circumvent mesh distortion and meshing complexities, have demonstrated promising performance in cardiac electromechanical simulations [52]. To conclude, we mention that the variational formulation is sometimes further extended by introducing the dilatation as an independent field, leading to a three-field variational formulation [268].

The convenience of linear tetrahedral elements has spurred extensive research into stabilization strategies within the variational formulation. This line of development originated in computational fluid mechanics through the introduction of the Petrov-Galerkin formulation [269, 270]. It provides a foundation for circumventing the inf-sup condition by adding consistent stabilization terms. A rigorous analysis was performed that guarantees convergence using any combination of elements in the mixed formulation. Building on this, the variational multiscale (VMS) formulation was introduced as a unifying framework [271], in which the stabilization terms can be viewed as the subgrid scale models that approximate the impact of the unresolved parts of the solution on the discrete solution. In fluid dynamics, the VMS formulation not only offers a way of handling the pressure instability, but also provides an implicit large eddy simulation mechanism by modeling unresolved scales [272, 273]. To date, the VMS formulation forms a cornerstone for patient-specific hemodynamic simulations [274, 275]. Importantly, because the VMS formulation is derived from a Galerkin framework, it is well-suited to fluid-structure interaction (FSI) where both fluid and solid subdomains are discretized using the finite element method [272]. This compatibility has led to the widespread adoption of VMS-based FSI solvers in cardiac modeling, including simulations of ventricular mechanics [223, 226, 276, 277] and whole-heart modeling [227, 278].

A recent development in continuum modeling involves the use of the Gibbs free energy as the thermodynamic potential [279]. It leads to a unified continuum model capable of describing viscous fluid flow and finite strain deformation. This feature makes it appealing in designing numerical formulations for FSI as it enables consistent numerical treatment and monolithic coupling of the two sub-problems [280, 281]. When applied to finite strain problems, this approach yields a mixed formulation distinct from the classical two-field or multi-field variational principles. It incorporates compressible and incompressible material behaviors in a single mixed formulation through the introduction of an isothermal compressibility factor. For fully incompressible materials, the constraint naturally emerges as a divergence-free condition for the velocity, identical to the constraint in the Navier-Stokes equations. An appealing feature of the formulation is its embedded nonlinear stability [267], which has been leveraged to construct structure-preserving time integrators for long-term elastodynamic simulations [282]. Furthermore, one may directly invoke the VMS formulation to introduce pressure stabilization for the mixed formulation, which allows equal-order interpolation for the kinematic and pressure fields [279]. The effectiveness of the approach has been demonstrated in studies of a fiber-reinforced anisotropic hyperelastic material [267, 283]. Recently, this approach has been applied to cardiac mechanics [40, 53].

Additional numerical challenges come from the spatial and temporal multiscale nature of cardiac electromechanics. As discussed in Section 6.1, the electrophysiology problem is governed by a system of nonlinear reaction-diffusion equations, whose solution manifests as a propagating wave. During sinus rhythm, the action potential travels as a wavefront across the myocardium followed by a repolarization phase to return to its resting value. Capturing such wavefront dynamics imposes strict numerical requirements, as the steep gradients can easily give rise to spurious undershoots and overshoots. Consequently, numerical stability becomes a primary design concern. Typically, a relatively fine spatial resolution is needed to capture the rapid voltage upstroke and conduction for cardiac electrophysiology [254]. Typical mesh element sizes are on the order of a few hundred microns. In contrast, the mechanical deformation of the ventricular myocardium is smoother, allowing for a coarser mesh resolution. A straightforward approach is to use a single mesh with resolution sufficiently high for electrophysiology. An obvious drawback is that this leads to an excessive number of degrees of freedom for the mechanics problem $O\left(10^{8}\right)$ degrees-of-freedom for the human heart). To address this mismatch, several multiscale strategies have been proposed. One class of approaches employs adaptive mesh refinement to dynamically resolve the wavefront [284]. Another class adopts a multi-mesh strategy, where separate meshes with different resolutions are used for the electrical and mechanical subproblems [220, 285, 286]. In such cases, the electrophysiology data needs to be interpolated to the mechanics mesh during the simulation. If the two meshes are nested with the finer mesh generated by refining the coarse mesh, the interpolation across the two meshes is relatively straightforward [5, 287]. However, generating such nested meshes demands extra care due to the geometrical and functional heterogeneity of the heart. If the two meshes are generated independently and do not share nodal alignment, more sophisticated projection techniques are necessary to transfer physical fields. For example, an intergrid transfer operator based on the radial basis function has been proposed, demonstrating superior accuracy and scalability [220].

The temporal multiscale phenomena also plays a critical role in the numerical treatment of cardiac electromechanics. The semi-discrete problem of electrophysiology exhibits significant stiffness in time [288]. To accurately resolve these fast transients, time marching must be performed using very small time step size, typically on the order of sub-milliseconds. In contrast, the mechanical responses can be adequately characterized with a time step size of around one millisecond, especially when using implicit time integration [83, 85, 197]. The temporal disparity strongly influences the design of coupling strategies, often requiring a balance between stability and computational efficiency. In monolithic approaches, one formulates a single large system of equations, encompassing both electrical and mechanical sub-problems, permitting stable solutions with larger time steps [289, 290]. However, solving the resulting large-scale nonlinear system efficiently necessitates careful design of robust linear solvers and preconditioners [291]. Moreover, a straightforward monolithic integration with a single global time step is often computationally prohibitive due to fine temporal resolution set by the electrophysiology. To address this, operator-splitting strategies are frequently employed [292]. In such schemes, the mechanical problem is advanced using a large time step, while the electrophysiology is integrated by subcycling using smaller time steps within each mechanical step. This approach effectively exploits the temporal scale separation and improves computational efficiency. Additionally, multiscale behavior is present within the electrophysiology model itself. Fortunately, the reaction–diffusion nature of the governing equations allows the stiff ionic reactions and the non-stiff diffusion terms to be integrated separately [293].

To conclude, we summarize prominent open-source software frameworks in the cardiac modeling community. Continuity, as a pioneering software environment for cardiac modeling, employs the finite element to model cardiac electromechanics [260]. Besides conventional finite element technology, it is also supports Hermite spline basis functions. Chaste is a highly object-oriented library for computational biology, featuring a well-validated cardiac modeling module [294]. The finite element library life^x^ [295], built on top of deal.II [296], has undergone continuous development to incorporate detailed electrophysiology [32], tissue mechanics [297], and blood flow [298]. The SimVascular project [274] offers a complete pipeline for patient-specific cardiovascular modeling. Its new module, svMultiPhysics (https://github.com/SimVascular/svMultiPhysics), builds on its Fortran-based predecessor svFSI [299], providing an efficient MPI-parallelized C++ solver with multiphysics capabilities, including electrophysiology and fluid–structure interaction. It also supports advanced linear algebra libraries such as Trilinos [300] and PETSc [301]. Another notable framework is CHeart, which offers comprehensive multiphysics capabilities specifically tailored for cardiac simulations [302]. Other notable open-source multiphysics codes include 4C [303], written in C++ and originating from the in-house BACI code [85] at the Technical University of Munich, and Ambit [304], built on top of the Python-based FEniCS library. FEBio [305], a multiphysics software tool for general biomechanics, has also been used to study the heart. For electrophysiology, openCARP [306] is a very popular open-source project. We also highlight SlicerHeart [307], an open-source extension for 3D Slicer [308] designed for advanced image processing, visualization, and (non-simulation) quantitative analysis for cardiovascular medicine. Alongside software packages, the community has developed benchmark problems to validate and compare numerical methods [53, 262], which are essential for evaluating the performance of different techniques.

## 8 Concluding remarks

In this review, we discussed recent advances and open questions in the field of cardiac mechanics modeling, which we believe to be important to the development of cardiac digital twins as predictive clinical tools. In Section 2, we first discussed the anatomy of the heart and the methods used to create cardiac anatomic models and meshes, which is typically the first step in any heart model. Historically, patient-specific models have required labor-intensive manual segmentation of medical images, but recent advances in machine learning are accelerating the process, in both healthy and diseased cases. Next, in Section 3, we reviewed the mesostructure of the heart, discussing how it is measured experimentally and modeled computationally, and highlighting some ambiguities we believe are important for modelers to be aware of. In Section 4, we summarized recent developments in three aspects of myocardial constitutive modeling – viscosity, compressibility, and fiber dispersion, and discussed how they influence simulation results. We then reviewed commonly used anatomic model geometries and boundary conditions in Section 5. Excluding certain cardiac structures from the computational domain, and the boundary conditions necessitated by such exclusions, can have significant impacts on model predictions. In Section 6, we briefly discussed how tissue mechanics is coupled to other physical processes in the heart, including electrophysiology, blood flow, and circulatory dynamics. Adding these couplings usually increase the physiological fidelity of heart models, but inevitably introduces additional computational challenges. Finally, in Section 7, we reviewed key topics in the design of numerical formulations for multiphysics simulations of the heart.

Several important topics fell outside the scope of this review, but we would nonetheless be remiss in not mentioning them. Much work has been done to accelerate simulations to make them applicable on clinical timescales. Many groups parallelize various aspects of their codes to run on high performance computing (HPC) clusters [62, 66, 299] or with GPU acceleration [309]. Other groups have targeted the underlying mathematical formulation [310] and preconditioners [251, 242] to gain speedup. However, these efforts to accelerate simulations are dwarfed in comparison to machine learning approaches, which have seen explosive growth in recent years [200, 311, 312, 313, 314, 315, 316, 317, 318]. Cardiac growth and remodeling, the process by which cardiac morphology and structure change in response to mechanical stimuli on long timescales [57, 319], is also an important component of predictive cardiac medicine under development. We point the reader to recent works in [108, 320, 321, 322, 323], and in particular to the reviews in Niestrawska et al. [36] and Holzapfel et al. [324]. Finally, patient-specific modeling, encompassing methods to determine parameter values or other simulation inputs to match continuously-collected experimental or clinical data, are essential to making cardiac digital twins a reality [1, 3]. We highlight recent works that aim to determine constitutive parameters [40, 48, 52, 206, 325, 326], unloaded configurations [40, 84], electrophysiology parameters [5, 327, 240], cellular contraction parameters [213], and circulatory model parameters [42, 52]. Sensitivity analyses and parameter estimation for multiphysics heart models can be found in recent works [20, 41, 42, 328]. Reviews of patient-specific modeling can be found in Bracamonte et al. [56], Peirlinck et al. [63], and Schwarz et al. [329].

As demonstrated throughout this review, much of the computational groundwork for cardiac digital twins has already been laid, providing a strong foundation for future development. The pressing challenge now is to establish their clinical utility, an ambitious goal that will face hurdles in both validation [328] and regulatory approval [57]. Establishing the utility of cardiac digital twins is likely to reveal the key limitations that still constrain model accuracy and robustness. These may include, for instance, incomplete representations of myocardial mesostructure, imprecise pericardial boundary conditions, limitations in constitutive modeling, and suboptimal strategies for parameter estimation. Beyond these technical issues, practical barriers, such as the time required to translate medical imaging data into actionable simulation results, may further hinder clinical adoption. At the same time, rigorous validation could help clarify which model complexities are essential for clinical relevance, and which may be safely simplified. It remains an open question whether highly detailed, multiphysics, multiscale whole-heart models are necessary for most use cases, or whether simplified models, possibly accelerated by machine learning methods, offer a more pragmatic path forward. To move the field forward, community-wide efforts such as a computational medicine challenge for cardiac modeling, akin to the 2015 International Aneurysm CFD Challenge [330], could play a pivotal role in consolidating modeling practices and benchmarking performance.

## Footnotes

1 The color gradient from apex to base is difficult to discern because ${\text{Ψ}}_{\text{ab}}$ rises sharply from zero at the apex over a very short distance (not visible from this angle), reaching values close to one across most of the domain. This can be problematic in some applications. A common remedy, as in the universal ventricular coordinates framework [97] is to smooth ${\text{Φ}}_{\text{ab}}$ by mapping it onto a geodesic from apex to base.

2 Note that there is a typo in the original Holzapfel-Ogden paper. The isotropic term should read $e^{b\left(I_{1}-3\right)}-1$, but −1 is missing from the original paper [54, 85]. However, because only the derivative of ψ with respect to the deformation, i.e., the stress, is physically meaningful, the omission is inconsequential. Also, while the original paper does not include a Heaviside function, it contains an equivalent statement that the fiber and sheetlet terms should be included in the strain energy only when $I_{4,f}>1$ and $I_{4,s}>1$, respectively.

## References

[1] Thangaraj Phyllis M, Benson Sean H, Oikonomou Evangelos K, Asselbergs Folkert W, and Khera Rohan. Cardiovascular care with digital twin technology in the era of generative artificial intelligence. European Heart Journal, 45(45):4808–4821, December 2024. ISSN 0195-668X. doi:10.1093/eurheartj/ehae619. URL https://doi.org/10.1093/eurheartj/ehae619. 39322420 PMC11638093

[2] Coorey Genevieve, Figtree Gemma A., Fletcher David F., Snelson Victoria J., Vernon Stephen Thomas, Winlaw David, Grieve Stuart M., McEwan Alistair, Yang Jean Yee Hwa, Qian Pierre, O’Brien Kieran, Orchard Jessica, Kim Jinman, Patel Sanjay, and Redfern Julie. The health digital twin to tackle cardiovascular disease—a review of an emerging interdisciplinary field. npj Digital Medicine, 5(1):126, August 2022. ISSN 2398-6352. doi:10.1038/s41746-022-00640-7. URL https://www.nature.com/articles/s41746-022-00640-7. Publisher: Nature Publishing Group. 36028526 PMC9418270

[3] Sel Kaan, Osman Deen, Zare Fatemeh, Shahrbabak Sina Masoumi, Brattain Laura, Hahn Jin-Oh, Inan Omer T., Mukkamala Ramakrishna, Palmer Jeffrey, Paydarfar David, Pettigrew Roderic I., Quyyumi Arshed A., Telfer Brian, and Jafari Roozbeh. Building Digital Twins for Cardiovascular Health: From Principles to Clinical Impact. Journal of the American Heart Association, 13(19):e031981, October 2024. ISSN 2047-9980. doi:10.1161/JAHA.123.031981. URL https://www.ahajournals.org/doi/10.1161/JAHA.123.031981. PMC11681439 39087582

[4] Meiburg Roel, Rijks Jesse H J, Beela Ahmed S, Bressi Edoardo, Grieco Domenico, Delhaas Tammo, Luermans Justin G LM, Prinzen Frits W, Vernooy Kevin, and Lumens Joost. Comparison of novel ventricular pacing strategies using an electro-mechanical simulation platform. EP Europace, 25(6):euad144, June 2023. ISSN 1099-5129. doi:10.1093/europace/euad144. URL https://doi.org/10.1093/europace/euad144. PMC10259067 37306315

[5] Capuano Emilia, Regazzoni Francesco, Maines Massimiliano, Fornara Silvia, Locatelli Vanessa, Catanzariti Domenico, Stella Simone, Nobile Fabio, Del Greco Maurizio, and Vergara Christian. Personalized computational electro-mechanics simulations to optimize cardiac resynchronization therapy. Biomechanics and Modeling in Mechanobiology, August 2024. ISSN 1617-7940. doi:10.1007/s10237-024-01878-8. URL https://doi.org/10.1007/s10237-024-01878-8. PMC11554892 39192164

[6] Lee Lik Chuan, Ge Liang, Zhang Zhihong, Pease Matthew, Nikolic Serjan D., Mishra Rakesh, Ratcliffe Mark B., and Guccione Julius M.. Patient-specific finite element modeling of the Cardiokinetix Parachute^®^ device: effects on left ventricular wall stress and function. Medical & Biological Engineering & Computing, 52(6): 557–566, June 2014. ISSN 1741-0444. doi:10.1007/s11517-014-1159-5. URL https://doi.org/10.1007/s11517-014-1159-5. 24793158 PMC4118670

[7] Kung Ethan, Farahmand Masoud, and Gupta Akash. A Hybrid Experimental-Computational Modeling Framework for Cardiovascular Device Testing. Journal of Biomechanical Engineering, 141(051012), March 2019. ISSN 0148-0731. doi:10.1115/1.4042665. URL https://doi.org/10.1115/1.4042665. 30698632

[8] Seo Jongmin, Ramachandra Abhay B., Boyd Jack, Marsden Alison L., and Kahn Andrew M.. Computational evaluation of venous graft geometries in coronary artery bypass surgery. Seminars in thoracic and cardiovascular surgery, 34(2):521–532, 2022. ISSN 1043-0679. doi:10.1053/j.semtcvs.2021.03.007. URL https://www.ncbi.nlm.nih.gov/pmc/articles/PMC8429518/. 33711465 PMC8429518

[9] Dowling Cameron, Firoozi Sami, and Brecker Stephen J.. First-in-Human Experience With Patient-Specific Computer Simulation of TAVR in Bicuspid Aortic Valve Morphology. JACC: Cardiovascular Interventions, 13(2):184–192, January 2020. ISSN 1936-8798. doi:10.1016/j.jcin.2019.07.032. URL https://www.sciencedirect.com/science/article/pii/S1936879819316115. 31629752

[10] Vigil Chad, Lasso Andras, Ghosh Reena, Pinter Csaba, Cianciulli Alana, Nam Hannah H., Abid Ashraful, Herz Christian, Mascio Christopher E., Chen Jonathan, Fuller Stephanie, Whitehead Kevin, and Jolley Matthew A.. Modeling Tool for Rapid Virtual Planning of the Intracardiac Baffle in Double Outlet Right Ventricle. The Annals of thoracic surgery, 111(6):2078–2083, June 2021. ISSN 0003-4975. doi:10.1016/j.athoracsur.2021.02.058. URL https://www.ncbi.nlm.nih.gov/pmc/articles/PMC8154721/. 33689734 PMC8154721

[11] O’Hara Ryan P, Binka Edem, Prakosa Adityo, Zimmerman Stefan L, Cartoski Mark J, Abraham M Roselle, Lu DaiYin, Boyle Patrick M, and Trayanova Natalia A. Personalized computational heart models with T1-mapped fibrotic remodeling predict sudden death risk in patients with hypertrophic cardiomyopathy. eLife, 11:e73325, January 2022. ISSN 2050-084X. doi:10.7554/eLife.73325. URL https://doi.org/10.7554/eLife.73325. Publisher: eLife Sciences Publications, Ltd. PMC8789259 35076018

[12] Kovacheva Ekaterina, Gerach Tobias, Schuler Steffen, Ochs Marco, Dössel Olaf, and Loewe Axel. Causes of altered ventricular mechanics in hypertrophic cardiomyopathy: an in-silico study. BioMedical Engineering OnLine, 20(1):69, July 2021. ISSN 1475-925X. doi:10.1186/s12938-021-00900-9. URL https://doi.org/10.1186/s12938-021-00900-9. 34294108 PMC8296558

[13] Teitgen Abigail E., Hock Marcus T., McCabe Kimberly J., Childers Matthew C., Huber Gary A., Marzban Bahador, Beard Daniel A., McCammon J. Andrew, Regnier Michael, and McCulloch Andrew D.. Multiscale modeling shows how 2’-deoxy-ATP rescues ventricular function in heart failure. Proceedings of the National Academy of Sciences, 121(35):e2322077121, August 2024. doi:10.1073/pnas.2322077121. URL https://www.pnas.org/doi/abs/10.1073/pnas.2322077121. Publisher: Proceedings of the National Academy of Sciences. PMC11363293 39172779

[14] Gonzalo Alejandro, Augustin Christoph M., Bifulco Savannah F., Telle Åshild, Chahine Yaacoub, Kassar Ahmad, Guerrero-Hurtado Manuel, Durán Eduardo, Martínez-Legazpi Pablo, Flores Oscar, Bermejo Javier, Plank Gernot, Akoum Nazem, Boyle Patrick M., and del Alamo Juan C.. Multiphysics simulations reveal haemodynamic impacts of patient-derived fibrosis-related changes in left atrial tissue mechanics. The Journal of Physiology, 602(24):6789–6812, 2024. ISSN 1469-7793. doi:10.1113/JP287011. URL https://onlinelibrary.wiley.com/doi/abs/10.1113/JP287011. _eprint: https://onlinelibrary.wiley.com/doi/pdf/10.1113/JP287011. 39513553 PMC11652225

[15] Cairelli Adriana Gaia, Gendernalik Alex, Chan Wei Xuan, Nguyen Phuc, Vermot Julien, Lee Juhyun, Bark David, and Yap Choon Hwai. Role of tissue biomechanics in the formation and function of myocardial trabeculae in zebrafish embryos. The Journal of Physiology, 602(4):597–617, February 2024. ISSN 1469-7793. doi:10.1113/JP285490. 38345870 PMC13410689

[16] Sahli-Costabal Francisco, Seo Kinya, Ashley Euan, and Kuhl Ellen. Classifying Drugs by their Arrhythmogenic Risk Using Machine Learning. Biophysical Journal, 118(5):1165–1176, March 2020. ISSN 0006-3495. doi:10.1016/j.bpj.2020.01.012. URL https://www.sciencedirect.com/science/article/pii/S0006349520300382. 32023435 PMC7063479

[17] Peirlinck M., Yao J., Sahli Costabal F., and Kuhl E.. How drugs modulate the performance of the human heart. Computational Mechanics, 69(6):1397–1411, June 2022. ISSN 1432-0924. doi:10.1007/s00466-022-02146-1. URL https://doi.org/10.1007/s00466-022-02146-1.

[18] Kong Fanwei, Wilson Nathan, and Shadden Shawn. A deep-learning approach for direct whole-heart mesh reconstruction. Medical Image Analysis, 74:102222, December 2021. ISSN 1361-8415. doi:10.1016/j.media.2021.102222. URL https://www.sciencedirect.com/science/article/pii/S136184152100267X. PMC9503710 34543913

[19] Alnasser Turki Nasser, Abdulaal Lojain, Maiter Ahmed, Sharkey Michael, Dwivedi Krit, Salehi Mahan, Garg Pankaj, Swift Andrew James, and Alabed Samer. Advancements in cardiac structures segmentation: a comprehensive systematic review of deep learning in CT imaging. Frontiers in Cardiovascular Medicine, 11, January 2024. ISSN 2297-055X. doi:10.3389/fcvm.2024.1323461. URL https://www.frontiersin.org/journals/cardiovascular-medicine/articles/10.3389/fcvm.2024.1323461/full. Publisher: Frontiers. PMC10839106 38317865

[20] Shi Lei, Chen Ian Y., and Vedula Vijay. Personalized Multiscale Modeling of Left Atrial Mechanics and Blood Flow, May 2025. URL https://www.biorxiv.org/content/10.1101/2025.04.26.650771v1. Pages: 2025.04.26.650771 Section: New Results.

[21] Fedele Marco and Quarteroni Alfio. Polygonal surface processing and mesh generation tools for the numerical simulation of the cardiac function. International Journal for Numerical Methods in Biomedical Engineering, 37(4):e3435, 2021. ISSN 2040-7947. doi:10.1002/cnm.3435. URL https://onlinelibrary.wiley.com/doi/abs/10.1002/cnm.3435. _eprint: https://onlinelibrary.wiley.com/doi/pdf/10.1002/cnm.3435. 33415829 PMC8244076

[22] Wilson Alexander J., Sands Gregory B., LeGrice Ian J., Young Alistair A., and Ennis Daniel B.. Myocardial mesostructure and mesofunction. American Journal of Physiology-Heart and Circulatory Physiology, 323(2): H257–H275, August 2022. ISSN 0363-6135. doi:10.1152/ajpheart.00059.2022. URL https://journals.physiology.org/doi/full/10.1152/ajpheart.00059.2022. Publisher: American Physiological Society. 35657613 PMC9273275

[23] Dall’Armellina Erica, Ennis Daniel B., Axel Leon, Croisille Pierre, Ferreira Pedro F., Gotschy Alexander, Lohr David, Moulin Kevin, Nguyen Christopher T., Nielles-Vallespin Sonja, Romero William, Scott Andrew D., Stoeck Christian, Teh Irvin, Tunnicliffe Elizabeth M., Viallon Magalie, Wang Victoria, Young Alistair A., Schneider Jürgen E., and Sosnovik David E.. Cardiac diffusion-weighted and tensor imaging: A consensus statement from the special interest group of the Society for Cardiovascular Magnetic Resonance. Journal of Cardiovascular Magnetic Resonance, 27(1):101109, 2025. ISSN 10976647. doi:10.1016/j.jocmr.2024.101109. URL https://linkinghub.elsevier.com/retrieve/pii/S1097664724011360. PMC11759557 39442672

[24] Piersanti Roberto, Africa Pasquale C., Fedele Marco, Vergara Christian, Luca Dedè Antonio F. Corno, and Quarteroni Alfio. Modeling cardiac muscle fibers in ventricular and atrial electrophysiology simulations. Computer Methods in Applied Mechanics and Engineering, 373:113468, January 2021. ISSN 00457825. doi:10.1016/j.cma.2020.113468. URL https://linkinghub.elsevier.com/retrieve/pii/S0045782520306538.

[25] Costabal Francisco Sahli, Hurtado Daniel E., and Kuhl Ellen. Generating Purkinje networks in the human heart. Journal of Biomechanics, 49(12):2455–2465, August 2016. ISSN 0021-9290. doi:10.1016/j.jbiomech.2015.12.025. URL https://www.sciencedirect.com/science/article/pii/S0021929015007332. 26748729 PMC4917481

[26] Barber Fernando, Langfield Peter, Lozano Miguel, García-Fernández Ignacio, Duchateau Josselin, Hocini Mélèze, Haïssaguerre Michel, Vigmond Edward, and Sebastian Rafael. Estimation of Personalized Minimal Purkinje Systems From Human Electro-Anatomical Maps. IEEE Transactions on Medical Imaging, 40(8): 2182–2194, August 2021. ISSN 1558-254X. doi:10.1109/TMI.2021.3073499. URL https://ieeexplore.ieee.org/abstract/document/9405625. 33856987

[27] Camps Julia, Berg Lucas Arantes, Wang Zhinuo Jenny, Sebastian Rafael, Riebel Leto Luana, Doste Ruben, Zhou Xin, Sachetto Rafael, Coleman James, Lawson Brodie, Grau Vicente, Burrage Kevin, Bueno-Orovio Alfonso, dos Santos Rodrigo Weber, and Rodriguez Blanca. Digital twinning of the human ventricular activation sequence to Clinical 12-lead ECGs and magnetic resonance imaging using realistic Purkinje networks for in silico clinical trials. Medical Image Analysis, 94:103108, May 2024. ISSN 1361-8415. doi:10.1016/j.media.2024.103108. URL https://www.sciencedirect.com/science/article/pii/S1361841524000331. 38447244

[28] Emendi Monica, Sturla Francesco, Ghosh Ram P., Bianchi Matteo, Piatti Filippo, Pluchinotta Francesca R., Giese Daniel, Lombardi Massimo, Redaelli Alberto, and Bluestein Danny. Patient-Specific Bicuspid Aortic Valve Biomechanics: A Magnetic Resonance Imaging Integrated Fluid–Structure Interaction Approach. Annals of Biomedical Engineering, 49(2):627–641, February 2021. ISSN 1573-9686. doi:10.1007/s10439-020-02571-4. URL https://link.springer.com/article/10.1007/s10439-020-02571-4. Company: Springer Distributor: Springer Institution: Springer Label: Springer Number: 2 Publisher: Springer International Publishing. 32804291 PMC7990108

[29] Haj-Ali Rami, Marom Gil, Ben Zekry Sagit, Rosenfeld Moshe, and Raanani Ehud. A general three-dimensional parametric geometry of the native aortic valve and root for biomechanical modeling. Journal of Biomechanics, 45(14):2392–2397, September 2012. ISSN 0021-9290. doi:10.1016/j.jbiomech.2012.07.017. URL https://www.sciencedirect.com/science/article/pii/S0021929012004125. 22854206

[30] Kaiser Alexander D., Shad Rohan, Hiesinger William, and Marsden Alison L.. A design-based model of the aortic valve for fluid-structure interaction. Biomechanics and Modeling in Mechanobiology, 20(6):2413–2435, December 2021. ISSN 1617-7940. doi:10.1007/s10237-021-01516-7. URL https://link.springer.com/article/10.1007/s10237-021-01516-7. Company: Springer Distributor: Springer Institution: Springer Label: Springer Number: 6 Publisher: Springer Berlin Heidelberg. 34549354 PMC10752438

[31] Pagani Stefano, Dede’ Luca, Manzoni Andrea, and Quarteroni Alfio. Data integration for the numerical simulation of cardiac electrophysiology. Pacing and Clinical Electrophysiology, 44(4):726–736, 2021. ISSN 1540-8159. doi:10.1111/pace.14198. URL https://onlinelibrary.wiley.com/doi/abs/10.1111/pace.14198. _eprint: https://onlinelibrary.wiley.com/doi/pdf/10.1111/pace.14198. 33594761 PMC8252775

[32] Africa Pasquale Claudio, Piersanti Roberto, Regazzoni Francesco, Bucelli Michele, Salvador Matteo, Fedele Marco, Pagani Stefano, Dede’ Luca, and Quarteroni Alfio. lifex-ep: a robust and efficient software for cardiac electrophysiology simulations. BMC Bioinformatics, 24(1):389, October 2023. ISSN 1471-2105. doi:10.1186/s12859-023-05513-8. URL https://doi.org/10.1186/s12859-023-05513-8. 37828428 PMC10571323

[33] Peirlinck Mathias, Sack Kevin L., De Backer Pieter, Morais Pedro, Segers Patrick, Franz Thomas, and De Beule Matthieu. Kinematic boundary conditions substantially impact in silico ventricular function. International Journal for Numerical Methods in Biomedical Engineering, 35(1):e3151, 2019. ISSN 2040-7947. doi:10.1002/cnm.3151. URL https://onlinelibrary.wiley.com/doi/abs/10.1002/cnm.3151. _eprint: https://onlinelibrary.wiley.com/doi/pdf/10.1002/cnm.3151. 30188608

[34] Pfaller Martin R., Hörmann Julia M., Weigl Martina, Nagler Andreas, Chabiniok Radomir, Bertoglio Cristóbal, and Wall Wolfgang A.. The importance of the pericardium for cardiac biomechanics: from physiology to computational modeling. Biomechanics and Modeling in Mechanobiology, 18(2):503–529, April 2019. ISSN 1617-7940. doi:10.1007/s10237-018-1098-4. URL https://doi.org/10.1007/s10237-018-1098-4. 30535650

[35] Vignon-Clementel Irene E., Figueroa C. Alberto, Jansen Kenneth E., and Taylor Charles A.. Outflow boundary conditions for three-dimensional finite element modeling of blood flow and pressure in arteries. Computer Methods in Applied Mechanics and Engineering, 195(29):3776–3796, June 2006. ISSN 0045-7825. doi:10.1016/j.cma.2005.04.014. URL https://www.sciencedirect.com/science/article/pii/S0045782505002586.

[36] Niestrawska Justyna A., Augustin Christoph M., and Plank Gernot. Computational modeling of cardiac growth and remodeling in pressure overloaded hearts—Linking microstructure to organ phenotype. Acta Biomaterialia, 106:34–53, April 2020. ISSN 1742-7061. doi:10.1016/j.actbio.2020.02.010. URL https://www.sciencedirect.com/science/article/pii/S1742706120300891. 32058078 PMC7311197

[37] Michler C., Cookson A. N., Chabiniok R., Hyde E., Lee J., Sinclair M., Sochi T., Goyal A., Vigueras G., Nordsletten D. A., and P Smith N.. A computationally efficient framework for the simulation of cardiac perfusion using a multi-compartment Darcy porous-media flow model. International Journal for Numerical Methods in Biomedical Engineering, 29(2):217–232, 2013. ISSN 2040-7947. doi:10.1002/cnm.2520. URL https://onlinelibrary.wiley.com/doi/abs/10.1002/cnm.2520. _eprint: https://onlinelibrary.wiley.com/doi/pdf/10.1002/cnm.2520. 23345266

[38] Le Trung Bao, Usta Mustafa, Aidun Cyrus, Yoganathan Ajit, and Sotiropoulos Fotis. Computational Methods for Fluid-Structure Interaction Simulation of Heart Valves in Patient-Specific Left Heart Anatomies. Fluids, 7(3): 94, March 2022. ISSN 2311-5521. doi:10.3390/fluids7030094. URL https://www.mdpi.com/2311-5521/7/3/94. Number: 3 Publisher: Multidisciplinary Digital Publishing Institute.

[39] Tonini Andrea, Regazzoni Francesco, Salvador Matteo, Dede’ Luca, Scrofani Roberto, Fusini Laura, Cogliati Chiara, Pontone Gianluca, Vergara Christian, and Quarteroni Alfio. Two New Calibration Techniques of Lumped-Parameter Mathematical Models for the Cardiovascular System. International Journal for Numerical Methods in Engineering, 126(1):e7648, 2025. ISSN 1097-0207. doi:10.1002/nme.7648. URL https://onlinelibrary.wiley.com/doi/abs/10.1002/nme.7648. _eprint: https://onlinelibrary.wiley.com/doi/pdf/10.1002/nme.7648.

[40] Shi Lei, Chen Ian Y., Takayama Hiroo, and Vedula Vijay. An optimization framework to personalize passive cardiac mechanics. Computer Methods in Applied Mechanics and Engineering, 432:117401, December 2024. ISSN 0045-7825. doi:10.1016/j.cma.2024.117401. URL https://www.sciencedirect.com/science/article/pii/S004578252400656X.

[41] Strocchi Marina, Augustin Christoph M, Gsell Matthias A F, Rinaldi Christopher A, Vigmond Edward J, Plank Gernot, Oates Chris J, Wilkinson Richard D, and Niederer Steven A. Integrating imaging and invasive pressure data into a multi-scale whole-heart model. Journal of Biomechanical Engineering, pages 1–52, August 2025. ISSN 0148-0731. doi:10.1115/1.4069497. URL https://doi.org/10.1115/1.4069497. 40847594

[42] Salvador Matteo, Regazzoni Francesco, Dede’ Luca, and Quarteroni Alfio. Fast and robust parameter estimation with uncertainty quantification for the cardiac function. Computer Methods and Programs in Biomedicine, 231: 107402, April 2023. ISSN 0169-2607. doi:10.1016/j.cmpb.2023.107402. URL https://www.sciencedirect.com/science/article/pii/S016926072300069X. 36773593

[43] Shi Lei, Chen Yurui, and Vedula Vijay. HeartSimSage: Attention-Enhanced Graph Neural Networks for Accelerating Cardiac Mechanics Modeling, April 2025. URL http://arxiv.org/abs/2504.18968. arXiv:2504.18968 [physics]. 10.1016/j.jcp.2026.114895 PMC13160281 42125631

[44] Caforio Federica, Regazzoni Francesco, Pagani Stefano, Karabelas Elias, Augustin Christoph, Haase Gundolf, Plank Gernot, and Quarteroni Alfio. Physics-informed neural network estimation of material properties in soft tissue nonlinear biomechanical models. Computational Mechanics, 75(2):487–513, February 2025. ISSN 1432-0924. doi:10.1007/s00466-024-02516-x. URL https://link.springer.com/article/10.1007/s00466-024-02516-x. Company: Springer Distributor: Springer Institution: Springer Label: Springer Number: 2 Publisher: Springer Berlin Heidelberg.

[45] Höfler Matthias, Regazzoni Francesco, Pagani Stefano, Karabelas Elias, Augustin Christoph, Haase Gundolf, Plank Gernot, and Caforio Federica. Physics-informed neural network estimation of active material properties in time-dependent cardiac biomechanical models, May 2025. URL http://arxiv.org/abs/2505.03382. arXiv:2505.03382 [cs].

[46] Levrero-Florencio F., Margara F., Zacur E., Bueno-Orovio A., Wang Z. J., Santiago A., Aguado-Sierra J., Houzeaux G., Grau V., Kay D., Vázquez M., Ruiz-Baier R., and Rodriguez B.. Sensitivity analysis of a strongly-coupled human-based electromechanical cardiac model: Effect of mechanical parameters on physiologically relevant biomarkers. Computer Methods in Applied Mechanics and Engineering, 361:112762, April 2020. ISSN 0045-7825. doi:10.1016/j.cma.2019.112762. URL https://www.sciencedirect.com/science/article/pii/S0045782519306541. 32565583 PMC7299076

[47] Strocchi Marina, Longobardi Stefano, Augustin Christoph M., Gsell Matthias A. F., Petras Argyrios, Rinaldi Christopher A., Vigmond Edward J., Plank Gernot, Oates Chris J., Wilkinson Richard D., and Niederer Steven A.. Cell to whole organ global sensitivity analysis on a four-chamber heart electromechanics model using Gaussian processes emulators. PLOS Computational Biology, 19(6):e1011257, June 2023. ISSN 1553-7358. doi:10.1371/journal.pcbi.1011257. URL https://journals.plos.org/ploscompbiol/article?id=10.1371/journal.pcbi.1011257. Publisher: Public Library of Science. PMC10328347 37363928

[48] Lazarus Alan, Dalton David, Husmeier Dirk, and Gao Hao. Sensitivity analysis and inverse uncertainty quantification for the left ventricular passive mechanics. Biomechanics and Modeling in Mechanobiology, 21(3):953–982, June 2022. ISSN 1617-7940. doi:10.1007/s10237-022-01571-8. URL https://link.springer.com/article/10.1007/s10237-022-01571-8. Company: Springer Distributor: Springer Institution: Springer Label: Springer Number: 3 Publisher: Springer Berlin Heidelberg. 35377030 PMC9132878

[49] Menon Karthik, Zanoni Andrea, Khan M. Owais, Geraci Gianluca, Nieman Koen, Schiavazzi Daniele E., and Marsden Alison L.. Personalized and uncertainty-aware coronary hemodynamics simulations: From Bayesian estimation to improved multi-fidelity uncertainty quantification. Computer Methods and Programs in Biomedicine, 271:108951, November 2025. ISSN 0169-2607. doi:10.1016/j.cmpb.2025.108951. URL https://www.sciencedirect.com/science/article/pii/S0169260725003682. PMC13016506 40737995

[50] Lee John D., Richter Jakob, Pfaller Martin R., Szafron Jason M., Menon Karthik, Zanoni Andrea, Ma Michael R., Feinstein Jeffrey A., Kreutzer Jacqueline, Marsden Alison L., and Schiavazzi Daniele E.. A probabilistic neural twin for treatment planning in peripheral pulmonary artery stenosis. International Journal for Numerical Methods in Biomedical Engineering, 40(5):e3820, 2024. ISSN 2040-7947. doi:10.1002/cnm.3820. URL https://onlinelibrary.wiley.com/doi/abs/10.1002/cnm.3820. _eprint: https://onlinelibrary.wiley.com/doi/pdf/10.1002/cnm.3820. 38544354 PMC11131421

[51] Torre Michele, Morganti Simone, Nitti Alessandro, de Tullio Marco D., Pasqualini Francesco S., and Reali Alessandro. Isogeometric mixed collocation of nearly-incompressible electromechanics in finite deformations for cardiac muscle simulations. Computer Methods in Applied Mechanics and Engineering, 411:116055, June 2023. ISSN 0045-7825. doi:10.1016/j.cma.2023.116055. URL https://www.sciencedirect.com/science/article/pii/S0045782523001792.

[52] Lluch Èric, Camara Oscar, Doste Rubén, Bijnens Bart, Mathieu De Craene Maxime Sermesant, Wang Vicky Y., Nash Martyn P., and Morales Hernán G.. Calibration of a fully coupled electromechanical meshless computational model of the heart with experimental data. Computer Methods in Applied Mechanics and Engineering, 364: 112869, June 2020. ISSN 0045-7825. doi:10.1016/j.cma.2020.112869. URL https://www.sciencedirect.com/science/article/pii/S0045782520300505.

[53] Aróstica Reidmen, Nolte David, Brown Aaron, Gebauer Amadeus, Karabelas Elias, Jilberto Javiera, Salvador Matteo, Bucelli Michele, Piersanti Roberto, Osouli Kasra, Augustin Christoph, Finsberg Henrik, Shi Lei, Hirschvogel Marc, Pfaller Martin, Africa Pasquale Claudio, Gsell Matthias, Marsden Alison, Nordsletten David, Regazzoni Francesco, Plank Gernot, Sundnes Joakim, Dede’ Luca, Peirlinck Mathias, Vedula Vijay, Wall Wolfgang, and Bertoglio Cristóbal. A software benchmark for cardiac elastodynamics. Computer Methods in Applied Mechanics and Engineering, 435:117485, February 2025. ISSN 0045-7825. doi:10.1016/j.cma.2024.117485. URL https://www.sciencedirect.com/science/article/pii/S0045782524007394.

[54] Avazmohammadi Reza, Soares João S., Li David S., Raut Samarth S., Gorman Robert C., and Sacks Michael S.. A Contemporary Look at Biomechanical Models of Myocardium. Annual Review of Biomedical Engineering, 21(Volume 21, 2019):417–442, June 2019. ISSN 1523-9829, 1545–4274. doi:10.1146/annurev-bioeng-062117-121129. URL https://www.annualreviews.org/content/journals/10.1146/annurev-bioeng-062117-121129. Publisher: Annual Reviews. PMC6626320 31167105

[55] Niederer Steven A., Campbell Kenneth S., and Campbell Stuart G.. A short history of the development of mathematical models of cardiac mechanics. Journal of Molecular and Cellular Cardiology, 127:11–19, February 2019. ISSN 0022-2828. doi:10.1016/j.yjmcc.2018.11.015. URL https://www.sciencedirect.com/science/article/pii/S0022282818309751. 30503754 PMC6525149

[56] Bracamonte Johane H., Saunders Sarah K., Wilson John S., Truong Uyen T., and Soares Joao S.. Patient-Specific Inverse Modeling of In Vivo Cardiovascular Mechanics with Medical Image-Derived Kinematics as Input Data: Concepts, Methods, and Applications. Applied Sciences, 12(8):3954, January 2022. ISSN 2076-3417. doi:10.3390/app12083954. URL https://www.mdpi.com/2076-3417/12/8/3954. Number: 8 Publisher: Multidisciplinary Digital Publishing Institute. 36911244 PMC10004130

[57] Rodero Cristobal, Baptiste Tiffany M. G., Barrows Rosie K., Lewalle Alexandre, Niederer Steven A., and Strocchi Marina. Advancing clinical translation of cardiac biomechanics models: a comprehensive review, applications and future pathways. Frontiers in Physics, 11, November 2023. ISSN 2296-424X. doi:10.3389/fphy.2023.1306210. URL https://www.frontiersin.org/journals/physics/articles/10.3389/fphy.2023.1306210/full. Publisher: Frontiers. PMC7615748 38500690

[58] Arzani Amirhossein, Wang Jian-Xun, Sacks Michael S., and Shadden Shawn C.. Machine Learning for Cardiovascular Biomechanics Modeling: Challenges and Beyond. Annals of Biomedical Engineering, 50(6): 615–627, June 2022. ISSN 1573-9686. doi:10.1007/s10439-022-02967-4. URL https://doi.org/10.1007/s10439-022-02967-4. 35445297

[59] Lüscher Thomas F. What is a normal blood pressure? European Heart Journal, 39(24):2233–2240, June 2018. ISSN 0195-668X. doi:10.1093/eurheartj/ehy330. URL https://doi.org/10.1093/eurheartj/ehy330. 29893817

[60] Mebazaa Alexandre, Karpati Peter, Renaud Estelle, and Algotsson Lars. Acute right ventricular failure—from pathophysiology to new treatments. Intensive Care Medicine, 30(2):185–196, February 2004. ISSN 1432-1238. doi:10.1007/s00134-003-2025-3. URL https://link-springer-com.laneproxy.stanford.edu/article/10.1007/s00134-003-2025-3. Company: Springer Distributor: Springer Institution: Springer Label: Springer Number: 2 Publisher: Springer Berlin Heidelberg. 14618229

[61] Matsukubo Haruo, Matsuura Tokru, Endo Naoto, Asayama Jun, Watanabe Toshimitsu, Furukawa Keizo, Kunishige Hiroshi, Katsume Hiroshi, and Ijichi Hamao. Echocardiographic measurement of right ventricular wall thickness. A new application of subxiphoid echocardiography., August 1977. URL https://www.ahajournals.org/doi/epdf/10.1161/01.CIR.56.2.278. 10.1161/01.cir.56.2.278 872322

[62] Fedele Marco, Piersanti Roberto, Regazzoni Francesco, Salvador Matteo, Africa Pasquale Claudio, Bucelli Michele, Zingaro Alberto, Dede’ Luca, and Quarteroni Alfio. A comprehensive and biophysically detailed computational model of the whole human heart electromechanics. Computer Methods in Applied Mechanics and Engineering, 410:115983, May 2023. ISSN 0045-7825. doi:10.1016/j.cma.2023.115983. URL https://www.sciencedirect.com/science/article/pii/S0045782523001068.

[63] Peirlinck M., Sahli Costabal F., Yao J., Guccione J. M., Tripathy S., Wang Y., Ozturk D., Segars P., Morrison T. M., Levine S., and Kuhl E.. Precision medicine in human heart modeling. Biomechanics and Modeling in Mechanobiology, 20(3):803–831, June 2021. ISSN 1617-7940. doi:10.1007/s10237-021-01421-z. URL https://doi.org/10.1007/s10237-021-01421-z. 33580313 PMC8154814

[64] Zygote Media Group Inc. Zygote Solid 3D Heart Generations I & II Development Report. Technical report, 2014.

[65] Baillargeon Brian, Rebelo Nuno, Fox David D., Taylor Robert L., and Kuhl Ellen. The Living Heart Project: A robust and integrative simulator for human heart function. European journal of mechanics. A, Solids, 48:38–47, 2014. ISSN 0997-7538. doi:10.1016/j.euromechsol.2014.04.001. URL https://www.ncbi.nlm.nih.gov/pmc/articles/PMC4175454/. 25267880 PMC4175454

[66] Santiago Alfonso, Santiago Alfonso, Aguado-Sierra Jazmín, Aguado-Sierra Jazmin, Zavala-Aké Miguel, Zavala-Aké Miguel, Doste Ruben, Doste Rubén, Doste-Beltran Ruben, Gómez Samuel Samuel, Arís Ruth, Arís Ruth, Cajas Juan Carlos, Cajas J.C., Casoni Eva, Casoni Eva, Vázquez Mariano, and Vázquez Mariano. Fully coupled fluid-electro-mechanical model of the human heart for supercomputers. International Journal for Numerical Methods in Biomedical Engineering, 34(12), December 2018. doi:10.1002/cnm.3140. MAG ID: 2885942414. 30117302

[67] Strocchi Marina, Augustin Christoph M., Gsell Matthias A. F., Karabelas Elias, Neic Aurel, Gillette Karli, Razeghi Orod, Prassl Anton J., Vigmond Edward J., Behar Jonathan M., Gould Justin, Sidhu Baldeep, Rinaldi Christopher A., Bishop Martin J., Plank Gernot, and Niederer Steven A.. A publicly available virtual cohort of four-chamber heart meshes for cardiac electro-mechanics simulations. PLOS ONE, 15(6):e0235145, June 2020. ISSN 1932-6203. doi:10.1371/journal.pone.0235145. URL https://journals.plos.org/plosone/article?id=10.1371/journal.pone.0235145. Publisher: Public Library of Science. PMC7319311 32589679

[68] Arts Theo, Delhaas Tammo, Bovendeerd Peter, Verbeek Xander, and Prinzen Frits W.. Adaptation to mechanical load determines shape and properties of heart and circulation: the CircAdapt model. American Journal of Physiology-Heart and Circulatory Physiology, 288(4):H1943–H1954, April 2005. ISSN 0363-6135. doi:10.1152/ajpheart.00444.2004. URL https://journals.physiology.org/doi/full/10.1152/ajpheart.00444.2004. Publisher: American Physiological Society. 15550528

[69] Strocchi Marina, Rodero Cristobal, Roney Caroline H., Costa Caroline Mendonca, Plank Gernot, Lamata Pablo, and Niederer Steven A.. A Semi-automatic Pipeline for Generation of Large Cohorts of Four-Chamber Heart Meshes. In Regnier Michael and Childers Matthew, editors, Familial Cardiomyopathies: Methods and Protocols, pages 117–127. Springer US, New York, NY, 2024. ISBN 978-1-07-163527-8. doi:10.1007/978-1-0716-3527-8_7. URL https://doi.org/10.1007/978-1-0716-3527-8_7. 38038846

[70] Si Hang. TetGen, a Delaunay-Based Quality Tetrahedral Mesh Generator. ACM Trans. Math. Softw., 41(2): 11:1–11:36, February 2015. ISSN 0098-3500. doi:10.1145/2629697. URL https://dl.acm.org/doi/10.1145/2629697.

[71] {The CGAL Project}. {CGAL} User and Reference Manual. {CGAL Editorial Board}, {6.0.1} edition, 2024. URL https://doc.cgal.org/6.0.1/Manual/packages.html.

[72] Geuzaine Christophe and Remacle Jean-François. Gmsh: A 3-D finite element mesh generator with built-in pre- and post-processing facilities. International Journal for Numerical Methods in Engineering, 79(11):1309–1331, 2009. ISSN 1097-0207. doi:10.1002/nme.2579. URL https://onlinelibrary.wiley.com/doi/abs/10.1002/nme.2579. _eprint: https://onlinelibrary.wiley.com/doi/pdf/10.1002/nme.2579.

[73] Neic Aurel, Gsell Matthias A. F., Karabelas Elias, Prassl Anton J., and Plank Gernot. Automating image-based mesh generation and manipulation tasks in cardiac modeling workflows using Meshtool. SoftwareX, 11:100454, January 2020. ISSN 2352-7110. doi:10.1016/j.softx.2020.100454. URL https://www.sciencedirect.com/science/article/pii/S235271101930295X. PMC7326605 32607406

[74] Pak Daniel H., Liu Minliang, Kim Theodore, Liang Liang, Caballero Andres, Onofrey John, Ahn Shawn S., Xu Yilin, McKay Raymond, Sun Wei, Gleason Rudolph, and Duncan James S.. Patient-Specific Heart Geometry Modeling for Solid Biomechanics Using Deep Learning. IEEE Transactions on Medical Imaging, 43(1):203–215, January 2024. ISSN 1558-254X. doi:10.1109/TMI.2023.3294128. URL https://ieeexplore.ieee.org/abstract/document/10178071. Conference Name: IEEE Transactions on Medical Imaging. 37432807 PMC10764002

[75] Zheng Yefeng, Barbu Adrian, Georgescu Bogdan, Scheuering Michael, and Comaniciu Dorin. Four-Chamber Heart Modeling and Automatic Segmentation for 3-D Cardiac CT Volumes Using Marginal Space Learning and Steerable Features. IEEE Transactions on Medical Imaging, 27(11):1668–1681, November 2008. ISSN 1558-254X. doi:10.1109/TMI.2008.2004421. URL https://ieeexplore.ieee.org/document/4601463. 18955181

[76] Romaszko Lukasz, Borowska Agnieszka, Lazarus Alan, Dalton David, Berry Colin, Luo Xiaoyu, Husmeier Dirk, and Gao Hao. Neural network-based left ventricle geometry prediction from CMR images with application in biomechanics. Artificial Intelligence in Medicine, 119:102140, September 2021. ISSN 0933-3657. doi:10.1016/j.artmed.2021.102140. URL https://www.sciencedirect.com/science/article/pii/S0933365721001330. 34531009

[77] Kong Fanwei and Shadden Shawn C.. Learning Whole Heart Mesh Generation From Patient Images for Computational Simulations. IEEE Transactions on Medical Imaging, 42(2):533–545, February 2023. ISSN 1558-254X. doi:10.1109/TMI.2022.3219284. URL https://ieeexplore.ieee.org/abstract/document/9936657. Conference Name: IEEE Transactions on Medical Imaging. 36327186

[78] Narayanan Arjun, Kong Fanwei, and Shadden Shawn. LinFlo-Net: A Two-Stage Deep Learning Method to Generate Simulation Ready Meshes of the Heart. Journal of Biomechanical Engineering, 146(071005), March 2024. ISSN 0148-0731. doi:10.1115/1.4064527. URL https://doi.org/10.1115/1.4064527. 38258957

[79] Kong Fanwei, Stocker Sascha, Choi Perry S., Ma Michael, Ennis Daniel B., and Marsden Alison L.. SDF4CHD: Generative modeling of cardiac anatomies with congenital heart defects. Medical Image Analysis, 97:103293, October 2024. ISSN 1361-8415. doi:10.1016/j.media.2024.103293. URL https://www.sciencedirect.com/science/article/pii/S1361841524002184. PMC11372630 39146700

[80] Hadjicharalambous Myrianthi, Stoeck Christian T., Weisskopf Miriam, Cesarovic Nikola, Ioannou Eleftherios, Vavourakis Vasileios, and Nordsletten David A.. Investigating the reference domain influence in personalised models of cardiac mechanics. Biomechanics and Modeling in Mechanobiology, 20(4):1579–1597, August 2021. ISSN 1617-7940. doi:10.1007/s10237-021-01464-2. URL https://doi.org/10.1007/s10237-021-01464-2. 34047891

[81] Strocchi Marina, Gsell Matthias A. F., Augustin Christoph M., Razeghi Orod, Roney Caroline H., Prassl Anton J., Vigmond Edward J., Behar Jonathan M., Gould Justin S., Rinaldi Christopher A., Bishop Martin J., Plank Gernot, and Niederer Steven A.. Simulating ventricular systolic motion in a four-chamber heart model with spatially varying robin boundary conditions to model the effect of the pericardium. Journal of Biomechanics, 101:109645, March 2020. ISSN 0021-9290. doi:10.1016/j.jbiomech.2020.109645. URL https://www.sciencedirect.com/science/article/pii/S002192902030052X. PMC7677892 32014305

[82] Wang Vicky Y., Lam H. I., Ennis Daniel B., Cowan Brett R., Young Alistair A., and Nash Martyn P.. Modelling passive diastolic mechanics with quantitative MRI of cardiac structure and function. Medical Image Analysis, 13(5):773–784, October 2009. ISSN 1361-8415. doi:10.1016/j.media.2009.07.006. URL https://www.sciencedirect.com/science/article/pii/S1361841509000619. 19664952 PMC6467494

[83] Augustin Christoph M., Gsell Matthias A. F., Karabelas Elias, Willemen Erik, Prinzen Frits W., Lumens Joost, Vigmond Edward J., and Plank Gernot. A computationally efficient physiologically comprehensive 3D–0D closed-loop model of the heart and circulation. Computer Methods in Applied Mechanics and Engineering, 386:114092, December 2021. ISSN 0045-7825. doi:10.1016/j.cma.2021.114092. URL https://www.sciencedirect.com/science/article/pii/S0045782521004230. PMC7611781 34630765

[84] Barnafi N. A., Regazzoni F., and Riccobelli D.. Reconstructing relaxed configurations in elastic bodies: Mathematical formulations and numerical methods for cardiac modeling. Computer Methods in Applied Mechanics and Engineering, 423:116845, April 2024. ISSN 0045-7825. doi:10.1016/j.cma.2024.116845. URL https://www.sciencedirect.com/science/article/pii/S0045782524001014.

[85] Hirschvogel Marc, Bassilious Marina, Jagschies Lasse, Wildhirt Stephen M., and Gee Michael W.. A monolithic 3D-0D coupled closed-loop model of the heart and the vascular system: Experiment-based parameter estimation for patient-specific cardiac mechanics. International Journal for Numerical Methods in Biomedical Engineering, 33(8):e2842, 2017. ISSN 2040-7947. doi:10.1002/cnm.2842. URL https://onlinelibrary.wiley.com/doi/abs/10.1002/cnm.2842. _eprint: https://onlinelibrary.wiley.com/doi/pdf/10.1002/cnm.2842. 27743468

[86] Holzapfel Gerhard A. and Ogden Ray W.. Constitutive Modelling of Passive Myocardium: A Structurally Based Framework for Material Characterization. Philosophical Transactions: Mathematical, Physical and Engineering Sciences, 367(1902):3445–3475, 2009. ISSN 1364-503X. URL https://www.jstor.org/stable/40485676. Publisher: Royal Society. 10.1098/rsta.2009.0091 19657007

[87] Eriksson TSE, Prassl AJ, Plank G, and Holzapfel GA. Influence of myocardial fiber/sheet orientations on left ventricular mechanical contraction. Mathematics and Mechanics of Solids, 18(6):592–606, August 2013. ISSN 1081-2865. doi:10.1177/1081286513485779. URL https://doi.org/10.1177/1081286513485779. Publisher: SAGE Publications Ltd STM.

[88] Holz David, Martonová Denisa, Schaller Emely, Duong Minh Tuan, Alkassar Muhannad, Weyand Michael, and Leyendecker Sigrid. Transmural fibre orientations based on Laplace–Dirichlet-Rule-Based-Methods and their influence on human heart simulations. Journal of Biomechanics, 156:111643, July 2023. ISSN 0021-9290. doi:10.1016/j.jbiomech.2023.111643. URL https://www.sciencedirect.com/science/article/pii/S0021929023002129. 37321157

[89] Rodríguez-Padilla Jairo, Petras Argyrios, Magat Julie, Bayer Jason, Bihan-Poudec Yann, El Hamrani Dounia, Ramlugun Girish, Neic Aurel, Augustin Christoph M., Vaillant Fanny, Constantin Marion, Benoist David, Pourtau Line, Dubes Virginie, Rogier Julien, Labrousse Louis, Bernus Olivier, Quesson Bruno, Haïssaguerre Michel, Gsell Matthias, Plank Gernot, Ozenne Valéry, and Vigmond Edward. Impact of intraventricular septal fiber orientation on cardiac electromechanical function. American Journal of Physiology-Heart and Circulatory Physiology, 322(6):H936–H952, June 2022. ISSN 0363-6135. doi:10.1152/ajpheart.00050.2022. URL https://journals.physiology.org/doi/full/10.1152/ajpheart.00050.2022. Publisher: American Physiological Society. 35302879 PMC9109800

[90] Lombaert Herve, Peyrat Jean-Marc, Croisille Pierre, Rapacchi Stanislas, Fanton Laurent, Cheriet Farida, Clarysse Patrick, Magnin Isabelle, Delingette Hervé, and Ayache Nicholas. Human Atlas of the Cardiac Fiber Architecture: Study on a Healthy Population. IEEE Transactions on Medical Imaging, 31(7):1436–1447, July 2012. ISSN 1558-254X. doi:10.1109/TMI.2012.2192743. URL https://ieeexplore.ieee.org/document/6177265. 22481815

[91] Anderson Robert H., Smerup Morten, Sanchez-Quintana Damian, Loukas Marios, and Lunkenheimer Paul P.. The three-dimensional arrangement of the myocytes in the ventricular walls. Clinical Anatomy, 22(1):64–76, 2009. ISSN 1098-2353. doi:10.1002/ca.20645. URL https://onlinelibrary.wiley.com/doi/abs/10.1002/ca.20645. _eprint: https://onlinelibrary.wiley.com/doi/pdf/10.1002/ca.20645. 18567009

[92] Agger Peter and Stephenson Robert S.. Assessing Myocardial Architecture: The Challenges and Controversies. Journal of Cardiovascular Development and Disease, 7(4):47, December 2020. ISSN 2308-3425. doi:10.3390/jcdd7040047. URL https://www.mdpi.com/2308-3425/7/4/47. Number: 4 Publisher: Multidisciplinary Digital Publishing Institute. 33137874 PMC7711767

[93] Legrice I. J., Hunter P. J., and Smaill B. H.. Laminar structure of the heart: a mathematical model. American Journal of Physiology-Heart and Circulatory Physiology, 272(5):H2466–H2476, May 1997. ISSN 0363-6135. doi:10.1152/ajpheart.1997.272.5.H2466. URL https://journals.physiology.org/doi/abs/10.1152/ajpheart.1997.272.5.H2466. Publisher: American Physiological Society. 9176318

[94] Sommer Gerhard, Schriefl Andreas J., Andrä Michaela, Sacherer Michael, Viertler Christian, Wolinski Heimo, and Holzapfel Gerhard A.. Biomechanical properties and microstructure of human ventricular myocardium. Acta Biomaterialia, 24:172–192, September 2015. ISSN 1742-7061. doi:10.1016/j.actbio.2015.06.031. URL https://www.sciencedirect.com/science/article/pii/S1742706115300039. 26141152

[95] Pashakhanloo Farhad, Herzka Daniel A., Ashikaga Hiroshi, Mori Susumu, Gai Neville, Bluemke David A., Trayanova Natalia A., and McVeigh Elliot R.. Myofiber Architecture of the Human Atria as Revealed by Submillimeter Diffusion Tensor Imaging. Circulation: Arrhythmia and Electrophysiology, 9(4):e004133, April 2016. doi:10.1161/CIRCEP.116.004133. URL https://www.ahajournals.org/doi/10.1161/CIRCEP.116.004133. Publisher: American Heart Association. PMC7035884 27071829

[96] Bayer J. D., Blake R. C., Plank G., and Trayanova N. A.. A novel rule-based algorithm for assigning myocardial fiber orientation to computational heart models. Annals of Biomedical Engineering, 40(10):2243–2254, October 2012. ISSN 1573-9686. doi:10.1007/s10439-012-0593-5. 22648575 PMC3518842

[97] Bayer Jason, Prassl Anton J., Pashaei Ali, Gomez Juan F., Frontera Antonio, Neic Aurel, Plank Gernot, and Vigmond Edward J.. Universal ventricular coordinates: A generic framework for describing position within the heart and transferring data. Medical Image Analysis, 45:83–93, April 2018. ISSN 13618415. doi:10.1016/j.media.2018.01.005. URL https://linkinghub.elsevier.com/retrieve/pii/S1361841518300203. 29414438

[98] Doste R., Soto-Iglesias D., Bernardino G., Alcaine A., Sebastian R., Giffard-Roisin S., Sermesant M., Berruezo A., Sanchez-Quintana D., and Camara O.. A rule-based method to model myocardial fiber orientation in cardiac biventricular geometries with outflow tracts. International Journal for Numerical Methods in Biomedical Engineering, 35(4), 2019. doi:10.1002/cnm.3185. 30721579

[99] Holz David, Duong Minh Tuan, Msartonová Denisa, Alkassar Muhannad, and Leyendecker Sigrid. A Transmural Path Model Improves the Definition of the Orthotropic Tissue Structure in Heart Simulations. Journal of Biomechanical Engineering, 144(031002), October 2021. ISSN 0148-0731. doi:10.1115/1.4052219. URL https://doi.org/10.1115/1.4052219. 34423814

[100] Bovendeerd Peter H. M., Kroon Wilco, and Delhaas Tammo. Determinants of left ventricular shear strain. American Journal of Physiology-Heart and Circulatory Physiology, 297(3):H1058–H1068, September 2009. ISSN 0363-6135. doi:10.1152/ajpheart.01334.2008. URL https://journals.physiology.org/doi/full/10.1152/ajpheart.01334.2008. Publisher: American Physiological Society. 19592607

[101] Axel Leon, Van Wedeen J, and Ennis Daniel B. Probing dynamic myocardial microstructure with cardiac magnetic resonance diffusion tensor imaging. Journal of Cardiovascular Magnetic Resonance, 16(1):89, December 2014. ISSN 1097-6647. doi:10.1186/s12968-014-0089-6. URL https://www.sciencedirect.com/science/article/pii/S1097664723001564. 25388937 PMC4229597

[102] Kung Geoffrey L., Nguyen Tom C., Itoh Aki, Skare Stefan, Ingels Neil B. Jr, Miller D. Craig, and Ennis Daniel B.. The presence of two local myocardial sheet populations confirmed by diffusion tensor MRI and histological validation. Journal of Magnetic Resonance Imaging, 34(5):1080–1091, 2011. ISSN 1522-2586. doi:10.1002/jmri.22725. URL https://onlinelibrary.wiley.com/doi/abs/10.1002/jmri.22725._eprint: https://onlinelibrary.wiley.com/doi/pdf/10.1002/jmri.22725. 21932362 PMC3195899

[103] Guan Debao, Yao Jiang, Luo Xiaoyu, and Gao Hao. Effect of myofibre architecture on ventricular pump function by using a neonatal porcine heart model: from DT-MRI to rule-based methods. Royal Society Open Science, 7 (4):191655, April 2020. doi:10.1098/rsos.191655. URL https://royalsocietypublishing.org/doi/10.1098/rsos.191655. Publisher: Royal Society. PMC7211874 32431869

[104] Garcia-Canadilla Patricia, Dejea Hector, Bonnin Anne, Balicevic Vedrana, Loncaric Sven, Zhang Chong, Butakoff Constantine, Aguado-Sierra Jazmin, Vázquez Mariano, Jackson Laurence H., Stuckey Daniel J., Rau Cristoph, Stampanoni Marco, Bijnens Bart, and Cook Andrew C.. Complex Congenital Heart Disease Associated With Disordered Myocardial Architecture in a Midtrimester Human Fetus. Circulation: Cardiovascular Imaging, 11(10):e007753, October 2018. doi:10.1161/CIRCIMAGING.118.007753. URL https://www.ahajournals.org/doi/full/10.1161/CIRCIMAGING.118.007753. Publisher: American Heart Association. 30354476

[105] Ghonim Sarah, Voges Inga, Gatehouse Peter D., Keegan Jennifer, Gatzoulis Michael A., Kilner Philip J., and Babu-Narayan Sonya V.. Myocardial Architecture, Mechanics, and Fibrosis in Congenital Heart Disease. Frontiers in Cardiovascular Medicine, 4, May 2017. ISSN 2297-055X. doi:10.3389/fcvm.2017.00030. URL https://www.frontiersin.org/journals/cardiovascular-medicine/articles/10.3389/fcvm.2017.00030/full. Publisher: Frontiers. PMC5440586 28589126

[106] Yen Ho Siew. Anatomy and myoarchitecture of the left ventricular wall in normal and in disease. European Journal of Echocardiography, 10(8):iii3–iii7, December 2009. ISSN 1525-2167. doi:10.1093/ejechocard/jep159. URL https://doi.org/10.1093/ejechocard/jep159. 19889656

[107] Ma Weikang, Gong Henry, Jani Vivek, Lee Kyoung Hwan, Landim-Vieira Maicon, Papadaki Maria, Pinto Jose R., Aslam M. Imran, Cammarato Anthony, and Irving Thomas. Myofibril orientation as a metric for characterizing heart disease. Biophysical Journal, 121(4):565–574, February 2022. ISSN 0006-3495. doi:10.1016/j.bpj.2022.01.009. URL https://www.cell.com/biophysj/abstract/S0006-3495(22)00038-8. Publisher: Elsevier. 35032456 PMC8874025

[108] Planinc Ivo, Garcia-Canadilla Patricia, Dejea Hector, Ilic Ivana, Guasch Eduard, Zamora Monica, Crispi Fàtima, Stampanoni Marco, Milicic Davor, Bijnens Bart, Bonnin Anne, and Cikes Maja. Comprehensive assessment of myocardial remodeling in ischemic heart disease by synchrotron propagation based X-ray phase contrast imaging. Scientific Reports, 11(1):14020, July 2021. ISSN 2045-2322. doi:10.1038/s41598-021-93054-6. URL https://www.nature.com/articles/s41598-021-93054-6. Publisher: Nature Publishing Group. 34234175 PMC8263575

[109] Washio Takumi, Yoneda Kazunori, Okada Jun-ichi, Kariya Taro, Sugiura Seiryo, and Hisada Toshiaki. Ventricular fiber optimization utilizing the branching structure. International Journal for Numerical Methods in Biomedical Engineering, 32(7):e02753, 2016. ISSN 2040-7947. doi:10.1002/cnm.2753. URL https://onlinelibrary.wiley.com/doi/abs/10.1002/cnm.2753. _eprint: https://onlinelibrary.wiley.com/doi/pdf/10.1002/cnm.2753. 26453026

[110] Washio Takumi, Sugiura Seiryo, Okada Jun-ichi, and Hisada Toshiaki. Using Systolic Local Mechanical Load to Predict Fiber Orientation in Ventricles. Frontiers in Physiology, 11, June 2020. ISSN 1664-042X. doi:10.3389/fphys.2020.00467. URL https://www.frontiersin.org/journals/physiology/articles/10.3389/fphys.2020.00467/full. Publisher: Frontiers. PMC7295989 32581822

[111] Toussaint Nicolas, Stoeck Christian T., Schaeffter Tobias, Kozerke Sebastian, Sermesant Maxime, and Batchelor Philip G.. *In vivo* human cardiac fibre architecture estimation using shape-based diffusion tensor processing. Medical Image Analysis, 17(8):1243–1255, December 2013. ISSN 1361-8415. doi:10.1016/j.media.2013.02.008. URL https://www.sciencedirect.com/science/article/pii/S1361841513000224. 23523287

[112] Nielles Vallespin Sonia, Khalique Zohya, Ferreira Pedro F., de Silva Ranil, Scott Andrew D., Kilner Philip, McGill Laura-Ann, Giannakidis Archontis, Gatehouse Peter D., Ennis Daniel, Aliotta Eric, Al Khalil Majid, Kellman Peter, Mazilu Dumitru, Balaban Robert S., Firmin David N., Arai Andrew E., and Pennell Dudley J.. Assessment of Myocardial Microstructural Dynamics by In Vivo Diffusion Tensor Cardiac Magnetic Resonance. Journal of the American College of Cardiology, 69(6):661–676, February 2017. doi:10.1016/j.jacc.2016.11.051. URL https://www.jacc.org/doi/full/10.1016/j.jacc.2016.11.051. Publisher: American College of Cardiology Foundation. 28183509 PMC8672367

[113] Moulin Kévin, Verzhbinsky Ilya A., Maforo Nyasha G., Perotti Luigi E., and Ennis Daniel B.. Probing cardiomyocyte mobility with multi-phase cardiac diffusion tensor MRI. PLOS ONE, 15(11):e0241996, November 2020. ISSN 1932-6203. doi:10.1371/journal.pone.0241996. URL https://journals.plos.org/plosone/article?id=10.1371/journal.pone.0241996. Publisher: Public Library of Science. PMC7660468 33180823

[114] Phipps Kellie, van de Boomen Maaike, Eder Robert, Michelhaugh Sam Allen, Spahillari Aferdita, Kim Joan, Parajuli Shestruma, Reese Timothy G., Mekkaoui Choukri, Das Saumya, Gee Denise, Shah Ravi, Sosnovik David E., and Nguyen Christopher. Accelerated in Vivo Cardiac Diffusion-Tensor MRI Using Residual Deep Learning–based Denoising in Participants with Obesity. Radiology: Cardiothoracic Imaging, 3(3): e200580, June 2021. doi:10.1148/ryct.2021200580. URL https://pubs.rsna.org/doi/full/10.1148/ryct.2021200580. Publisher: Radiological Society of North America. PMC8259662 34250491

[115] Stimm Johanna, Nordsletten David A., Jilberto Javiera, Miller Renee, Berberoğlu Ezgi, Kozerke Sebastian, and Stoeck Christian T.. Personalization of biomechanical simulations of the left ventricle by in-vivo cardiac DTI data: Impact of fiber interpolation methods. Frontiers in Physiology, 13, November 2022. ISSN 1664-042X. doi:10.3389/fphys.2022.1042537. URL https://www.frontiersin.org/journals/physiology/articles/10.3389/fphys.2022.1042537/full. Publisher: Frontiers. PMC9742433 36518106

[116] Tueni Nicole, Allain Jean-Marc, and Genet Martin. On the structural origin of the anisotropy in the myocardium: Multiscale modeling and analysis. Journal of the Mechanical Behavior of Biomedical Materials, 138:105600, February 2023. ISSN 1751-6161. doi:10.1016/j.jmbbm.2022.105600. URL https://www.sciencedirect.com/science/article/pii/S1751616122005057. 36525875

[117] Kakaletsis Sotirios, Meador William D., Mathur Mrudang, Sugerman Gabriella P., Jazwiec Tomasz, Malinowski Marcin, Lejeune Emma, Timek Tomasz A., and Rausch Manuel K.. Right ventricular myocardial mechanics: Multi-modal deformation, microstructure, modeling, and comparison to the left ventricle. Acta Biomaterialia, 123:154–166, March 2021. ISSN 1742-7061. doi:10.1016/j.actbio.2020.12.006. URL https://www.sciencedirect.com/science/article/pii/S174270612030711X. 33338654 PMC7946450

[118] Nemavhola Fulufhelo. Study of biaxial mechanical properties of the passive pig heart: material characterisation and categorisation of regional differences. International Journal of Mechanical and Materials Engineering, 16 (1):6, April 2021. ISSN 2198-2791. doi:10.1186/s40712-021-00128-4. URL https://doi.org/10.1186/s40712-021-00128-4.

[119] Guccione J. M., McCulloch A. D., and Waldman L. K.. Passive Material Properties of Intact Ventricular Myocardium Determined From a Cylindrical Model. Journal of Biomechanical Engineering, 113(1):42–55, February 1991. ISSN 0148-0731. doi:10.1115/1.2894084. URL https://doi.org/10.1115/1.2894084. 2020175

[120] Guccione Julius M., Costa Kevin D., and McCulloch Andrew D.. Finite element stress analysis of left ventricular mechanics in the beating dog heart. Journal of Biomechanics, 28(10):1167–1177, October 1995. ISSN 00219290. doi:10.1016/0021-9290(94)00174-3. URL https://linkinghub.elsevier.com/retrieve/pii/0021929094001743. 8550635

[121] Fröhlich Jonathan, Gerach Tobias, Krauß Jonathan, Loewe Axel, Stengel Laura, and Wieners Christian. Numerical evaluation of elasto-mechanical and visco-elastic electro-mechanical models of the human heart. GAMM-Mitteilungen, 46(3–4):e202370010, 2023. ISSN 1522-2608. doi:10.1002/gamm.202370010. URL https://onlinelibrary.wiley.com/doi/abs/10.1002/gamm.202370010._eprint: https://onlinelibrary.wiley.com/doi/pdf/10.1002/gamm.202370010.

[122] Shavik Sheikh Mohammad, Wall Samuel, Sundnes Joakim, Guccione Julius M., Sengupta Partho, Solomon Scott D., Burkhoff Daniel, and Lee Lik Chuan. Computational Modeling Studies of the Roles of Left Ventricular Geometry, Afterload, and Muscle Contractility on Myocardial Strains in Heart Failure with Preserved Ejection Fraction. Journal of Cardiovascular Translational Research, 14(6):1131–1145, December 2021. ISSN 1937-5395. doi:10.1007/s12265-021-10130-y. URL https://doi.org/10.1007/s12265-021-10130-y. 33928526 PMC9305326

[123] Usyk T.P., Mazhari R., and McCulloch A.D.. Effect of Laminar Orthotropic Myofiber Architecture on Regional Stress and Strain in the Canine Left Ventricle. Journal of elasticity and the physical science of solids, 61(1): 143–164, July 2000. ISSN 1573-2681. doi:10.1023/A:1010883920374. URL https://doi.org/10.1023/A:1010883920374.

[124] Usyk Taras P., LeGrice Ian J., and McCulloch Andrew D.. Computational model of three-dimensional cardiac electromechanics. Computing and Visualization in Science, 4(4):249–257, July 2002. ISSN 1432-9360. doi:10.1007/s00791-002-0081-9. URL https://doi.org/10.1007/s00791-002-0081-9.

[125] Alharbi Yousef. A 3D-0D Computational Model of the Left Ventricle for Investigating Blood Flow Patterns for Cases of Systolic Anterior Motion and after Anterior Mitral Leaflet Splitting. Applied Sciences, 14(1):466, January 2024. ISSN 2076-3417. doi:10.3390/app14010466. URL https://www.mdpi.com/2076-3417/14/1/466. Number: 1 Publisher: Multidisciplinary Digital Publishing Institute.

[126] Eriksson Thomas S. E., Prassl Anton J., Plank Gernot, and Holzapfel Gerhard A.. Modeling the dispersion in electromechanically coupled myocardium. International Journal for Numerical Methods in Biomedical Engineering, 29(11):1267–1284, 2013. ISSN 2040-7947. doi:10.1002/cnm.2575. URL https://onlinelibrary.wiley.com/doi/abs/10.1002/cnm.2575. _eprint: https://onlinelibrary.wiley.com/doi/pdf/10.1002/cnm.2575. 23868817 PMC3970090

[127] Gültekin Osman, Sommer Gerhard, and Holzapfel Gerhard A.. An orthotropic viscoelastic model for the passive myocardium: continuum basis and numerical treatment. Computer Methods in Biomechanics and Biomedical Engineering, 19(15):1647–1664, November 2016. ISSN 1025-5842. doi:10.1080/10255842.2016.1176155. URL https://doi.org/10.1080/10255842.2016.1176155. Publisher: Taylor & Francis _eprint: https://doi.org/10.1080/10255842.2016.1176155. 27146848

[128] Nolan D. R., Gower A. L., Destrade M., Ogden R. W., and McGarry J. P.. A robust anisotropic hyperelastic formulation for the modelling of soft tissue. Journal of the Mechanical Behavior of Biomedical Materials, 39:48–60, November 2014. ISSN 1751-6161. doi:10.1016/j.jmbbm.2014.06.016. URL https://www.sciencedirect.com/science/article/pii/S1751616114001805. 25104546

[129] McEvoy Eoin, Holzapfel Gerhard A., and McGarry Patrick. Compressibility and Anisotropy of the Ventricular Myocardium: Experimental Analysis and Microstructural Modeling. Journal of Biomechanical Engineering, 140(081004), May 2018. ISSN 0148-0731. doi:10.1115/1.4039947. URL https://doi.org/10.1115/1.4039947. 30003247

[130] Vaverka Jiří and Burša Jiří. A modification of Holzapfel–Ogden hyperelastic model of myocardium better describing its passive mechanical behavior. European Journal of Mechanics - A/Solids, 111:105586, May 2025. ISSN 0997-7538. doi:10.1016/j.euromechsol.2025.105586. URL https://www.sciencedirect.com/science/article/pii/S0997753825000208.

[131] Caporizzo Matthew A. and Prosser Benjamin L.. Need for Speed: The Importance of Physiological Strain Rates in Determining Myocardial Stiffness. Frontiers in Physiology, 12:696694, July 2021. doi:10.3389/fphys.2021.696694. URL https://pmc.ncbi.nlm.nih.gov/articles/PMC8361601/. PMC8361601 34393820

[132] Wang Zhijie, Golob Mark J., Chesler Naomi C., Wang Zhijie, Golob Mark J., and Chesler Naomi C.. Viscoelastic Properties of Cardiovascular Tissues. In Viscoelastic and Viscoplastic Materials. IntechOpen, September 2016. ISBN 978-953-51-2603-4. doi:10.5772/64169. URL https://www.intechopen.com/chapters/51650.

[133] Nordsletten David, Capilnasiu Adela, Zhang Will, Wittgenstein Anna, Hadjicharalambous Myrianthi, Sommer Gerhard, Sinkus Ralph, and Holzapfel Gerhard A.. A viscoelastic model for human myocardium. Acta Biomaterialia, 135:441–457, November 2021. ISSN 1742-7061. doi:10.1016/j.actbio.2021.08.036. URL https://www.sciencedirect.com/science/article/pii/S1742706121005699. 34487858

[134] Tikenoğulları Oğuz Ziya, Costabal Francisco Sahli, Yao Jiang, Marsden Alison, and Kuhl Ellen. How viscous is the beating heart? Insights from a computational study. Computational Mechanics, 70(3):565–579, September 2022. ISSN 1432-0924. doi:10.1007/s00466-022-02180-z. URL https://doi.org/10.1007/s00466-022-02180-z. 37274842 PMC10237084

[135] Zhang Will, Capilnasiu Adela, and Nordsletten David. Comparative Analysis of Nonlinear Viscoelastic Models Across Common Biomechanical Experiments. Journal of Elasticity, 145(1):117–152, August 2021. ISSN 1573-2681. doi:10.1007/s10659-021-09827-7. URL https://doi.org/10.1007/s10659-021-09827-7.

[136] Zhang Will, Jilberto Javiera, Sommer Gerhard, Sacks Michael S., Holzapfel Gerhard A., and Nordsletten David A.. Simulating hyperelasticity and fractional viscoelasticity in the human heart. Computer Methods in Applied Mechanics and Engineering, 411:116048, June 2023. ISSN 0045-7825. doi:10.1016/j.cma.2023.116048. URL https://www.sciencedirect.com/science/article/pii/S004578252300172X.

[137] Zhang Will, Capilnasiu Adela, Sommer Gerhard, Holzapfel Gerhard A., and Nordsletten David A.. An efficient and accurate method for modeling nonlinear fractional viscoelastic biomaterials. Computer Methods in Applied Mechanics and Engineering, 362:112834, April 2020. ISSN 0045-7825. doi:10.1016/j.cma.2020.112834. URL https://www.sciencedirect.com/science/article/pii/S0045782520300153. PMC7610983 34136022

[138] Dominique Chapelle P. Le Tallec, Moireau P., and Sorine M.. ENERGY-PRESERVING MUSCLE TISSUE MODEL: FORMULATION AND COMPATIBLE DISCRETIZATIONS. International Journal for Multiscale Computational Engineering, 10(2):189–211, 2012. ISSN 1543-1649. doi:10.1615/IntJMultCompEng.2011002360. URL http://www.dl.begellhouse.com/journals/61fd1b191cf7e96f,42f1beba0b405e70,2491c81155ff42d6.html.

[139] Sugiura Seiryo, Okada Jun-Ichi, Washio Takumi, and Hisada Toshiaki. UT-Heart: A Finite Element Model Designed for the Multiscale and Multiphysics Integration of our Knowledge on the Human Heart. In Cortassa Sonia and Aon Miguel A., editors, Computational Systems Biology in Medicine and Biotechnology: Methods and Protocols, pages 221–245. Springer US, New York, NY, 2022. ISBN 978-1-07-161831-8. doi:10.1007/978-1-0716-1831-8_10. URL https://doi.org/10.1007/978-1-0716-1831-8_10. 35604559

[140] Lafortune Pierre, Arís Ruth, Vázquez Mariano, and Houzeaux Guillaume. Coupled electromechanical model of the heart: Parallel finite element formulation. International Journal for Numerical Methods in Biomedical Engineering, 28(1):72–86, 2012. ISSN 2040-7947. doi:10.1002/cnm.1494. URL https://onlinelibrary.wiley.com/doi/abs/10.1002/cnm.1494. _eprint: https://onlinelibrary.wiley.com/doi/pdf/10.1002/cnm.1494. 25830206

[141] Yin F. C., Chan C. C., and Judd R. M.. Compressibility of perfused passive myocardium. American Journal of Physiology-Heart and Circulatory Physiology, 271(5):H1864–H1870, November 1996. ISSN 0363-6135. doi:10.1152/ajpheart.1996.271.5.H1864. URL https://journals.physiology.org/doi/abs/10.1152/ajpheart.1996.271.5.h1864. Publisher: American Physiological Society. 8945902

[142] Avazmohammadi Reza, Li David S., Leahy Thomas, Shih Elizabeth, Soares João S., Gorman Joseph H., Gorman Robert C., and Sacks Michael S.. An Integrated Inverse Model-Experimental Approach to Determine Soft Tissue Three-Dimensional Constitutive Properties: Application to Post-Infarcted Myocardium. Biomechanics and modeling in mechanobiology, 17(1):31, August 2017. doi:10.1007/s10237-017-0943-1. URL https://pmc.ncbi.nlm.nih.gov/articles/PMC5809201/. 28861630 PMC5809201

[143] Tsuiki Kai and Ritman Erik L.. Direct Evidence that Left Ventricular Myocardium Is Incompressible Throughout Systole and Diastole. The Tohoku Journal of Experimental Medicine, 132(1):119–120, 1980. ISSN 0040-8727, 1349–3329. doi:10.1620/tjem.132.119. URL http://www.jstage.jst.go.jp/article/tjem1920/132/1/132_1_119/_article. 7209964

[144] Vossoughi Jafar, Vaishnav Ramesh N., and Patel Dali J.. Compressibility of the myocardial tissue. Advances in bioengineering, pages 45–48, 1980.

[145] Rodriguez Ignacio, Ennis Daniel B., and Wen Han. Noninvasive measurement of myocardial tissue volume change during systolic contraction and diastolic relaxation in the canine left ventricle. Magnetic Resonance in Medicine, 55(3):484–490, 2006. ISSN 1522-2594. doi:10.1002/mrm.20786. URL https://onlinelibrary.wiley.com/doi/abs/10.1002/mrm.20786. _eprint: https://onlinelibrary.wiley.com/doi/pdf/10.1002/mrm.20786. 16408273 PMC2887312

[146] Bistoquet A, Oshinski J, and Skrinjar O. Myocardial deformation recovery from cine MRI using a nearly incompressible biventricular model. Medical Image Analysis, 12(1):69–85, February 2008. ISSN 13618415. doi:10.1016/j.media.2007.10.009. URL https://linkinghub.elsevier.com/retrieve/pii/S1361841507001090. 18234539

[147] Ashikaga Hiroshi, Coppola Benjamin A., Yamazaki Katrina G., Villarreal Francisco J., Omens Jeffrey H., and Covell James W.. Changes in regional myocardial volume during the cardiac cycle: implications for transmural blood flow and cardiac structure. American Journal of Physiology-Heart and Circulatory Physiology, 295(2): H610–H618, August 2008. ISSN 0363-6135. doi:10.1152/ajpheart.00107.2008. URL https://journals.physiology.org/doi/full/10.1152/ajpheart.00107.2008. Publisher: American Physiological Society. 18515651 PMC2519196

[148] Soares Joao S., Li David S., Lai Eric, Gorman Joseph H. III, Gorman Robert C., and Sacks Michael S.. Modeling of Myocardium Compressibility and its Impact in Computational Simulations of the Healthy and Infarcted Heart. In Pop Mihaela and Wright Graham A, editors, Functional Imaging and Modelling of the Heart, pages 493–501, Cham, 2017. Springer International Publishing. ISBN 978-3-319-59448-4. doi:10.1007/978-3-319-59448-4_47. PMC6510496 31080965

[149] Avazmohammadi Reza, Soares Joao S., Li David S., Eperjesi Thomas, Pilla James, Gorman Robert C., and Sacks Michael S.. On the in vivo systolic compressibility of left ventricular free wall myocardium in the normal and infarcted heart. Journal of Biomechanics, 107:109767, June 2020. ISSN 0021-9290. doi:10.1016/j.jbiomech.2020.109767. URL https://www.sciencedirect.com/science/article/pii/S0021929020301834. PMC7433024 32386714

[150] Liu Hao, Soares João S., Walmsley John, Li David S., Raut Samarth, Avazmohammadi Reza, Iaizzo Paul, Palmer Mark, Gorman Joseph H., Gorman Robert C., and Sacks Michael S.. The impact of myocardial compressibility on organ-level simulations of the normal and infarcted heart. Scientific Reports, 11(1):13466, June 2021. ISSN 2045-2322. doi:10.1038/s41598-021-92810-y. URL https://www.nature.com/articles/s41598-021-92810-y. Publisher: Nature Publishing Group. 34188138 PMC8242073

[151] Kumar Vinayak, Ryu Alexander J., Manduca Armando, Rao Chaitanya, Gibbons Raymond J., Gersh Bernard J., Chandrasekaran Krishnaswamy, Asirvatham Samuel J., Araoz Philip A., Oh Jae K., Egbe Alexander C., Behfar Atta, Borlaug Barry A., and Anavekar Nandan S.. Cardiac MRI demonstrates compressibility in healthy myocardium but not in myocardium with reduced ejection fraction. International Journal of Cardiology, 322:278–283, January 2021. ISSN 0167-5273. doi:10.1016/j.ijcard.2020.08.087. URL https://www.sciencedirect.com/science/article/pii/S0167527320337141. 32871188

[152] Karlon William J., McCulloch Andrew D., Covell James W., Hunter John J., and Omens Jeffrey H.. Regional dysfunction correlates with myofiber disarray in transgenic mice with ventricular expression ofras. American Journal of Physiology-Heart and Circulatory Physiology, 278(3):H898–H906, March 2000. ISSN 0363-6135. doi:10.1152/ajpheart.2000.278.3.H898. URL https://journals.physiology.org/doi/full/10.1152/ajpheart.2000.278.3.H898. Publisher: American Physiological Society. 10710359 PMC3328188

[153] Schriefl Andreas J., Reinisch Andreas J., Sankaran Sethuraman, Pierce David M., and Holzapfel Gerhard A.. Quantitative assessment of collagen fibre orientations from two-dimensional images of soft biological tissues. Journal of the Royal Society Interface, 9(76):3081–3093, November 2012. ISSN 1742-5689. doi:10.1098/rsif.2012.0339. URL https://www.ncbi.nlm.nih.gov/pmc/articles/PMC3479916/. 22764133 PMC3479916

[154] Schriefl Andreas J., Wolinski Heimo, Regitnig Peter, Kohlwein Sepp D., and Holzapfel Gerhard A.. An automated approach for three-dimensional quantification of fibrillar structures in optically cleared soft biological tissues. Journal of The Royal Society Interface, 10(80):20120760, March 2013. doi:10.1098/rsif.2012.0760. URL https://royalsocietypublishing.org/doi/10.1098/rsif.2012.0760. Publisher: Royal Society. PMC3565728 23269845

[155] Ahmad F., Soe S., White N., Johnston R., Khan I., Liao J., Jones M., Prabhu R., Maconochie I., and Theobald Peter. Region-Specific Microstructure in the Neonatal Ventricles of a Porcine Model. Annals of Biomedical Engineering, 46(12):2162–2176, December 2018. ISSN 1573-9686. doi:10.1007/s10439-018-2089-4. 30014287 PMC6267669

[156] Lanir Yoram. Multi-scale Structural Modeling of Soft Tissues Mechanics and Mechanobiology. Journal of Elasticity, 129(1):7–48, December 2017. ISSN 1573-2681. doi:10.1007/s10659-016-9607-0. URL https://doi.org/10.1007/s10659-016-9607-0.

[157] Christian Gasser T., Ogden Ray W, and Holzapfel Gerhard A. Hyperelastic modelling of arterial layers with distributed collagen fibre orientations. Journal of The Royal Society Interface, 3(6):15–35, September 2005. doi:10.1098/rsif.2005.0073. URL https://royalsocietypublishing.org/doi/10.1098/rsif.2005.0073. Publisher: Royal Society. PMC1618483 16849214

[158] Mojumder Joy, Fan Lei, Nguyen Thuy, Campbell Kenneth S., Wenk Jonathan F., Guccione Julius M., Abraham Theodore, and Lee Lik Chuan. Computational analysis of ventricular mechanics in hypertrophic cardiomyopathy patients. Scientific Reports, 13(1):958, January 2023. ISSN 2045-2322. doi:10.1038/s41598-023-28037-w. URL https://www.nature.com/articles/s41598-023-28037-w. Publisher: Nature Publishing Group. 36653468 PMC9849405

[159] Melnik Andrey V., Luo Xiaoyu, and Ogden Ray W.. A generalised structure tensor model for the mixed invariant $I_{8}$. International Journal of Non-Linear Mechanics, 107:137–148, December 2018. ISSN 0020-7462. doi:10.1016/j.ijnonlinmec.2018.08.018. URL https://www.sciencedirect.com/science/article/pii/S002074621830163X.

[160] Holzapfel Gerhard A., Niestrawska Justyna A., Ogden Ray W., Reinisch Andreas J., and Schriefl Andreas J.. Modelling non-symmetric collagen fibre dispersion in arterial walls. Journal of The Royal Society Interface, 12 (106):20150188, May 2015. doi:10.1098/rsif.2015.0188. URL https://royalsocietypublishing.org/doi/full/10.1098/rsif.2015.0188. Publisher: Royal Society. PMC4424700 25878125

[161] Guan Debao, Zhuan Xin, Holmes William, Luo Xiaoyu, and Gao Hao. Modelling of fibre dispersion and its effects on cardiac mechanics from diastole to systole. Journal of Engineering Mathematics, 128(1):1, April 2021. ISSN 1573-2703. doi:10.1007/s10665-021-10102-w. URL https://doi.org/10.1007/s10665-021-10102-w.

[162] Cortes Daniel H., Lake Spencer P., Kadlowec Jennifer A., Soslowsky Louis J., and Elliott Dawn M.. Characterizing the mechanical contribution of fiber angular distribution in connective tissue: comparison of two modeling approaches. Biomechanics and Modeling in Mechanobiology, 9(5):651–658, October 2010. ISSN 1617-7940. doi:10.1007/s10237-010-0194-x. URL https://doi.org/10.1007/s10237-010-0194-x. 20148345 PMC2917498

[163] Holzapfel Gerhard A. and Ogden Ray W.. On Fiber Dispersion Models: Exclusion of Compressed Fibers and Spurious Model Comparisons. Journal of Elasticity, 129(1):49–68, December 2017. ISSN 1573-2681. doi:10.1007/s10659-016-9605-2. URL https://doi.org/10.1007/s10659-016-9605-2.

[164] Holzapfel Gerhard A. and Ogden Ray W.. Comparison of two model frameworks for fiber dispersion in the elasticity of soft biological tissues. European Journal of Mechanics - A/Solids, 66:193–200, November 2017. ISSN 0997-7538. doi:10.1016/j.euromechsol.2017.07.005. URL https://www.sciencedirect.com/science/article/pii/S0997753817303807.

[165] Li Kewei, Ogden Ray W., and Holzapfel Gerhard A.. Modeling fibrous biological tissues with a general invariant that excludes compressed fibers. Journal of the Mechanics and Physics of Solids, 110:38–53, January 2018. ISSN 0022-5096. doi:10.1016/j.jmps.2017.09.005. URL https://www.sciencedirect.com/science/article/pii/S0022509617304672.

[166] Li Kewei, Ogden Ray W., and Holzapfel Gerhard A.. A discrete fibre dispersion method for excluding fibres under compression in the modelling of fibrous tissues. Journal of The Royal Society Interface, 15(138):20170766, January 2018. doi:10.1098/rsif.2017.0766. URL https://royalsocietypublishing.org/doi/full/10.1098/rsif.2017.0766. Publisher: Royal Society. PMC5805975 29386399

[167] Lin D. H. S. and Yin F. C. P.. A Multiaxial Constitutive Law for Mammalian Left Ventricular Myocardium in Steady-State Barium Contracture or Tetanus. Journal of Biomechanical Engineering, 120(4):504–517, August 1998. ISSN 0148-0731. doi:10.1115/1.2798021. URL https://doi.org/10.1115/1.2798021. 10412422

[168] Krishnamurthy Adarsh, Coppola Benjamin, Tangney Jared, Kerckhoffs Roy C. P., Omens Jeffrey H., and McCulloch Andrew D.. A Microstructurally Based Multi-Scale Constitutive Model of Active Myocardial Mechanics. In Kassab Ghassan S and Sacks Michael S., editors, Structure-Based Mechanics of Tissues and Organs, pages 439–460. Springer US, Boston, MA, 2016. ISBN 978-1-4899-7630-7. doi:10.1007/978-1-4899-7630-7_22. URL https://doi.org/10.1007/978-1-4899-7630-7_22.

[169] Piersanti Roberto, Regazzoni Francesco, Salvador Matteo, Corno Antonio F., Dede’ Luca, Vergara Christian, and Quarteroni Alfio. 3D–0D closed-loop model for the simulation of cardiac biventricular electromechanics. Computer Methods in Applied Mechanics and Engineering, 391:114607, March 2022. ISSN 0045-7825. doi:10.1016/j.cma.2022.114607. URL https://www.sciencedirect.com/science/article/pii/S0045782522000251.

[170] Sack Kevin L., Aliotta Eric, Ennis Daniel B., Choy Jenny S., Kassab Ghassan S., Guccione Julius M., and Franz Thomas. Construction and Validation of Subject-Specific Biventricular Finite-Element Models of Healthy and Failing Swine Hearts From High-Resolution DT-MRI. Frontiers in Physiology, 9, May 2018. ISSN 1664-042X. doi:10.3389/fphys.2018.00539. URL https://www.frontiersin.org/journals/physiology/articles/10.3389/fphys.2018.00539/full. Publisher: Frontiers. PMC5986944 29896107

[171] Gerach Tobias, Schuler Steffen, Fröhlich Jonathan, Lindner Laura, Kovacheva Ekaterina, Moss Robin, Wülfers Eike Moritz, Seemann Gunnar, Wieners Christian, and Loewe Axel. Electro-Mechanical Whole-Heart Digital Twins: A Fully Coupled Multi-Physics Approach. Mathematics, 9(11):1247, January 2021. ISSN 2227-7390. doi:10.3390/math9111247. URL https://www.mdpi.com/2227-7390/9/11/1247. Number: 11 Publisher: Multidisciplinary Digital Publishing Institute.

[172] Fritz Thomas, Wieners Christian, Seemann Gunnar, Steen Henning, and Olaf Dössel. Simulation of the contraction of the ventricles in a human heart model including atria and pericardium. Biomechanics and Modeling in Mechanobiology, 13(3):627–641, June 2014. ISSN 1617-7940. doi:10.1007/s10237-013-0523-y. URL https://doi.org/10.1007/s10237-013-0523-y. 23990017

[173] Ghebryal Justina, Rodero Cristobal, Barrows Rosie K., Strocchi Marina, Roney Caroline H., Augustin Christoph M., Plank Gernot, and Niederer Steven A.. Effect of Varying Pericardial Boundary Conditions on Whole Heart Function: A Computational Study. In Bernard Olivier, Clarysse Patrick, Duchateau Nicolas, Ohayon Jacques, and Viallon Magalie, editors, Functional Imaging and Modeling of the Heart, pages 545–554, Cham, 2023. Springer Nature Switzerland. ISBN 978-3-031-35302-4. doi:10.1007/978-3-031-35302-4_56.

[174] Feng Liuyang, Gao Hao, and Luo Xiaoyu. Whole-heart modelling with valves in a fluid–structure interaction framework. Computer Methods in Applied Mechanics and Engineering, 420:116724, February 2024. ISSN 0045-7825. doi:10.1016/j.cma.2023.116724. URL https://www.sciencedirect.com/science/article/pii/S0045782523008472.

[175] Asner L., Hadjicharalambous M., Chabiniok R., Peressutti D., Sammut E., Wong J., Carr-White G., Razavi R., King A. P., Smith N., Lee J., and Nordsletten D.. Patient-specific modeling for left ventricular mechanics using data-driven boundary energies. Computer Methods in Applied Mechanics and Engineering, 314:269–295, February 2017. ISSN 0045-7825. doi:10.1016/j.cma.2016.08.002. URL https://www.sciencedirect.com/science/article/pii/S0045782516308672.

[176] Rodero Cristobal, Strocchi Marina, Marciniak Maciej, Longobardi Stefano, Whitaker John, O’Neill Mark D., Gillette Karli, Augustin Christoph, Plank Gernot, Vigmond Edward J., Lamata Pablo, and Niederer Steven A.. Linking statistical shape models and simulated function in the healthy adult human heart. PLOS Computational Biology, 17(4):e1008851, April 2021. ISSN 1553-7358. doi:10.1371/journal.pcbi.1008851. URL https://journals.plos.org/ploscompbiol/article?id=10.1371/journal.pcbi.1008851. Publisher: Public Library of Science. PMC8049237 33857152

[177] Niederer Steven, Rhode Kawal, Razavi Reza, and Smith Nic. The Importance of Model Parameters and Boundary Conditions in Whole Organ Models of Cardiac Contraction. In Ayache Nicholas, Delingette Hervé, and Sermesant Maxime, editors, Functional Imaging and Modeling of the Heart, pages 348–356, Berlin, Heidelberg, 2009. Springer. ISBN 978-3-642-01932-6. doi:10.1007/978-3-642-01932-6_38.

[178] Naeije Robert and Badagliacca Roberto. The overloaded right heart and ventricular interdependence. Cardiovascular Research, 113(12):1474–1485, October 2017. ISSN 0008-6363. doi:10.1093/cvr/cvx160. URL https://doi.org/10.1093/cvr/cvx160. 28957537

[179] Mandapaka Sangeeta, Hamilton Craig A., Morgan Timothy M., and Hundley W. Gregory. Simultaneous Measurement of Left and Right Ventricular Volumes and Ejection Fraction During Dobutamine Stress Cardiovascular Magnetic Resonance. Journal of computer assisted tomography, 35(5):614–617, 2011. ISSN 0363-8715. doi:10.1097/RCT.0b013e31822abbcd. URL https://www.ncbi.nlm.nih.gov/pmc/articles/PMC3666038/. 21926858 PMC3666038

[180] Kim Salla M., Randall E. Benjamin, Jezek Filip, Beard Daniel A., and Chesler Naomi C.. Computational modeling of ventricular-ventricular interactions suggest a role in clinical conditions involving heart failure. Frontiers in Physiology, 14, September 2023. ISSN 1664-042X. doi:10.3389/fphys.2023.1231688. URL https://www.frontiersin.org/journals/physiology/articles/10.3389/fphys.2023.1231688/full. Publisher: Frontiers. PMC10512181 37745253

[181] Santamore W. P., Shaffer T., and Papa L.. Theoretical model of ventricular interdependence: pericardial effects. American Journal of Physiology-Heart and Circulatory Physiology, 259(1):H181–H189, July 1990. ISSN 0363-6135. doi:10.1152/ajpheart.1990.259.1.H181. URL https://journals.physiology.org/doi/abs/10.1152/ajpheart.1990.259.1.h181. Publisher: American Physiological Society. 2375404

[182] Carol A. Kroeker Gibbons, Shrive Nigel G., Belenkie Israel, and Tyberg John V.. Pericardium modulates left and right ventricular stroke volumes to compensate for sudden changes in atrial volume. American Journal of Physiology-Heart and Circulatory Physiology, 284(6):H2247–H2254, June 2003. ISSN 0363-6135. doi:10.1152/ajpheart.00613.2002. URL https://journals.physiology.org/doi/full/10.1152/ajpheart.00613.2002. Publisher: American Physiological Society. 12560206

[183] Vaillant Fanny, Abell Emma, Bear Laura R., Caluori Guido, Belterman Charly, Coronel Ruben, Ploux Sylvain, and Dos Santos Pierre. Influence of pericardium on ventricular mechanical interdependence in an isolated biventricular working pig heart model. The Journal of Physiology, 603(2):285–300, 2025. ISSN 1469-7793. doi:10.1113/JP286259. URL https://onlinelibrary.wiley.com/doi/abs/10.1113/JP286259. _eprint: https://onlinelibrary.wiley.com/doi/pdf/10.1113/JP286259. 39724308 PMC11737541

[184] Damiano R. J., La Follette P., Cox J. L., Lowe J. E., and Santamore W. P.. Significant left ventricular contribution to right ventricular systolic function. American Journal of Physiology-Heart and Circulatory Physiology, 261 (5):H1514–H1524, November 1991. ISSN 0363-6135. doi:10.1152/ajpheart.1991.261.5.H1514. URL https://journals.physiology.org/doi/abs/10.1152/ajpheart.1991.261.5.H1514. Publisher: American Physiological Society. 1951739

[185] Petit Matthieu and Vieillard-Baron Antoine. Ventricular interdependence in critically ill patients: from physiology to bedside. Frontiers in Physiology, 14:1232340, August 2023. ISSN 1664-042X. doi:10.3389/fphys.2023.1232340. URL https://www.ncbi.nlm.nih.gov/pmc/articles/PMC10442576/. PMC10442576 37614759

[186] Chung D. C., Niranjan S. C., Clark J. W., Bidani A., Johnston W. E., Zwischenberger J. B., and Traber D. L.. A dynamic model of ventricular interaction and pericardial influence. American Journal of Physiology-Heart and Circulatory Physiology, 272(6):H2942–H2962, June 1997. ISSN 0363-6135. doi:10.1152/ajpheart.1997.272.6.H2942. URL https://journals.physiology.org/doi/abs/10.1152/ajpheart.1997.272.6.h2942. Publisher: American Physiological Society. 9227574

[187] Sack Kevin L., Dabiri Yaghoub, Franz Thomas, Solomon Scott D., Burkhoff Daniel, and Guccione Julius M.. Investigating the Role of Interventricular Interdependence in Development of Right Heart Dysfunction During LVAD Support: A Patient-Specific Methods-Based Approach. Frontiers in Physiology, 9, May 2018. ISSN 1664-042X. doi:10.3389/fphys.2018.00520. URL https://www.frontiersin.orgundefined/journals/physiology/articles/10.3389/fphys.2018.00520/full. Publisher: Frontiers. PMC5962934 29867563

[188] Beyar R. and Sideman S.. Atrioventricular interactions: a theoretical simulation study. American Journal of Physiology-Heart and Circulatory Physiology, 252(3):H653–H665, March 1987. ISSN 0363-6135. doi:10.1152/ajpheart.1987.252.3.H653. URL https://journals.physiology.org/doi/abs/10.1152/ajpheart.1987.252.3.H653. Publisher: American Physiological Society. 3826407

[189] Blume Gustavo G., Mcleod Christopher J., Barnes Marion E., Seward James B., Pellikka Patricia A., Bastiansen Paul M., and Tsang Teresa S.M.. Left atrial function: physiology, assessment, and clinical implications. European Journal of Echocardiography, 12(6):421–430, June 2011. ISSN 1525-2167. doi:10.1093/ejechocard/jeq175. URL https://doi.org/10.1093/ejechocard/jeq175. 21565866

[190] Strocchi Marina, Augustin Christoph M., Gsell Matthias A. F., Karabelas Elias, Neic Aurel, Gillette Karli, Roney Caroline H., Razeghi Orod, Behar Jonathan M., Rinaldi Christopher A., Vigmond Edward J., Bishop Martin J., Plank Gernot, and Niederer Steven A.. The Effect of Ventricular Myofibre Orientation on Atrial Dynamics. In Ennis Daniel B., Perotti Luigi E., and Wang Vicky Y., editors, Functional Imaging and Modeling of the Heart, pages 659–670, Cham, 2021. Springer International Publishing. ISBN 978-3-030-78710-3. doi:10.1007/978-3-030-78710-3_63.

[191] Land S. and Niederer S.A.. Influence of atrial contraction dynamics on cardiac function. International Journal for Numerical Methods in Biomedical Engineering, 34(3), 2018. doi:10.1002/cnm.2931. 28990354

[192] Gerach Tobias, Schuler Steffen, Wachter Andreas, and Loewe Axel. The Impact of Standard Ablation Strategies for Atrial Fibrillation on Cardiovascular Performance in a Four-Chamber Heart Model. Cardiovascular Engineering and Technology, 14(2):296–314, April 2023. ISSN 1869-4098. doi:10.1007/s13239-022-00651-1. URL https://doi.org/10.1007/s13239-022-00651-1. 36652165 PMC10102113

[193] Fatemifar Fatemeh, Feldman Marc D., Clarke Geoffrey D., Finol Ender A., and Han Hai-Chao. Computational Modeling of Human Left Ventricle to Assess the Effects of Trabeculae Carneae on the Diastolic and Systolic Functions. Journal of Biomechanical Engineering, 141(091014), August 2019. ISSN 0148-0731. doi:10.1115/1.4043831. URL https://doi.org/10.1115/1.4043831. 31116359

[194] Serrani Marta, Costantino Maria Laura, and Fumero Roberto. The influence of cardiac trabeculae on ventricular mechanics. In Computing in Cardiology 2013, pages 811–814, September 2013. URL https://ieeexplore.ieee.org/abstract/document/6713501. ISSN: 2325–8853.

[195] Vedula Vijay, Seo Jung-Hee, Lardo Albert C., and Mittal Rajat. Effect of trabeculae and papillary muscles on the hemodynamics of the left ventricle. Theoretical and Computational Fluid Dynamics, 30(1):3–21, April 2016. ISSN 1432-2250. doi:10.1007/s00162-015-0349-6. URL https://doi.org/10.1007/s00162-015-0349-6.

[196] Rene Rodriguez E. and Carmela D. Tan. Structure and Anatomy of the Human Pericardium. Progress in Cardiovascular Diseases, 59(4):327–340, January 2017. ISSN 0033-0620. doi:10.1016/j.pcad.2016.12.010. URL https://www.sciencedirect.com/science/article/pii/S0033062016301463. 28062264

[197] Brown Aaron L., Salvador Matteo, Shi Lei, Pfaller Martin R., Hu Zinan, Harold Kaitlin E., Hsiai Tzung, Vedula Vijay, and Marsden Alison L.. A modular framework for implicit 3D–0D coupling in cardiac mechanics. Computer Methods in Applied Mechanics and Engineering, 421:116764, March 2024. ISSN 0045-7825. doi:10.1016/j.cma.2024.116764. URL https://www.sciencedirect.com/science/article/pii/S0045782524000203. PMC10956732 38523716

[198] Brenneisen Jochen, Daub Anna, Gerach Tobias, Kovacheva Ekaterina, Huetter Larissa, Frohnapfel Bettina, Dössel Olaf, and Loewe Axel. Sequential Coupling Shows Minor Effects of Fluid Dynamics on Myocardial Deformation in a Realistic Whole-Heart Model. Frontiers in Cardiovascular Medicine, 8, December 2021. ISSN 2297-055X. doi:10.3389/fcvm.2021.768548. URL https://www.frontiersin.org/journals/cardiovascular-medicine/articles/10.3389/fcvm.2021.768548/full. Publisher: Frontiers. PMC8733159 35004885

[199] Davey Marshall, Puelz Charles, Rossi Simone, Smith Margaret Anne, Wells David R, Sturgeon Gregory M, Segars W Paul, Vavalle John P, Peskin Charles S, and Griffith Boyce E. Simulating cardiac fluid dynamics in the human heart. PNAS Nexus, 3(10):pgae392, October 2024. ISSN 2752-6542. doi:10.1093/pnasnexus/pgae392. URL https://doi.org/10.1093/pnasnexus/pgae392. PMC11492567 39434870

[200] Regazzoni F., Dedè L., and Quarteroni A.. Machine learning of multiscale active force generation models for the efficient simulation of cardiac electromechanics. Computer Methods in Applied Mechanics and Engineering, 370:113268, October 2020. ISSN 0045-7825. doi:10.1016/j.cma.2020.113268. URL https://www.sciencedirect.com/science/article/pii/S0045782520304539.

[201] Regazzoni F., Salvador M., Africa P. C., Fedele M., Dedè L., and Quarteroni A.. A cardiac electromechanical model coupled with a lumped-parameter model for closed-loop blood circulation. Journal of Computational Physics, 457:111083, May 2022. ISSN 0021-9991. doi:10.1016/j.jcp.2022.111083. URL https://www.sciencedirect.com/science/article/pii/S0021999122001450.

[202] Talman Andrew H., Psaltis Peter J., Cameron James D., Meredith Ian T., Seneviratne Sujith K., and Wong Dennis T. L.. Epicardial adipose tissue: far more than a fat depot. Cardiovascular Diagnosis and Therapy, 4(6):416–429, December 2014. ISSN 2223-3652. doi:10.3978/j.issn.2223-3652.2014.11.05. URL https://www.ncbi.nlm.nih.gov/pmc/articles/PMC4278038/. 25610800 PMC4278038

[203] Mendiola Emilio A., Sacks Michael S., and Avazmohammadi Reza. Mechanical Interaction of the Pericardium and Cardiac Function in the Normal and Hypertensive Rat Heart. Frontiers in Physiology, 13, May 2022. ISSN 1664-042X. doi:10.3389/fphys.2022.878861. URL https://www.frontiersin.org/journals/physiology/articles/10.3389/fphys.2022.878861/full. Publisher: Frontiers. PMC9108501 35586708

[204] Jilberto Javiera and Nordsletten David. Identification of the Unloaded Heart Configuration Including External Interactions. In Chabiniok Radomír, Zou Qing, Hussain Tarique, Nguyen Hoang H., Zaha Vlad G., and Gusseva Maria, editors, Functional Imaging and Modeling of the Heart, pages 331–342, Cham, 2025. Springer Nature Switzerland. ISBN 978-3-031-94559-5. doi:10.1007/978-3-031-94559-5_30. PMC13098015 42023130

[205] Jonathan Krauß Tobias Gerach, and Loewe Axel. Comparison of Pericardium Modeling Approaches for Mechanical Whole Heart Simulations. In 2023 Computing in Cardiology (CinC), volume 50, pages 1–4, October 2023. doi:10.22489/CinC.2023.150. URL https://ieeexplore.ieee.org/abstract/document/10363870. ISSN: 2325–887X.

[206] Asner Liya, Hadjicharalambous Myrianthi, Chabiniok Radomir, Peresutti Devis, Sammut Eva, Wong James, Carr-White Gerald, Chowienczyk Philip, Lee Jack, King Andrew, Smith Nicolas, Razavi Reza, and Nordsletten David. Estimation of passive and active properties in the human heart using 3D tagged MRI. Biomechanics and Modeling in Mechanobiology, 15(5):1121–1139, October 2016. ISSN 1617-7940. doi:10.1007/s10237-015-0748-z. URL https://doi.org/10.1007/s10237-015-0748-z. 26611908 PMC5021775

[207] Trayanova Natalia A.. Whole-Heart Modeling. Circulation Research, 108(1):113–128, January 2011. doi:10.1161/CIRCRESAHA.110.223610. URL https://www.ahajournals.org/doi/full/10.1161/CIRCRESAHA.110.223610. Publisher: American Heart Association. 21212393 PMC3031963

[208] Potse Mark, Dube Bruno, Richer Jacques, Vinet Alain, and Gulrajani Ramesh M.. A Comparison of Monodomain and Bidomain Reaction-Diffusion Models for Action Potential Propagation in the Human Heart. IEEE Transactions on Biomedical Engineering, 53(12):2425–2435, December 2006. ISSN 1558-2531. doi:10.1109/TBME.2006.880875. URL https://ieeexplore.ieee.org/document/4015619/?arnumber=4015619. Conference Name: IEEE Transactions on Biomedical Engineering. 17153199

[209] Stella Simone, Regazzoni Francesco, Vergara Christian, Dedé Luca, and Quarteroni Alfio. A fast cardiac electromechanics model coupling the Eikonal and the nonlinear mechanics equations. Mathematical Models and Methods in Applied Sciences, 32(08):1531–1556, July 2022. ISSN 0218-2025. doi:10.1142/S021820252250035X. URL https://www.worldscientific.com/doi/10.1142/S021820252250035X. Publisher: World Scientific Publishing Co.

[210] Gurev Viatcheslav, Pathmanathan Pras, Fattebert Jean-Luc, Wen Hui-Fang, Magerlein John, Gray Richard A., Richards David F., and Rice J. Jeremy. A high-resolution computational model of the deforming human heart. Biomechanics and Modeling in Mechanobiology, 14(4):829–849, August 2015. ISSN 1617-7940. doi:10.1007/s10237-014-0639-8. URL https://link.springer.com/article/10.1007/s10237-014-0639-8. Company: Springer Distributor: Springer Institution: Springer Label: Springer Number: 4 Publisher: Springer Berlin Heidelberg. 25567753

[211] Fan Lei, Choy Jenny S., Raissi Farshad, Kassab Ghassan S., and Lee Lik Chuan. Optimization of cardiac resynchronization therapy based on a cardiac electromechanics-perfusion computational model. Computers in Biology and Medicine, 141:105050, February 2022. ISSN 0010-4825. doi:10.1016/j.compbiomed.2021.105050. URL https://www.sciencedirect.com/science/article/pii/S0010482521008441. PMC8810745 34823858

[212] Radišić Irena, Regazzoni Francesco, Bucelli Michele, Pagani Stefano, Dede’ Luca, and Quarteroni Alfio. Influence of cellular mechano-calcium feedback in numerical models of cardiac electromechanics. Computer Methods in Applied Mechanics and Engineering, 445:118172, October 2025. ISSN 0045-7825. doi:10.1016/j.cma.2025.118172. URL https://www.sciencedirect.com/science/article/pii/S004578252500444X.

[213] Jung Alexander, Gsell Matthias A. F., Augustin Christoph M., and Plank Gernot. An Integrated Workflow for Building Digital Twins of Cardiac Electromechanics—A Multi-Fidelity Approach for Personalising Active Mechanics. Mathematics, 10(5):823, January 2022. ISSN 2227-7390. doi:10.3390/math10050823. URL https://www.mdpi.com/2227-7390/10/5/823. Number: 5 Publisher: Multidisciplinary Digital Publishing Institute. 35295404 PMC7612499

[214] Regazzoni Francesco, Dedè Luca, and Quarteroni Alfio. Biophysically detailed mathematical models of multiscale cardiac active mechanics. PLOS Computational Biology, 16(10):e1008294, October 2020. ISSN 1553-7358. doi:10.1371/journal.pcbi.1008294. URL https://journals.plos.org/ploscompbiol/article?id=10.1371/journal.pcbi.1008294. Publisher: Public Library of Science. PMC7571720 33027247

[215] Gerach Tobias and Loewe Axel. Differential effects of mechano-electric feedback mechanisms on whole-heart activation, repolarization, and tension. The Journal of Physiology, 602(18):4605–4624, 2024. ISSN 1469-7793. doi:10.1113/JP285022. URL https://onlinelibrary.wiley.com/doi/abs/10.1113/JP285022. _eprint: https://physoc.onlinelibrary.wiley.com/doi/pdf/10.1113/JP285022. 38185911

[216] Alexander Quinn T. and Kohl Peter. Cardiac Mechano-Electric Coupling: Acute Effects of Mechanical Stimulation on Heart Rate and Rhythm. Physiological Reviews, 101(1):37–92, January 2021. ISSN 0031-9333. doi:10.1152/physrev.00036.2019. URL https://journals.physiology.org/doi/full/10.1152/physrev.00036.2019. Publisher: American Physiological Society. 32380895

[217] Salvador Matteo, Regazzoni Francesco, Pagani Stefano, Dede’ Luca, Trayanova Natalia, and Quarteroni Alfio. The role of mechano-electric feedbacks and hemodynamic coupling in scar-related ventricular tachycardia. Computers in Biology and Medicine, 142:105203, March 2022. ISSN 0010-4825. doi:10.1016/j.compbiomed.2021.105203. URL https://www.sciencedirect.com/science/article/pii/S0010482521009975. 35033878

[218] Colli Franzone P., Pavarino L. F., and Scacchi S.. Effects of mechanical feedback on the stability of cardiac scroll waves: A bidomain electro-mechanical simulation study. Chaos: An Interdisciplinary Journal of Nonlinear Science, 27(9):093905, September 2017. ISSN 1054-1500, 1089–7682. doi:10.1063/1.4999465. URL https://pubs.aip.org/cha/article/27/9/093905/342097/Effects-of-mechanical-feedback-on-the-stability-of. 28964121

[219] Costabal Francisco Sahli, Concha Felipe A., Hurtado Daniel E., and Kuhl Ellen. The importance of mechanoelectrical feedback and inertia in cardiac electromechanics. Computer Methods in Applied Mechanics and Engineering, 320:352–368, June 2017. ISSN 0045-7825. doi:10.1016/j.cma.2017.03.015. URL https://www.sciencedirect.com/science/article/pii/S0045782516317728. 29056782 PMC5646712

[220] Salvador Matteo, Dede’ Luca, and Quarteroni Alfio. An intergrid transfer operator using radial basis functions with application to cardiac electromechanics. Computational Mechanics, 66(2):491–511, August 2020. ISSN 1432-0924. doi:10.1007/s00466-020-01861-x. URL https://doi.org/10.1007/s00466-020-01861-x.

[221] Regazzoni F. and Quarteroni A.. An oscillation-free fully staggered algorithm for velocity-dependent active models of cardiac mechanics. Computer Methods in Applied Mechanics and Engineering, 373:113506, January 2021. ISSN 0045-7825. doi:10.1016/j.cma.2020.113506. URL https://www.sciencedirect.com/science/article/pii/S0045782520306915.

[222] Clayton R. H., Bernus O., Cherry E. M., Dierckx H., Fenton F. H., Mirabella L., Panfilov A. V., Sachse F. B., Seemann G., and Zhang H.. Models of cardiac tissue electrophysiology: Progress, challenges and open questions. Progress in Biophysics and Molecular Biology, 104(1):22–48, January 2011. ISSN 0079-6107. doi:10.1016/j.pbiomolbio.2010.05.008. URL https://www.sciencedirect.com/science/article/pii/S0079610710000362. 20553746

[223] Quarteroni Alfio, Lassila Toni, Rossi Simone, and Ricardo Ruiz-Baier. Integrated Heart—Coupling multiscale and multiphysics models for the simulation of the cardiac function. Computer Methods in Applied Mechanics and Engineering, 314:345–407, February 2017. ISSN 0045-7825. doi:10.1016/j.cma.2016.05.031. URL https://www.sciencedirect.com/science/article/pii/S0045782516304662.

[224] Verzicco R.. Electro-fluid-mechanics of the heart. Journal of Fluid Mechanics, 941:P1, June 2022. ISSN 0022-1120, 1469–7645. doi:10.1017/jfm.2022.272. URL https://www.cambridge.org/core/journals/journal-of-fluid-mechanics/article/electrofluidmechanics-of-the-heart/6BDA989AA6F798872F10624C6D786B09.

[225] Viola Francesco, Del Corso Giulio, and Verzicco Roberto. High-fidelity model of the human heart: An immersed boundary implementation. Physical Review Fluids, 8(10):100502, October 2023. doi:10.1103/PhysRevFluids.8.100502. URL https://link.aps.org/doi/10.1103/PhysRevFluids.8.100502. Publisher: American Physical Society.

[226] Bucelli Michele, Zingaro Alberto, Africa Pasquale Claudio, Fumagalli Ivan, Dede’ Luca, and Quarteroni Alfio. A mathematical model that integrates cardiac electrophysiology, mechanics, and fluid dynamics: Application to the human left heart. International Journal for Numerical Methods in Biomedical Engineering, 39(3):e3678, 2023. ISSN 2040-7947. doi:10.1002/cnm.3678. URL https://onlinelibrary.wiley.com/doi/abs/10.1002/cnm.3678. _eprint: https://onlinelibrary.wiley.com/doi/pdf/10.1002/cnm.3678. 36579792

[227] Zingaro Alberto, Bucelli Michele, Piersanti Roberto, Regazzoni Francesco, Dede’ Luca, and Quarteroni Alfio. An electromechanics-driven fluid dynamics model for the simulation of the whole human heart. Journal of Computational Physics, 504:112885, May 2024. ISSN 0021-9991. doi:10.1016/j.jcp.2024.112885. URL https://www.sciencedirect.com/science/article/pii/S0021999124001347.

[228] Quarteroni A., Veneziani A., and Vergara C.. Geometric multiscale modeling of the cardiovascular system, between theory and practice. Computer Methods in Applied Mechanics and Engineering, 302:193–252, April 2016. ISSN 0045-7825. doi:10.1016/j.cma.2016.01.007. URL https://www.sciencedirect.com/science/article/pii/S0045782516000098.

[229] Caforio Federica, Augustin Christoph M., Alastruey Jordi, Gsell Matthias A. F., and Plank Gernot. A coupling strategy for a first 3D-1D model of the cardiovascular system to study the effects of pulse wave propagation on cardiac function. Computational Mechanics, 70(4):703–722, October 2022. ISSN 1432-0924. doi:10.1007/s00466-022-02206-6. URL https://doi.org/10.1007/s00466-022-02206-6. 36124206 PMC9477941

[230] Peiró Joaquim and Veneziani Alessandro. Reduced models of the cardiovascular system. In Cardiovascular Mathematics, pages 347–394. Springer, Milano, 2009. ISBN 978-88-470-1152-6. doi:10.1007/978-88-470-1152-6_10. URL https://link.springer.com/chapter/10.1007/978-88-470-1152-6_10.

[231] Moghadam Mahdi Esmaily, Vignon-Clementel Irene E., Figliola Richard, and Marsden Alison L.. A modular numerical method for implicit 0D/3D coupling in cardiovascular finite element simulations. Journal of Computational Physics, 244:63–79, July 2013. ISSN 0021-9991. doi:10.1016/j.jcp.2012.07.035. URL https://www.sciencedirect.com/science/article/pii/S0021999112004202.

[232] Jafari Arian, Pszczolkowski Edward, and Krishnamurthy Adarsh. A Framework for Biomechanics Simulations Using Four-Chamber Cardiac Models. Journal of biomechanics, 91:92–101, June 2019. ISSN 0021-9290. doi:10.1016/j.jbiomech.2019.05.019. URL https://www.ncbi.nlm.nih.gov/pmc/articles/PMC6579665/. 31155211 PMC6579665

[233] Shavik Sheikh Mohammad, Jiang Zhenxiang, Baek Seungik, and Lee Lik Chuan. High Spatial Resolution Multi-Organ Finite Element Modeling of Ventricular-Arterial Coupling. Frontiers in Physiology, 9:119, 2018. ISSN 1664-042X. doi:10.3389/fphys.2018.00119. 29551977 PMC5841309

[234] Westerhof Nico, Lankhaar Jan-willem, and Westerhof Berend E.. The arterial Windkessel. Medical and Biological Engineering and Computing, 47(2):131–41, February 2009. ISSN 01400118. doi:10.1007/s11517-008-0359-2. URL https://www.proquest.com/docview/211490078/abstract/CFC7CEDB521A43E5PQ/1. Num Pages: 131–41 Place: Heidelberg, Netherlands Publisher: Springer Nature B.V. 18543011

[235] Kung Ethan, Baretta Alessia, Baker Catriona, Arbia Gregory, Biglino Giovanni, Corsini Chiara, Schievano Silvia, Vignon-Clementel Irene E., Dubini Gabriele, Pennati Giancarlo, Taylor Andrew, Dorfman Adam, Hlavacek Anthony M., Marsden Alison L., Hsia Tain-Yen, Migliavacca Francesco, and Modeling Of Congenital Hearts Alliance (MOCHA)+ Investigators. Predictive modeling of the virtual Hemi-Fontan operation for second stage single ventricle palliation: two patient-specific cases. Journal of Biomechanics, 46(2):423–429, January 2013. ISSN 1873-2380. doi:10.1016/j.jbiomech.2012.10.023. 23174419

[236] Vincent Jean-Louis. Understanding cardiac output. Critical Care, 12(4):174, 2008. ISSN 1364-8535. doi:10.1186/cc6975. URL https://www.ncbi.nlm.nih.gov/pmc/articles/PMC2575587/. 18771592 PMC2575587

[237] Kung Ethan, Pennati Giancarlo, Migliavacca Francesco, Hsia Tain-Yen, Figliola Richard, Marsden Alison, Giardini Alessandro, and MOCHA Investigators. A Simulation Protocol for Exercise Physiology in Fontan Patients Using a Closed Loop Lumped-Parameter Model. Journal of Biomechanical Engineering, 136(081007), June 2014. ISSN 0148-0731. doi:10.1115/1.4027271. URL https://doi.org/10.1115/1.4027271. PMC5101033 24658635

[238] Kaufmann Joshua and Kung Ethan. Factors Affecting Cardiovascular Physiology in Cardiothoracic Surgery: Implications for Lumped-Parameter Modeling. Frontiers in Surgery, 6, November 2019. ISSN 2296-875X. doi:10.3389/fsurg.2019.00062. URL https://www.frontiersin.org/journals/surgery/articles/10.3389/fsurg.2019.00062/full. Publisher: Frontiers. PMC6848453 31750311

[239] Van De Bruaene Alexander, Claessen Guido, La Gerche Andre, Kung Ethan, Marsden Alison, De Meester Pieter, Devroe Sarah, Bogaert Jan, Claus Piet, Heidbuchel Hein, Budts Werner, and Gewillig Marc. Effect of respiration on cardiac filling at rest and during exercise in Fontan patients: A clinical and computational modeling study. IJC Heart & Vasculature, 9:100–108, December 2015. ISSN 2352-9067. doi:10.1016/j.ijcha.2015.08.002. URL https://www.sciencedirect.com/science/article/pii/S2352906715300257. 28785717 PMC5497350

[240] Kariya Taro, Washio Takumi, Okada Jun-ichi, Nakagawa Machiko, Watanabe Masahiro, Kadooka Yoshimasa, Sano Shunji, Nagai Ryozo, Sugiura Seiryo, and Hisada Toshiaki. Personalized Perioperative Multi-scale, Multi-physics Heart Simulation of Double Outlet Right Ventricle. Annals of Biomedical Engineering, 48(6): 1740–1750, June 2020. ISSN 1573-9686. doi:10.1007/s10439-020-02488-y. URL https://doi.org/10.1007/s10439-020-02488-y. 32152800

[241] Regazzoni Francesco. Stabilization of loosely coupled schemes for 0D–3D fluid–structure interaction problems with application to cardiovascular modelling. Numerische Mathematik, 157(1):249–306, February 2025. ISSN 0945-3245. doi:10.1007/s00211-025-01452-z. URL https://doi.org/10.1007/s00211-025-01452-z.

[242] Hirschvogel Marc, Bonini Mia, Balmus Maximilian, and Nordsletten David. Effective block preconditioners for fluid dynamics coupled to reduced models of a non-local nature. Computer Methods in Applied Mechanics and Engineering, 435:117541, February 2025. ISSN 0045-7825. doi:10.1016/j.cma.2024.117541. URL https://www.sciencedirect.com/science/article/pii/S0045782524007953.

[243] Esmaily-Moghadam Mahdi, Bazilevs Yuri, and Marsden Alison L.. A new preconditioning technique for implicitly coupled multidomain simulations with applications to hemodynamics. Computational Mechanics, 52(5):1141–1152, November 2013. ISSN 1432-0924. doi:10.1007/s00466-013-0868-1. URL https://link.springer.com/article/10.1007/s00466-013-0868-1. Company: Springer Distributor: Springer Institution: Springer Label: Springer Number: 5 Publisher: Springer Berlin Heidelberg.

[244] Wittwer Nicolás Alejandro Barnafi, Di Gregorio Simone, Dede’ Luca, Zunino Paolo, Vergara Christian, and Quarteroni Alfio. A Multiscale Poromechanics Model Integrating Myocardial Perfusion and the Epicardial Coronary Vessels. SIAM Journal on Applied Mathematics, 82(4):1167–1193, August 2022. ISSN 0036-1399. doi:10.1137/21M1424482. URL https://epubs.siam.org/doi/abs/10.1137/21M1424482. Publisher: Society for Industrial and Applied Mathematics.

[245] Fan Lei, Namani Ravi, Choy Jenny S., Kassab Ghassan S., and Lee Lik Chuan. Transmural Distribution of Coronary Perfusion and Myocardial Work Density Due to Alterations in Ventricular Loading, Geometry and Contractility. Frontiers in Physiology, 12, November 2021. ISSN 1664-042X. doi:10.3389/fphys.2021.744855. URL https://www.frontiersin.org/journals/physiology/articles/10.3389/fphys.2021.744855/full. Publisher: Frontiers. PMC8652301 34899378

[246] Munneke Anneloes G., Lumens Joost, Arts Theo, Prinzen Frits W., and Delhaas Tammo. Myocardial perfusion and flow reserve in the asynchronous heart: mechanistic insight from a computational model. Journal of Applied Physiology, 135(3):489–499, September 2023. ISSN 8750-7587. doi:10.1152/japplphysiol.00181.2023. URL https://journals.physiology.org/doi/full/10.1152/japplphysiol.00181.2023. Publisher: American Physiological Society. 37439238 PMC10538979

[247] Zingaro Alberto, Vergara Christian, Dede’ Luca, Regazzoni Francesco, and Quarteroni Alfio. A comprehensive mathematical model for cardiac perfusion. Scientific Reports, 13(1):14220, August 2023. ISSN 2045-2322. doi:10.1038/s41598-023-41312-0. URL https://www.nature.com/articles/s41598-023-41312-0. Publisher: Nature Publishing Group. 37648701 PMC10469210

[248] Lopez Rachel, Marzban Bahador, Gao Xin, Lauinger Ellen, Van den Bergh Françoise, Whitesall Steven E, Converso-Baran Kimber, Burant Charles F, Michele Daniel E, and Beard Daniel A. Impaired Myocardial Energetics Causes Mechanical Dysfunction in Decompensated Failing Hearts. Function, 1(2):zqaa018, September 2020. ISSN 2633-8823. doi:10.1093/function/zqaa018. URL https://doi.org/10.1093/function/zqaa018. PMC7552914 33074265

[249] Guerrero-Hurtado Manuel, Garcia-Villalba Manuel, Gonzalo Alejandro, Martinez-Legazpi Pablo, Kahn Andrew M., McVeigh Elliot, Bermejo Javier, del Alamo Juan C., and Flores Oscar. Efficient multi-fidelity computation of blood coagulation under flow. PLOS Computational Biology, 19(10):e1011583, October 2023. ISSN 1553-7358. doi:10.1371/journal.pcbi.1011583. URL https://journals.plos.org/ploscompbiol/article?id=10.1371/journal.pcbi.1011583. Publisher: Public Library of Science. PMC10659216 37889899

[250] Sharifi Hossein, Lee Lik Chuan, Campbell Kenneth S., and Wenk Jonathan F.. A multiscale finite element model of left ventricular mechanics incorporating baroreflex regulation. Computers in Biology and Medicine, 168:107690, January 2024. ISSN 0010-4825. doi:10.1016/j.compbiomed.2023.107690. URL https://www.sciencedirect.com/science/article/pii/S0010482523011551. PMC11017291 37984204

[251] Barnafi Nicolás A., Pavarino Luca F., and Scacchi Simone. A comparative study of scalable multilevel preconditioners for cardiac mechanics. Journal of Computational Physics, 492:112421, November 2023. ISSN 0021-9991. doi:10.1016/j.jcp.2023.112421. URL https://www.sciencedirect.com/science/article/pii/S0021999123005168.

[252] Krishnamoorthi Shankarjee, Perotti Luigi E., Borgstrom Nils P., Ajijola Olujimi A., Frid Anna, Ponnaluri Aditya V., Weiss James N., Qu Zhilin, Klug William S., Ennis Daniel B., and Garfinkel Alan. Simulation Methods and Validation Criteria for Modeling Cardiac Ventricular Electrophysiology. PLOS ONE, 9(12): e114494, December 2014. ISSN 1932-6203. doi:10.1371/journal.pone.0114494. URL https://journals.plos.org/plosone/article?id=10.1371/journal.pone.0114494. Publisher: Public Library of Science. PMC4262432 25493967

[253] Rossi Simone, Lassila Toni, Ruiz-Baier Ricardo, Sequeira Adélia, and Quarteroni Alfio. Thermodynamically consistent orthotropic activation model capturing ventricular systolic wall thickening in cardiac electromechanics. European Journal of Mechanics - A/Solids, 48:129–142, November 2014. ISSN 0997-7538. doi:10.1016/j.euromechsol.2013.10.009. URL https://www.sciencedirect.com/science/article/pii/S0997753813001228.

[254] Augustin Christoph M., Neic Aurel, Liebmann Manfred, Prassl Anton J., Niederer Steven A., Haase Gundolf, and Plank Gernot. Anatomically accurate high resolution modeling of human whole heart electromechanics: A strongly scalable algebraic multigrid solver method for nonlinear deformation. Journal of Computational Physics, 305:622–646, January 2016. ISSN 0021-9991. doi:10.1016/j.jcp.2015.10.045. URL https://www.sciencedirect.com/science/article/pii/S0021999115007226. 26819483 PMC4724941

[255] Elguedj T., Bazilevs Y., Calo V. M., and Hughes T. J. R.. $\overset{¯}{B}$ and $\overset{¯}{F}$ projection methods for nearly incompressible linear and non-linear elasticity and plasticity using higher-order NURBS elements. Computer Methods in Applied Mechanics and Engineering, 197(33):2732–2762, June 2008. ISSN 0045-7825. doi:10.1016/j.cma.2008.01.012. URL https://www.sciencedirect.com/science/article/pii/S0045782508000248.

[256] Heisserer Ulrich, Hartmann Stefan, Düster Alexander, and Yosibash Zohar. On volumetric locking-free behaviour of p-version finite elements under finite deformations. Communications in Numerical Methods in Engineering, 24(11):1019–1032, 2008. ISSN 1099-0887. doi:10.1002/cnm.1008. URL https://onlinelibrary.wiley.com/doi/abs/10.1002/cnm.1008. _eprint: https://onlinelibrary.wiley.com/doi/pdf/10.1002/cnm.1008.

[257] Yosibash Zohar. *p*-FEMs in biomechanics: Bones and arteries. Computer Methods in Applied Mechanics and Engineering, 249–252:169–184, December 2012. ISSN 0045-7825. doi:10.1016/j.cma.2012.09.006. URL https://www.sciencedirect.com/science/article/pii/S0045782512002812.

[258] Costa K. D., Hunter P. J., Wayne J. S., Waldman L. K., Guccione J. M., and McCulloch A. D.. A Three-Dimensional Finite Element Method for Large Elastic Deformations of Ventricular Myocardium: II—Prolate Spheroidal Coordinates. Journal of Biomechanical Engineering, 118(4):464–472, November 1996. ISSN 0148-0731. doi:10.1115/1.2796032. URL https://doi.org/10.1115/1.2796032. 8950649

[259] Kerckhoffs Roy C. P., Neal Maxwell L., Gu Quan, Bassingthwaighte James B., Omens Jeff H., and McCulloch Andrew D.. Coupling of a 3D Finite Element Model of Cardiac Ventricular Mechanics to Lumped Systems Models of the Systemic and Pulmonic Circulation. Annals of Biomedical Engineering, 35(1):1–18, January 2007. ISSN 1573-9686. doi:10.1007/s10439-006-9212-7. URL https://doi.org/10.1007/s10439-006-9212-7. 17111210 PMC2872168

[260] Continuity – A Problem Solving Environment for Multi-Scale Biology,. URL https://continuity.ucsd.edu/.

[261] Brezzi Franco and Fortin Michel, editors. Mixed and Hybrid Finite Element Methods, volume 15 of Springer Series in Computational Mathematics. Springer, New York, NY, 1991. ISBN 978-1-4612-7824-5 978-1-4612-3172-1. doi:10.1007/978-1-4612-3172-1. URL http://link.springer.com/10.1007/978-1-4612-3172-1.

[262] Land Sander, Gurev Viatcheslav, Arens Sander, Augustin Christoph M., Baron Lukas, Blake Robert, Bradley Chris, Castro Sebastian, Crozier Andrew, Favino Marco, Fastl Thomas E., Fritz Thomas, Gao Hao, Gizzi Alessio, Griffith Boyce E., Hurtado Daniel E., Krause Rolf, Luo Xiaoyu, Nash Martyn P., Pezzuto Simone, Plank Gernot, Rossi Simone, Ruprecht Daniel, Seemann Gunnar, Smith Nicolas P., Sundnes Joakim, Rice J. Jeremy, Trayanova Natalia, Wang Dafang, Wang Zhinuo Jenny, and Niederer Steven A.. Verification of cardiac mechanics software: benchmark problems and solutions for testing active and passive material behaviour. Proceedings. Mathematical, Physical, and Engineering Sciences, 471(2184):20150641, December 2015. ISSN 1364-5021. doi:10.1098/rspa.2015.0641. PMC4707707 26807042

[263] Nobile F., Quarteroni A., and Ruiz-Baier R.. An active strain electromechanical model for cardiac tissue. International Journal for Numerical Methods in Biomedical Engineering, 28(1):52–71, January 2012. ISSN 2040-7947. doi:10.1002/cnm.1468. 25830205

[264] Lipton S., Evans J. A., Bazilevs Y., Elguedj T., and Hughes T. J. R.. Robustness of isogeometric structural discretizations under severe mesh distortion. Computer Methods in Applied Mechanics and Engineering, 199(5):357–373, January 2010. ISSN 0045-7825. doi:10.1016/j.cma.2009.01.022. URL https://www.sciencedirect.com/science/article/pii/S0045782509000346.

[265] Gültekin Osman, Dal Hüsnü, and Holzapfel Gerhard A.. On the quasi-incompressible finite element analysis of anisotropic hyperelastic materials. Computational Mechanics, 63(3):443–453, March 2019. ISSN 1432-0924. doi:10.1007/s00466-018-1602-9. URL https://link.springer.com/article/10.1007/s00466-018-1602-9. Company: Springer Distributor: Springer Institution: Springer Label: Springer Number: 3 Publisher: Springer Berlin Heidelberg.

[266] Chamberland É., Fortin A., and Fortin M.. Comparison of the performance of some finite element discretizations for large deformation elasticity problems. Computers & Structures, 88(11):664–673, June 2010. ISSN 0045-7949. doi:10.1016/j.compstruc.2010.02.007. URL https://www.sciencedirect.com/science/article/pii/S0045794910000404.

[267] Liu Ju, Marsden Alison L., and Tao Zhen. An energy-stable mixed formulation for isogeometric analysis of incompressible hyperelastodynamics. International Journal for Numerical Methods in Engineering, 120(8): 937–963, 2019. ISSN 1097-0207. doi:10.1002/nme.6165. URL https://onlinelibrary.wiley.com/doi/abs/10.1002/nme.6165. _eprint: https://onlinelibrary.wiley.com/doi/pdf/10.1002/nme.6165. 32981972 PMC7517668

[268] Pezzuto S., Ambrosi D., and Quarteroni A.. An orthotropic active–strain model for the myocardium mechanics and its numerical approximation. European Journal of Mechanics - A/Solids, 48:83–96, November 2014. ISSN 0997-7538. doi:10.1016/j.euromechsol.2014.03.006. URL https://www.sciencedirect.com/science/article/pii/S0997753814000527.

[269] Hughes Thomas J. R., Franca Leopoldo P., and Balestra Marc. A new finite element formulation for computational fluid dynamics: V. Circumventing the babuška-brezzi condition: a stable Petrov-Galerkin formulation of the stokes problem accommodating equal-order interpolations. Computer Methods in Applied Mechanics and Engineering, 59(1):85–99, November 1986. ISSN 0045-7825. doi:10.1016/0045-7825(86)90025-3. URL https://www.sciencedirect.com/science/article/pii/0045782586900253.

[270] Franca Leopoldo P. and Frey Sérgio L.. Stabilized finite element methods: II. The incompressible Navier-Stokes equations. Computer Methods in Applied Mechanics and Engineering, 99(2):209–233, September 1992. ISSN 0045-7825. doi:10.1016/0045-7825(92)90041-H. URL https://www.sciencedirect.com/science/article/pii/004578259290041H.

[271] Hughes Thomas J.R.. Multiscale phenomena: Green’s functions, the Dirichlet-to-Neumann formulation, subgrid scale models, bubbles and the origins of stabilized methods. Computer Methods in Applied Mechanics and Engineering, 127(1–4):387–401, November 1995. ISSN 00457825. doi:10.1016/0045-7825(95)00844-9. URL https://linkinghub.elsevier.com/retrieve/pii/0045782595008449.

[272] Bazilevs Y., Takizawa K., and Tezduyar T.E.. Computational Fluid–Structure Interaction: Methods and Applications. John Wiley & Sons, Ltd, 1 edition, 2013. doi:10.1002/9781118483565. URL https://onlinelibrary.wiley.com/doi/10.1002/9781118483565. _eprint: https://onlinelibrary.wiley.com/doi/pdf/10.1002/9781118483565.

[273] John Volker and Kindl Adela. Numerical studies of finite element variational multiscale methods for turbulent flow simulations. Computer Methods in Applied Mechanics and Engineering, 199(13):841–852, February 2010. ISSN 0045-7825. doi:10.1016/j.cma.2009.01.010. URL https://www.sciencedirect.com/science/article/pii/S0045782509000395.

[274] Updegrove Adam, Wilson Nathan M., Merkow Jameson, Lan Hongzhi, Marsden Alison L., and Shadden Shawn C.. SimVascular: An Open Source Pipeline for Cardiovascular Simulation. Annals of Biomedical Engineering, 45(3):525–541, March 2017. ISSN 1573-9686. doi:10.1007/s10439-016-1762-8. URL https://link.springer.com/article/10.1007/s10439-016-1762-8. Company: Springer Distributor: Springer Institution: Springer Label: Springer Number: 3 Publisher: Springer US. 27933407 PMC6546171

[275] Alberto Figueroa C., Taylor Charles A., and Marsden Alison L.. Blood Flow. In Encyclopedia of Computational Mechanics Second Edition, pages 1–31. John Wiley & Sons, Ltd, 2017. ISBN 978-1-119-17681-7. doi:10.1002/9781119176817.ecm2068. URL https://onlinelibrary.wiley.com/doi/abs/10.1002/9781119176817.ecm2068. _eprint: https://onlinelibrary.wiley.com/doi/pdf/10.1002/9781119176817.ecm2068.

[276] Tagliabue A., Dedè L., and Quarteroni A.. Fluid dynamics of an idealized left ventricle: the extended Nitsche’s method for the treatment of heart valves as mixed time varying boundary conditions. International Journal for Numerical Methods in Fluids, 85(3):135–164, 2017. ISSN 1097-0363. doi:10.1002/fld.4375. URL https://onlinelibrary.wiley.com/doi/abs/10.1002/fld.4375. _eprint: https://onlinelibrary.wiley.com/doi/pdf/10.1002/fld.4375.

[277] Karabelas Elias, Gsell Matthias A. F., Augustin Christoph M., Marx Laura, Neic Aurel, Prassl Anton J., Goubergrits Leonid, Kuehne Titus, and Plank Gernot. Towards a Computational Framework for Modeling the Impact of Aortic Coarctations Upon Left Ventricular Load. Frontiers in Physiology, 9:538, May 2018. ISSN 1664-042X. doi:10.3389/fphys.2018.00538. URL https://www.ncbi.nlm.nih.gov/pmc/articles/PMC5985756/. 29892227 PMC5985756

[278] Karabelas Elias, Longobardi Stefano, Fuchsberger Jana, Razeghi Orod, Rodero Cristobal, Strocchi Marina, Rajani Ronak, Haase Gundolf, Plank Gernot, and Niederer Steven. Global Sensitivity Analysis of Four Chamber Heart Hemodynamics Using Surrogate Models. IEEE Transactions on Biomedical Engineering, 69(10):3216–3223, October 2022. ISSN 1558-2531. doi:10.1109/TBME.2022.3163428. URL https://ieeexplore-ieee-org.stanford.idm.oclc.org/document/9745350. 35353691 PMC9491017

[279] Liu Ju and Marsden Alison L.. A unified continuum and variational multiscale formulation for fluids, solids, and fluid–structure interaction. Computer Methods in Applied Mechanics and Engineering, 337:549–597, August 2018. ISSN 0045-7825. doi:10.1016/j.cma.2018.03.045. URL https://www.sciencedirect.com/science/article/pii/S0045782518301701. 30505038 PMC6261472

[280] Liu Ju, Yang Weiguang, Lan Ingrid S., and Marsden Alison L.. Fluid-structure interaction modeling of blood flow in the pulmonary arteries using the unified continuum and variational multiscale formulation. Mechanics Research Communications, 107:103556, July 2020. ISSN 0093-6413. doi:10.1016/j.mechrescom.2020.103556. URL https://www.sciencedirect.com/science/article/pii/S0093641320300847. 32773906 PMC7405952

[281] Sun Yujie, Huang Jiayi, Lu Qingshuang, Yue Xinhai, Huang Xuanming, He Wei, Shi Yun, and Liu Ju. Modeling Fibrous Tissue in Vascular Fluid–Structure Interaction: A Morphology-Based Pipeline and Biomechanical Significance. International Journal for Numerical Methods in Biomedical Engineering, 41(1):e3892, 2025. ISSN 2040-7947. doi:10.1002/cnm.3892. URL https://onlinelibrary.wiley.com/doi/abs/10.1002/cnm.3892. _eprint: https://onlinelibrary.wiley.com/doi/pdf/10.1002/cnm.3892. 39725381

[282] Guan Jiashen, Yuan Hongyan, and Liu Ju. A structure-preserving integrator for incompressible finite elastodynamics based on a grad-div stabilized mixed formulation with particular emphasis on stretch-based material models. Computer Methods in Applied Mechanics and Engineering, 414:116145, September 2023. ISSN 0045-7825. doi:10.1016/j.cma.2023.116145. URL https://www.sciencedirect.com/science/article/pii/S0045782523002694.

[283] Liu Ju and Marsden Alison L.. A robust and efficient iterative method for hyper-elastodynamics with nested block preconditioning. Journal of Computational Physics, 383:72–93, April 2019. ISSN 0021-9991. doi:10.1016/j.jcp.2019.01.019. URL https://www.sciencedirect.com/science/article/pii/S0021999119300440. 31595091 PMC6781635

[284] Deuflhard Peter, Erdmann Bodo, Roitzsch Rainer, and Lines Glenn Terje. Adaptive finite element simulation of ventricular fibrillation dynamics. Computing and Visualization in Science, 12(5):201–205, June 2009. ISSN 1433-0369. doi:10.1007/s00791-008-0088-y. URL https://link.springer.com/article/10.1007/s00791-008-0088-y. Company: Springer Distributor: Springer Institution: Springer Label: Springer Number: 5 Publisher: Springer-Verlag.

[285] Buist Martin, Sands Gregory, Hunter Peter, and Pullan Andrew. A Deformable Finite Element Derived Finite Difference Method for Cardiac Activation Problems. Annals of Biomedical Engineering, 31(5):577–588, May 2003. ISSN 1573-9686. doi:10.1114/1.1567283. URL https://link.springer.com/article/10.1114/1.1567283. Company: Springer Distributor: Springer Institution: Springer Label: Springer Number: 5 Publisher: Kluwer Academic Publishers-Plenum Publishers. 12757201

[286] Chapelle Dominique, Fernández Miguel A., Gerbeau Jean-Frédéric, Moireau Philippe, Sainte-Marie Jacques, and Zemzemi Nejib. Numerical Simulation of the Electromechanical Activity of the Heart. In Functional Imaging and Modeling of the Heart, pages 357–365. Springer, Berlin, Heidelberg, 2009. ISBN 978-3-642-01932-6. doi:10.1007/978-3-642-01932-6_39. URL https://link.springer.com/chapter/10.1007/978-3-642-01932-6_39. ISSN: 1611–3349.

[287] Franzone Piero Colli, Pavarino Luca F., and Scacchi Simone. A Numerical Study of Scalable Cardiac Electro-Mechanical Solvers on HPC Architectures. Frontiers in Physiology, 9:268, April 2018. ISSN 1664-042X. doi:10.3389/fphys.2018.00268. URL https://www.ncbi.nlm.nih.gov/pmc/articles/PMC5895745/. 29674971 PMC5895745

[288] Spiteri Raymond J. and Dean Ryan C.. Stiffness Analysis of Cardiac Electrophysiological Models. Annals of Biomedical Engineering, 38(12):3592–3604, December 2010. ISSN 1573-9686. doi:10.1007/s10439-010-0100-9. URL https://link.springer.com/article/10.1007/s10439-010-0100-9. Company: Springer Distributor: Springer Institution: Springer Label: Springer Number: 12 Publisher: Springer US. 20582476

[289] Dal Hüsnü, Göktepe Serdar, Kaliske Michael, and Kuhl Ellen. A fully implicit finite element method for bidomain models of cardiac electromechanics. Computer Methods in Applied Mechanics and Engineering, 253:323–336, January 2013. ISSN 0045-7825. doi:10.1016/j.cma.2012.07.004. URL https://www.sciencedirect.com/science/article/pii/S0045782512002241. 23175588 PMC3501134

[290] Wong J., Göktepe S., and Kuhl E.. Computational modeling of chemo-electro-mechanical coupling: A novel implicit monolithic finite element approach. International journal for numerical methods in biomedical engineering, 29(10):1104–1133, October 2013. ISSN 2040-7939. doi:10.1002/cnm.2565. URL https://www.ncbi.nlm.nih.gov/pmc/articles/PMC4567385/. 23798328 PMC4567385

[291] Gerbi Antonello, Dedè Luca, Quarteroni Alfio, Gerbi Antonello, Dedè Luca, and Quarteroni Alfio. A monolithic algorithm for the simulation of cardiac electromechanics in the human left ventricle. Mathematics in Engineering, 1(1):1–37, 2019. ISSN 2640-3501. doi:10.3934/Mine.2018.1.1. URL http://www.aimspress.com/article/doi/10.3934/Mine.2018.1.1. Cc_license_type: cc_by Number: Mine-01-01-001 Primary_atype: Mathematics in Engineering Subject_term: Research article Subject_term_id: Research article.

[292] Sundnes J., Wall S., Osnes H., Thorvaldsen T., and McCulloch A.D.. Improved discretisation and linearisation of active tension in strongly coupled cardiac electro-mechanics simulations. Computer methods in biomechanics and biomedical engineering, 17(6):604–615, 2014. ISSN 1025-5842. doi:10.1080/10255842.2012.704368. URL https://www.ncbi.nlm.nih.gov/pmc/articles/PMC4230300/. 22800534 PMC4230300

[293] Krishnamoorthi Shankarjee, Sarkar Mainak, and Klug William S.. Numerical Quadrature and Operator Splitting in Finite Element Methods for Cardiac Electrophysiology. International journal for numerical methods in biomedical engineering, 29(11):1243–1266, November 2013. ISSN 2040-7939. doi:10.1002/cnm.2573. URL https://www.ncbi.nlm.nih.gov/pmc/articles/PMC4519349/. 23873868 PMC4519349

[294] Pitt-Francis Joe, Pathmanathan Pras, Bernabeu Miguel O., Bordas Rafel, Cooper Jonathan, Fletcher Alexander G., Mirams Gary R., Murray Philip, Osborne James M., Walter Alex, Chapman S. Jon, Garny Alan, van Leeuwen Ingeborg M. M., Maini Philip K., Rodríguez Blanca, Waters Sarah L., Whiteley Jonathan P., Byrne Helen M., and Gavaghan David J.. Chaste: A test-driven approach to software development for biological modelling. Computer Physics Communications, 180(12):2452–2471, December 2009. ISSN 0010-4655. doi:10.1016/j.cpc.2009.07.019. URL https://www.sciencedirect.com/science/article/pii/S0010465509002604.

[295] Africa Pasquale Claudio. lifex: A flexible, high performance library for the numerical solution of complex finite element problems. SoftwareX, 20:101252, December 2022. ISSN 2352-7110. doi:10.1016/j.softx.2022.101252. URL https://www.sciencedirect.com/science/article/pii/S2352711022001704.

[296] Arndt Daniel, Bangerth Wolfgang, Blais Bruno, Clevenger Thomas C, Fehling Marc, Grayver Alexander V, Heister Timo, Heltai Luca, Kronbichler Martin, Munch Peter, Maier Matthias, Pelteret Jean-Paul, Rastak Reza, Turcksin Bruno, Wang Zhuoran, and Wells David. The deal.II Library, Version 9.2, 2020.

[297] Africa Pasquale Claudio, Piersanti Roberto, Fedele Marco, Dede’ Luca, and Quarteroni Alfio. lifex-fiber: an open tool for myofibers generation in cardiac computational models. BMC Bioinformatics, 24(1):143, April 2023. ISSN 1471-2105. doi:10.1186/s12859-023-05260-w. URL https://doi.org/10.1186/s12859-023-05260-w. 37046208 PMC10091584

[298] Pasquale Claudio Africa Ivan Fumagalli, Bucelli Michele, Zingaro Alberto, Fedele Marco, Dede’ Luca, and Quarteroni Alfio. lifex-cfd: An open-source computational fluid dynamics solver for cardiovascular applications. Computer Physics Communications, 296:109039, March 2024. ISSN 0010-4655. doi:10.1016/j.cpc.2023.109039. URL https://www.sciencedirect.com/science/article/pii/S0010465523003843.

[299] Zhu Chi, Vedula Vijay, Parker Dave, Wilson Nathan, Shadden Shawn, and Marsden Alison. svFSI: A Multiphysics Package for Integrated Cardiac Modeling. Journal of Open Source Software, 7(78):4118, October 2022. ISSN 2475-9066. doi:10.21105/joss.04118. URL https://joss.theoj.org/papers/10.21105/joss.04118.

[300] The Trilinos Project Team. The Trilinos Project Website.

[301] Balay S., Abhyankar S., Adams M., Brown J., Brune P., Buschelman K., Dalcin L., Dener A., Eijkhout V., Gropp W., Karpeyev D., Kaushik D., Knepley M., May D., McInnes L., Mills R., Munson T., Rupp K., Sanan P., Smith B., Zampini S., and Zhang H.. PETSc Users Manual. 2019. URL https://ora.ox.ac.uk/objects/uuid:fa2b9e7c-1c58-429c-90fd-f780a3c3dc7d. Publisher: Argonne National Laboratory.

[302] Lee J., Cookson A., Roy I., Kerfoot E., Asner L., Vigueras G., Sochi T., Deparis S., Michler C., Smith N. P., and Nordsletten D. A.. Multiphysics Computational Modeling in $\boldsymbol{\mathcal{C}}\mathbf{Heart}$. SIAM Journal on Scientific Computing, 38(3):C150–C178, January 2016. ISSN 1064-8275. doi:10.1137/15M1014097. URL https://epubs.siam.org/doi/10.1137/15M1014097. Publisher: Society for Industrial and Applied Mathematics.

[303] 4C. 4C: A Comprehensive Multiphysics Simulation Framework. URL https://www.4c-multiphysics.org.

[304] Hirschvogel Marc. Ambit – A FEniCS-based cardiovascular multi-physicssolver. Journal of Open Source Software, 9(93):5744, January 2024. ISSN 2475-9066. doi:10.21105/joss.05744. URL https://joss.theoj.org/papers/10.21105/joss.05744.

[305] Maas Steve A., Ellis Benjamin J., Ateshian Gerard A., and Weiss Jeffrey A.. FEBio: Finite Elements for Biomechanics. Journal of Biomechanical Engineering, 134(011005), February 2012. ISSN 0148-0731. doi:10.1115/1.4005694. URL https://doi.org/10.1115/1.4005694. PMC3705975 22482660

[306] Plank Gernot, Loewe Axel, Neic Aurel, Augustin Christoph, Huang Yung-Lin, Gsell Matthias A. F., Karabelas Elias, Nothstein Mark, Prassl Anton J., Sánchez Jorge, Seemann Gunnar, and Vigmond Edward J.. The *openCARP* simulation environment for cardiac electrophysiology. Computer Methods and Programs in Biomedicine, 208:106223, September 2021. ISSN 0169-2607. doi:10.1016/j.cmpb.2021.106223. URL https://www.sciencedirect.com/science/article/pii/S0169260721002972. 34171774

[307] Lasso Andras, Herz Christian, Nam Hannah, Cianciulli Alana, Pieper Steve, Drouin Simon, Pinter Csaba, St-Onge Samuelle, Vigil Chad, Ching Stephen, Sunderland Kyle, Fichtinger Gabor, Kikinis Ron, and Jolley Matthew A.. SlicerHeart: An open-source computing platform for cardiac image analysis and modeling. Frontiers in Cardiovascular Medicine, 9, September 2022. ISSN 2297-055X. doi:10.3389/fcvm.2022.886549. URL https://www.frontiersin.org/journals/cardiovascular-medicine/articles/10.3389/fcvm.2022.886549/full. Publisher: Frontiers. PMC9485637 36148054

[308] 3D Slicer image computing platform,. URL https://slicer.org/. 10.1016/j.mri.2012.05.001 PMC3466397 22770690

[309] Viola Francesco, Del Corso Giulio, Ruggero, and Verzicco Roberto. GPU accelerated digital twins of the human heart open new routes for cardiovascular research. Scientific Reports, 13(1):8230, May 2023. ISSN 2045-2322. doi:10.1038/s41598-023-34098-8. URL https://www.nature.com/articles/s41598-023-34098-8. Publisher: Nature Publishing Group. 37217483 PMC10203142

[310] Hurtado Daniel E. and Zavala Patricio. Accelerating cardiac and vessel mechanics simulations: An energy-transform variational formulation for soft-tissue hyperelasticity. Computer Methods in Applied Mechanics and Engineering, 379:113764, June 2021. ISSN 0045-7825. doi:10.1016/j.cma.2021.113764. URL https://www.sciencedirect.com/science/article/pii/S0045782521001006.

[311] Buoso Stefano, Joyce Thomas, and Kozerke Sebastian. Personalising left-ventricular biophysical models of the heart using parametric physics-informed neural networks. Medical Image Analysis, 71:102066, July 2021. ISSN 1361-8415. doi:10.1016/j.media.2021.102066. URL https://www.sciencedirect.com/science/article/pii/S1361841521001122. 33951597

[312] Cicci Ludovica, Fresca Stefania, Manzoni Andrea, and Quarteroni Alfio. Efficient approximation of cardiac mechanics through reduced-order modeling with deep learning-based operator approximation. International Journal for Numerical Methods in Biomedical Engineering, 40(1):e3783, 2024. ISSN 2040-7947. doi:10.1002/cnm.3783. URL https://onlinelibrary.wiley.com/doi/abs/10.1002/cnm.3783. _eprint: https://onlinelibrary.wiley.com/doi/pdf/10.1002/cnm.3783. 37921217

[313] Dabiri Yaghoub, Van der Velden Alex, Sack Kevin L., Choy Jenny S., Guccione Julius M., and s Ghassan S.. Application of feed forward and recurrent neural networks in simulation of left ventricular mechanics. Scientific Reports, 10(1):22298, December 2020. ISSN 2045-2322. doi:10.1038/s41598-020-79191-4. URL https://www.nature.com/articles/s41598-020-79191-4. Publisher: Nature Publishing Group. 33339836 PMC7749109

[314] Gonzalo D. Talou Maso, Babarenda Gamage Thiranja P., Sagar Mark, and Nash Martyn P.. Deep Learning Over Reduced Intrinsic Domains for Efficient Mechanics of the Left Ventricle. Frontiers in Physics, 8, February 2020. ISSN 2296-424X. doi:10.3389/fphy.2020.00030. URL https://www.frontiersin.org/journals/physics/articles/10.3389/fphy.2020.00030/full. Publisher: Frontiers.

[315] Regazzoni F. and Quarteroni A.. Accelerating the convergence to a limit cycle in 3D cardiac electromechanical simulations through a data-driven 0D emulator. Computers in Biology and Medicine, 135:104641, August 2021. ISSN 0010-4825. doi:10.1016/j.compbiomed.2021.104641. URL https://www.sciencedirect.com/science/article/pii/S0010482521004352. 34298436

[316] Regazzoni F., Salvador M., Dede’ L., and Quarteroni A.. A machine learning method for real-time numerical simulations of cardiac electromechanics. Computer Methods in Applied Mechanics and Engineering, 393: 114825, April 2022. ISSN 0045-7825. doi:10.1016/j.cma.2022.114825. URL https://www.sciencedirect.com/science/article/pii/S004578252200144X.

[317] Salvador Matteo, Strocchi Marina, Regazzoni Francesco, Augustin Christoph M., Dede’ Luca, Niederer Steven A., and Quarteroni Alfio. Whole-heart electromechanical simulations using Latent Neural Ordinary Differential Equations. npj Digital Medicine, 7(1):1–8, April 2024. ISSN 2398-6352. doi:10.1038/s41746-024-01084-x. URL https://www.nature.com/articles/s41746-024-01084-x. Publisher: Nature Publishing Group. 38605089 PMC11009296

[318] Motiwale Shruti, Zhang Wenbo, Feldmeier Reese, and Sacks Michael S.. A neural network finite element approach for high speed cardiac mechanics simulations. Computer Methods in Applied Mechanics and Engineering, 427:117060, July 2024. ISSN 0045-7825. doi:10.1016/j.cma.2024.117060. URL https://www.sciencedirect.com/science/article/pii/S0045782524003165.

[319] Ambrosi Davide, Ben Amar Martine, Cyron Christian J., DeSimone Antonio, Goriely Alain, Humphrey Jay D., and Kuhl Ellen. Growth and remodelling of living tissues: perspectives, challenges and opportunities. Journal of The Royal Society Interface, 16(157):20190233, August 2019. doi:10.1098/rsif.2019.0233. URL https://royalsocietypublishing.org/doi/10.1098/rsif.2019.0233. Publisher: Royal Society. PMC6731508 31431183

[320] Lee Yongjae, Cansız Barış, and Kaliske Michael. A multiphysical computational model of myocardial growth adopted to human pathological ventricular remodelling. Computational Mechanics, 72(6):1215–1237, December 2023. ISSN 1432-0924. doi:10.1007/s00466-023-02346-3. URL https://doi.org/10.1007/s00466-023-02346-3.

[321] Gebauer Amadeus M., Pfaller Martin R., Braeu Fabian A., Cyron Christian J., and Wall Wolfgang A.. A homogenized constrained mixture model of cardiac growth and remodeling: analyzing mechanobiological stability and reversal. Biomechanics and Modeling in Mechanobiology, 22(6):1983–2002, December 2023. ISSN 1617-7940. doi:10.1007/s10237-023-01747-w. URL https://link.springer.com/article/10.1007/s10237-023-01747-w. Company: Springer Distributor: Springer Institution: Springer Label: Springer Number: 6 Publisher: Springer Berlin Heidelberg. 37482576 PMC10613155

[322] Schwarz Erica L., Pfaller Martin R., Szafron Jason M., Latorre Marcos, Lindsey Stephanie E., Breuer Christopher K., Humphrey Jay D., and Marsden Alison L.. A fluid–solid-growth solver for cardiovascular modeling. Computer Methods in Applied Mechanics and Engineering, 417:116312, December 2023. ISSN 0045-7825. doi:10.1016/j.cma.2023.116312. URL https://www.sciencedirect.com/science/article/pii/S004578252300436X. PMC10691594 38044957

[323] Guan Debao, Zhuan Xin, Luo Xiaoyu, and Gao Hao. An updated Lagrangian constrained mixture model of pathological cardiac growth and remodelling. Acta Biomaterialia, 166:375–399, August 2023. ISSN 1742-7061. doi:10.1016/j.actbio.2023.05.022. URL https://www.sciencedirect.com/science/article/pii/S1742706123002787. 37201740

[324] Holzapfel Gerhard A., Humphrey Jay D., and Ogden Ray W.. Biomechanics of soft biological tissues and organs, mechanobiology, homeostasis and modelling. Journal of The Royal Society Interface, 22(222):20240361, January 2025. doi:10.1098/rsif.2024.0361. URL https://royalsocietypublishing.org/doi/10.1098/rsif.2024.0361. Publisher: Royal Society. PMC11775666 39876788

[325] Borowska Agnieszka, Gao Hao, Lazarus Alan, and Husmeier Dirk. Bayesian optimisation for efficient parameter inference in a cardiac mechanics model of the left ventricle. International Journal for Numerical Methods in Biomedical Engineering, 38(5):e3593, 2022. ISSN 2040-7947. doi:10.1002/cnm.3593. URL https://onlinelibrary.wiley.com/doi/abs/10.1002/cnm.3593. _eprint: https://onlinelibrary.wiley.com/doi/pdf/10.1002/cnm.3593. 35302293 PMC9285944

[326] Miller Renee, Kerfoot Eric, Mauger Charlène, Ismail Tevfik F., Young Alistair A., and Nordsletten David A.. An Implementation of Patient-Specific Biventricular Mechanics Simulations With a Deep Learning and Computational Pipeline. Frontiers in Physiology, 12, September 2021. ISSN 1664-042X. doi:10.3389/fphys.2021.716597. URL https://www.frontiersin.org/journals/physiology/articles/10.3389/fphys.2021.716597/full. Publisher: Frontiers. PMC8481785 34603077

[327] Salvador Matteo, Kong Fanwei, Peirlinck Mathias, Parker David W., Chubb Henry, Dubin Anne M., and Marsden Alison L.. Digital twinning of cardiac electrophysiology for congenital heart disease. Journal of The Royal Society Interface, 21(215):20230729, June 2024. doi:10.1098/rsif.2023.0729. URL https://royalsocietypublishing.org/doi/full/10.1098/rsif.2023.0729. Publisher: Royal Society. PMC11285762 38835246

[328] Wang Zhinuo Jenny, Holmes Maxx, Doste Ruben, Camps Julia, Margara Francesca, Vazquez Mariano, and Rodriguez Blanca. Calibration and validation strategy for electromechanical cardiac digital twins, March 2025. URL https://www.biorxiv.org/content/10.1101/2025.03.06.638897v1. Pages: 2025.03.06.638897 Section: New Results.

[329] Schwarz Erica L., Pegolotti Luca, Pfaller Martin R., and Marsden Alison L.. Beyond CFD: Emerging methodologies for predictive simulation in cardiovascular health and disease. Biophysics Reviews, 4(1):011301, January 2023. ISSN 2688-4089. doi:10.1063/5.0109400. URL https://doi.org/10.1063/5.0109400. PMC9846834 36686891

[330] Valen-Sendstad Kristian, Bergersen Aslak W., Shimogonya Yuji, Goubergrits Leonid, Bruening Jan, Pallares Jordi, Cito Salvatore, Piskin Senol, Pekkan Kerem, Geers Arjan J., Larrabide Ignacio, Rapaka Saikiran, Mihalef Viorel, Fu Wenyu, Qiao Aike, Jain Kartik, Roller Sabine, Mardal Kent-Andre, Kamakoti Ramji, Spirka Thomas, Ashton Neil, Revell Alistair, Aristokleous Nicolas, Houston J. Graeme, Tsuji Masanori, Ishida Fujimaro, Menon Prahlad G., Browne Leonard D., Broderick Stephen, Shojima Masaaki, Koizumi Satoshi, Barbour Michael, Aliseda Alberto, Morales Hernán G., Lefèvre Thierry, Hodis Simona, Al-Smadi Yahia M., Tran Justin S., Marsden Alison L., Vaippummadhom Sreeja, Einstein G. Albert, Brown Alistair G., Debus Kristian, Niizuma Kuniyasu, Rashad Sherif, Sugiyama Shin-Ichiro, Khan M. Owais, Updegrove Adam R., Shadden Shawn C., Cornelissen Bart M. W., Majoie Charles B. L. M., Berg Philipp, Saalfield Sylvia, Kono Kenichi, and Steinman David A.. Real-World Variability in the Prediction of Intracranial Aneurysm Wall Shear Stress: The 2015 International Aneurysm CFD Challenge. Cardiovascular Engineering and Technology, 9(4):544–564, December 2018. ISSN 1869-4098. doi:10.1007/s13239-018-00374-2. 30203115 PMC6290689

